# Bioassay-Guided Isolation of *cis*-Clerodane Diterpenoids and Monoglycerides from the Leaves of *Solidago gigantea* and Their Antimicrobial Activities

**DOI:** 10.3390/plants14142152

**Published:** 2025-07-11

**Authors:** Márton Baglyas, Péter G. Ott, Zoltán Bozsó, Ildikó Schwarczinger, József Bakonyi, Dénes Dlauchy, András Darcsi, Szilárd Varga, Ágnes M. Móricz

**Affiliations:** 1 Plant Protection Institute, HUN-REN Centre for Agricultural Research, Fehérvári út 132-144, 1116 Budapest, Hungary; baglyas.marton@atk.hun-ren.hu (M.B.); ott.peter@atk.hun-ren.hu (P.G.O.); bozso.zoltan@atk.hun-ren.hu (Z.B.); schwarczinger.ildiko@atk.hun-ren.hu (I.S.); bakonyi.jozsef@atk.hun-ren.hu (J.B.); 2 Doctoral School, Semmelweis University, Üllői út 26, 1085 Budapest, Hungary; 3 National Collection of Agricultural and Industrial Microorganisms, Institute of Food Science and Technology, Hungarian University of Agriculture and Life Sciences, Somlói út 14-16, 1118 Budapest, Hungary; dlauchy.denes@uni-mate.hu; 4 Pharmaceutical Chemistry and Technology Department, National Center for Public Health and Pharmacy, Szabolcs utca 33, 1135 Budapest, Hungary; darcsi.andras@nngyk.gov.hu; 5 Organocatalysis Research Group, Institute of Organic Chemistry, HUN-REN Research Centre for Natural Sciences, Magyar Tudósok krt. 2, 1117 Budapest, Hungary; varga.szilard@ttk.hu

**Keywords:** giant goldenrod, solidagoic acid J, TLC hyphenations, TLC–direct bioautography, effect-directed analysis, bioassays, antibacterial activity, antifungal activity

## Abstract

A previously undescribed *cis*-clerodane diterpenoid, diangelate solidagoic acid J (**1**), along with two known *cis*-clerodane diterpenoids, solidagoic acid C (**2**) and solidagoic acid D (**3**), as well as two known unsaturated monoacylglycerols, 1-linoleoyl glycerol (**4**) and 1-α-linolenoyl glycerol (**5**), were isolated and characterized from the *n*-hexane leaf extract of *Solidago gigantea* (giant goldenrod). Compounds **2**–**5** were identified first in this species, and compounds **4** and **5** are reported here for the first time in the *Solidago* genus. The bioassay-guided isolation procedure included thin-layer chromatography (TLC) coupled with a *Bacillus subtilis* antibacterial assay, preparative flash column chromatography, and TLC–mass spectrometry (MS). Their structures were elucidated via extensive spectroscopic and spectrometric techniques such as one- and two-dimensional nuclear magnetic resonance (NMR) spectroscopy and high-resolution tandem mass spectrometry (HRMS/MS). The antimicrobial activities of the isolated compounds were evaluated by a microdilution assay. All compounds exhibited weak to moderate antibacterial activity against the Gram-positive plant pathogen *Clavibacter michiganensis*, with MIC values ranging from 17 to 133 µg/mL, with compound **5** being the most potent. Only compound **1** was active against *Curtobacterium flaccumfaciens* pv. *flaccumfaciens*, while compound **3** demonstrated a weak antibacterial effect against *B. subtilis* and *Rhodococcus fascians*. Additionally, the growth of *B. subtilis* and *R. fascians* was moderately inhibited by compounds **1** and **5**, respectively. None of the tested compounds showed antibacterial activity against Gram-negative *Pseudomonas syringae* pv. *tomato* and *Xanthomonas arboricola* pv. *pruni*. No bactericidal activity was observed against the tested microorganisms. Compounds **2** and **3** displayed weak antifungal activity against the crop pathogens *Bipolaris sorokiniana* and *Fusarium graminearum*. Our results demonstrate the efficacy of bioassay-guided strategies in facilitating the discovery of novel bioactive compounds.

## 1. Introduction

*Solidago*, commonly referred to as goldenrod, is a large genus within the Asteraceae family, comprising approximately 139 species [1]. *S. virgaurea* L. is native to Europe. The other goldenrods originate primarily from North America, although four of their representatives have naturalized in Europe: *S. gigantea* Ait., *S. canadensis* L., *S. altissima* L., and *Euthamia graminifolia* (L.) Nutt. (previously known as *S. graminifolia* (L.) Salisb.) [2].

**Figure 1:**
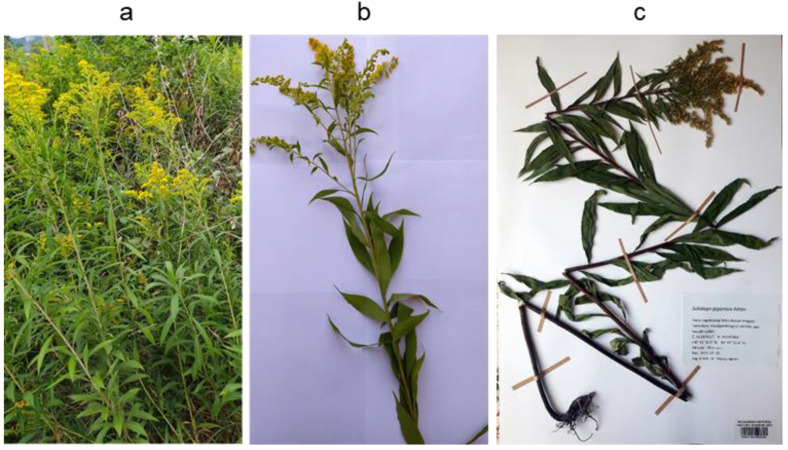
Illustration of the invasive spread of *Solidago gigantea* Ait. (giant goldenrod) in its natural habitat (**a**), the morphological features of its aerial parts (**b**), and the corresponding voucher herbarium specimen (accession number: HNHM-TRA 00027284) (**c**).

*S. gigantea* Ait. (giant goldenrod) (Figure 1a–c) is a rhizomatous perennial plant that has become a well-known, notorious, and highly invasive species throughout Europe, where its aggressive spread presents a major challenge for biodiversity conservation [3,4]. Among the factors contributing to its successful invasion is its allelopathic potential that enables the plant to suppress the growth of native species by releasing allelochemicals [5]. It is also recognized as a medicinal plant; its aerial, flowering parts (combined with *S. canadensis* and *S. virgaurea*) are officially listed as *Solidaginis herba* in the European Pharmacopoeia [6] and are traditionally used in phytotherapy for their anti-inflammatory, diuretic, and spasmolytic properties [7]. Invasive *Solidago* weeds, such as *S. gigantea*, have attracted considerable scientific attention not only due to their ecological impact but also as potential sources of novel bioactive specialized metabolites. The extracts or essential oils obtained from *S. gigantea* aerial parts exhibit various biological activities, including antibacterial [8], antifungal [9], antimutagenic [6,8], antiproliferative [10], antioxidant [6], and insecticidal [11] effects. These pharmacological activities have been attributed to flavonoids [12,13,14], phenolic acids [12,13,14], monoterpenoids [15,16], sesquiterpenoids [15,16], diterpenoids [17,18,19], and triterpenoids [20,21,22,23].

In our previous work, four antibacterial *cis*-clerodane diterpenoids, solidagoic acids E, F, H, and I, were isolated from an *n*-hexane leaf extract of *S. gigantea* [19]. Encouraged by these findings, our ongoing search for bioactive compounds derived from the invasive goldenrod species was continued [24,25,26,27].

Thus, this work presents non-targeted, effect-directed screening and subsequent highly targeted bioassay-guided fractionation and isolation, followed by the structure elucidation, comprehensive spectroscopic and spectrometric characterization, and microbiological evaluation of compounds contributing to the antibacterial effect of the *n*-hexane leaf extract of *S. gigantea*. The methodologies employed included TLC, TLC–direct bioautography (using a *Bacillus subtilis* antibacterial assay), TLC–MS, preparative flash column chromatography, flow injection analysis (FIA)–heated electrospray ionization (HESI)-tandem high-resolution MS (HRMS/MS), polarimetry, and nuclear magnetic resonance (NMR), UV, and attenuated total reflectance Fourier-transform infrared (ATR–FTIR) spectroscopy. The antibacterial and antifungal activities of the isolated compounds were assessed by in vitro microplate assays against the non-pathogenic soil bacterium *B. subtilis* and the phytopathogenic bacteria *Curtobacterium flaccumfaciens* pv. *flaccumfaciens*, *Clavibacter michiganensis*, *Pseudomonas syringae* pv. *tomato*, *Rhodococcus fascians*, and *Xanthomonas arboricola* pv. *pruni*, as well as the plant pathogenic fungal strains *Bipolaris sorokiniana* and *Fusarium graminearum*.

## 2. Results and Discussion

### 2.1. Bioassay-Guided Isolation

**Figure 2:**
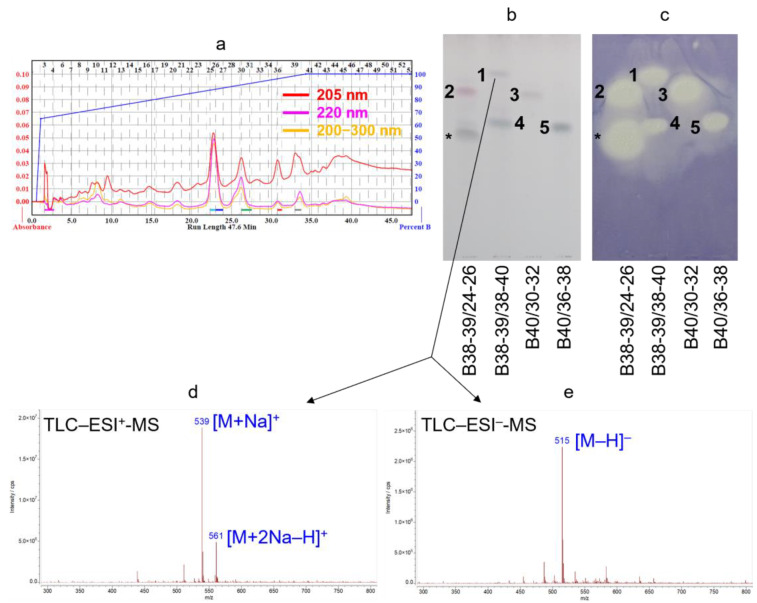
UV chromatograms (**a**) recorded during the preparative flash chromatographic fractionation of fraction B38-39 using a C_18_ column as a stationary phase and water with 0.1% formic acid–methanol gradient as a mobile phase. TLC chromatograms (**b**) and bioautograms (**c**) of flash subfractions B38-39/24-26, B38-39/38-40, B40/30-32, and B40/36-38 with compounds **1**–**5**, visualized after derivatization with *p*-anisaldehyde reagent (**b**) and evaluated via direct bioautography using a *Bacillus subtilis* antibacterial assay (**c**). *R*_F_ values for compounds **1**–**5**: 0.81 (**1**), 0.73 (**2**), 0.71 (**3**), 0.59 (**4**), and 0.58 (**5**). Compounds responsible for the inhibition zone detected in subfraction B38-39/24-26, marked with an asterisk sign (*) (**b**,**c**), have not been isolated. TLC–ESI^+^-MS (**d**) and TLC–ESI^−^-MS (**e**) spectra of compound **1** in subfraction B38-39/38-40, recorded using an elution-head based interface followed by a background subtraction, are exemplarily shown along with the assignments.

In our preliminary experiments, the *n*-hexane leaf extract of *S. gigantea* displayed antibacterial activity against *B. subtilis*, observed by an in vitro microdilution assay. To detect potential antibacterial compounds, the *n*-hexane leaf extract was subjected to a non-targeted, effect-directed screening using TLC–direct bioautography with a *B. subtilis* antibacterial assay [19]. Four *cis*-clerodane diterpenoids responsible for the antibacterial effect were identified but the investigation was not exhaustive and focused on flash fractions B43–45 and B46–48, containing slightly more polar compounds compared to this work [19]. Therefore, to identify further bioactive components, the *n*-hexane leaf extract was subjected to repeated fractionation by preparative flash chromatography (Figure 2a) using normal- (silica gel) and reversed-phase (C_18_) columns, ultimately returning to silica gel column chromatography, to exploit the orthogonal selectivity of these stationary phases. The purification procedure was continuously monitored by TLC with *p*-anisaldehyde derivatization (Figure 2b), TLC–*B. subtilis* direct bioautography (Figure 2c), TLC–MS (Figure 2d,e), and RP-HPLC–ESI-MS. Fractions with similar TLC fingerprints were pooled. The isolation process resulted in five antibacterial compounds obtained from flash fractions B38–39 and B40 (**1**–**5**): 0.8 mg of compound **1** (white amorphous solid), 10.7 mg of compound **2** (white amorphous solid), 9.3 mg of compound **3** (white amorphous solid), 2.4 mg of compound **4** (yellow oil), and 2.1 mg of compound **5** (yellow oil).

With the TLC mobile phase chloroform–ethyl acetate–methanol 15:3:2 (*V*/*V*), compound **1** exhibited an *R*_F_ value of 0.81, compounds **2** and **3** migrated with an *R*_F_ value of 0.73 and 0.71, respectively, and compounds **4** and **5** migrated with an *R*_F_ value of 0.59 and 0.58, respectively. After derivatization with *p*-anisaldehyde, compounds **2** and **3** appeared as pink spots, while compounds **1**, **4**, and **5** were visualized as dark purple zones on the TLC plates (Figure 2b). All isolated compounds demonstrated strong inhibition zones in the TLC–*B. subtilis* antibacterial assay (Figure 2c). Note that compounds responsible for the inhibition zone detected in the subfraction B38-39/24-26, marked with an asterisk sign (*) (Figure 2b,c), have not been isolated.

### 2.2. Structure Elucidation

In this study, one previously undescribed *cis*-clerodane diterpenoid (**1**) along with two known *cis*-clerodane diterpenoids (**2**–**3**) and two known, unsaturated monoglycerides (**4**–**5**) (Figure 3) were isolated. Their structures were elucidated by extensive spectroscopic and spectrometric techniques and comparison with previously reported data. The novelty of the structure of **1** was verified by searching in CAS SciFinder^®^ and Reaxys databases. The recorded one- and two-dimensional NMR (Figures S1–S12 for **1**, Figures S19–S25 for **2**, Figures S30–S36 for **3**, Figures S41–S46 for **4**, and Figures S49–S54 for **5**), HR-ESI-MS(/MS) (Figures S13–S16 for **1**, Figures S26–S29 for **2**, Figures S37–S40 for **3**, Figures S47 and S48 for **4**, and Figures S55 and S56 for **5**), UV (Figure S17 for **1**), and ATR-FTIR spectra (Figure S18 for **1**) of compounds **1**–**5** can be found in the Supporting Information.

Solidagoic acid J (**1**) was obtained as a white amorphous solid. Its molecular formula was established as C_30_H_44_O_7_ deduced from the ^13^C DEPTQ NMR spectrum and based on the positive- and negative-ion mode HR-ESI-MS signals at *m*/*z* 539.2980 [M+Na]^+^ (calculated for C_30_H_44_O_7_Na^+^, *m*/*z* 539.2979 [M+Na]^+^; error: 0.1 ppm) and at *m*/*z* 515.3013 [M−H]^−^ (calculated for C_30_H_43_O_7_^−^, *m*/*z* 515.3014 [M−H]^−^; error: −0.3 ppm), indicating nine double bond equivalents (DBEs). Its ^1^H NMR spectrum (Table 1) revealed proton resonances corresponding to six methyl groups at *δ*_H_ 1.97 (ov., 6H, H_3_-4′, H_3_-4″), 1.88 (ov., 3H, H_3_-5′), 1.86 (ov., 3H, H_3_-5″), and 0.92 (s, 3H, H_3_-20), 0.79 (d, *J* = 6.4 Hz, H_3_-17), four vinylic hydrogens at *δ*_H_ 6.10 (qq, *J* = 7.2, 1.3 Hz, 1H, H-3″), 6.04 (qq, *J* = 7.2, 1.3 Hz, 1H, H-3′), 5.91 (t, *J* = 4.0 Hz, 1H, H-3), and 5.72 (t, *J* = 7.1 Hz, 1H, H-14), and three oxymethylene groups at *δ*_H_ 4.80 (d, *J* = 12.3 Hz, 1H, H_2_-16a), 4.66 (d, *J* = 12.3 Hz, 1H, H_2_-16b), 4.51 (m, 2H, H_2_-18), and 4.26 (d, *J* = 7.1 Hz, 2H, H_2_-15). Based on ^13^C (Table 1), ^1^H–^13^C multiplicity-edited HSQC (edHSQC), and ^1^H–^13^C HMBC spectroscopic data, the thirty ^13^C resonances were assigned to six methyl groups (*δ*_C_ 27.0, 20.8, 20.7, 16.0, 15.9, 15.8), six aliphatic methylene (*δ*_C_ 30.1, 30.0, 29.4, 28.0, 26.5, 19.6) and three oxygenated (*δ*_C_ 64.5, 61.7, 58.7) methylene groups, two aliphatic methine (*δ*_C_ 42.6, 37.0) and four olefinic (*δ*_C_ 139.3, 138.3, 128.7, 128.1) methine groups, and nine non-hydrogenated carbons, including one carboxylic (*δ*_C_ 178.8), two aliphatic (*δ*_C_ 50.0, 38.7), two ester (*δ*_C_ 168.7, 167.7), and four olefinic (*δ*_C_ 139.1, 136.2, 128.0, 127.7) carbons. Thus, the structure of compound **1** exhibited one carboxylic, two ester moieties, and four trisubstituted carbon–carbon double bonds, implying a bicyclic molecule to satisfy the required number of DBEs. In addition, 42 hydrogen atoms could be accounted for, and the remaining two exchangeable hydrogens were assigned to one carboxylic and one hydroxylic group. The ATR-FTIR spectrum featured characteristic, intense bands at 1715 cm^−1^ (overlapping C=O stretches) and 1648 cm^−1^ (C=C stretch). These NMR spectroscopic data suggested that compound **1** possessed a clerodane-type diterpene skeleton featuring two methyl groups and two angeloyloxy moieties.

The ^1^H–^1^H COSY and ^1^H–^1^H TOCSY spectra revealed six distinct spin systems: H-10/H_2_-1/H_2_-2/H-3, H_2_-6/H_2_-7/H-8/H_3_-17, H_2_-11/H_2_-12, H-14/H_2_-15, H-3′/H_3_-4′, and H-3″/H_3_-4″ (Figure 4). The 6/6 fused A/B ring system (C-1–C-10) bearing two methyl groups (C-17, C-20) and two modified methyl groups (C-18, C-19) was constructed from the spin systems H-10/H_2_-1/H_2_-2/H-3 and H_2_-6/H_2_-7/H-8/H_3_-17, and ^1^H–^13^C HMBC correlations from H-3 to C-5, from H-10 to C-5, C-6, C-8, and C-9, from H_3_-17 to C-7, C-8, and C-9, from H_2_-18 to C-3, C-4, and C-5, and from H_3_-20 to C-8, C-9, and C-10 (Figure 4). The carboxylic group was assigned to C-5, supported by the HMBC correlations from H_2_-6a, H_2_-6b, and H-10 to C-19. The structure of the side chain composed of C-11–C-16 was established by the spin systems H_2_-11/H_2_-12 and H-14/H_2_-15, as well as the HMBC correlations from H_2_-11 to C-13, from H_2_-12 to C-13, C-14, and C-16, from H-14 to C-13, C-12, and C-16, and from H_2_-16 to C-12, C-13, and C-14. The hydroxy group was attached to C-15 as indicated by the downfield ^1^H and ^13^C NMR chemical shift of H_2_-15 (*δ*_H_ 4.26) and C-15 (*δ*_C_ 58.7). The side chain unit was connected to C-9 as inferred from the HMBC correlations from H_2_-11 to C-9, C-10, and C-20 and from H-8, H-10, and H_3_-20 to C-11. The presence of two angeloyloxy (Ang) groups was confirmed by the spin systems H-3′/H_3_-4′ and H-3″/H_3_-4″ along with HMBC correlations from H-3′ to C-1′, from H_3_-4′ to C-2′, from H_3_-5′ to C-1′, C-2′, and C-3′, from H-3″ to C-1″, from H_3_-4″ to C-2″, and from H_3_-5″ to C-1″, C-2″, and C-3″. This was further verified by the MS fragmentation of the precursor ion at *m*/*z* 539.2979 [M+Na]^+^, which resulted in the formation of fragment ions at *m*/*z* 439.2454 [M+Na–C_5_H_8_O_2_]^+^ and at *m*/*z* 339.1930 [M+Na–C_10_H_16_O_4_]^+^, indicating the successive losses of two Ang groups (C_5_H_8_O_2_). Additionally, the MS fragmentation of the parent ion at *m*/*z* 515.3014 [M−H]^−^ yielded a product ion at *m*/*z* 99.0452, corresponding to an angelate ion (C_5_H_7_O_2_^−^). The two angeloyloxy groups were linked to C-16 and C-18 based on the HMBC correlations from H_2_-16 to C-1″ and H_2_-18 to C-1′, respectively. This assignment was corroborated by the downfield ^1^H and ^13^C NMR chemical shifts of H_2_-16a (*δ*_H_ 4.80), H_2_-16b (*δ*_H_ 4.66), and C-16 (*δ*_C_ 61.7) as well as H_2_-18 (*δ*_H_ 4.51) and C-18 (*δ*_C_ 64.5), respectively.

After determining the planar structure, the relative configuration of compound **1** was elucidated using a combination of ^1^H–^1^H ROESY correlations, diagnostic ^1^H–^1^H spin–spin coupling constants, ^1^H and ^13^C NMR chemical shift analysis, ^13^C NMR-based empirical rules, and comparison with data from the literature. The ^13^C NMR chemical shift difference in the resonance between C-17 and C-20 exceeding 10 ppm (∆*δ*_C-20–C-17_ = 11.2 ppm) suggested the *cis* relative configuration of the A/B ring junction and the *trans* relationship between the C-17 and C-20 methyl groups (characteristic of a *cis*–*trans* (CT)-type clerodane diterpene skeleton) [28,29]. The *cis*-A/B ring fusion was further proved by the downfield ^13^C NMR chemical shift of C-20 (*δ*_C_ 27.0); in *trans*-clerodanes, C-20 typically resonates between *δ*_C_ 17–19, whereas in *cis*-clerodanes, this resonance appears between *δ*_C_ 21–29 [30]. Furthermore, a downfield ^1^H NMR chemical shift of H_3_-20 (*δ*_H_ 0.92) relative to H_3_-17 (*δ*_H_ 0.79) also supports the presence of a *cis*-A/B ring junction [29]. The CT-type clerodane diterpene skeleton with a nonsteroidal conformation was confirmed by NOE enhancements between H_3_-17/H_3_-20, H_3_-17/H_2_-11b, H_3_-20/H_2_-1a, H_3_-20/H-8, and H_3_-20/H-10 and by the axial orientation of H-10 [28], which was revealed by its coupling constants of 12.9 Hz (axial–axial coupling with H_2_-1b) and 1.5 Hz (axial–equatorial coupling with H_2_-1a). The NOE interactions between H-3′/H_3_-5′ and H-3″/H_3_-5″ along with the absence of NOE correlations between H_3_-4′/H_3_-5′ and H_3_-4″/H_3_-5″ implied that the esterified group was an angelate ((2*Z*)-2-methylbut-2-enoate) with a *Z* double bond geometry. The presence of the angeloyloxy rather than the isomeric tigloyloxy moieties was confirmed by comparing the ^1^H NMR chemical shift of H-3′ (*δ*_H_ 6.04) and H-3″ (*δ*_H_ 6.10) with reference values from the literature: *δ*_H_ 6.06 for methyl angelate and *δ*_H_ 6.90 for methyl tiglate [31]. Finally, the ROESY spectrum featured cross-peaks between H_2_-12a/H-14, H_2_-12b/H-14, H_2_-11a/H-14, H_2_-11b/H-14, H_2_-15/H_2_-16a, and H_2_-15/H_2_-16b, although no correlation was observed between H-14/H_2_-16 and H_2_-12/H_2_-15, which is consistent with the *Z* configuration of the double bond C-13–C-14.

Solidagoic acids A and B were first isolated from the roots of *S. gigantea* [32], while solidagoic acids C–I were first reported from natural sources from the aerial parts of *S. virgaurea* [33]. Based on the structural similarity of compound **1** to solidagoic acids A–I, this previously undescribed *cis*-clerodane diterpenoid is herein assigned the trivial name solidagoic acid J, and its absolute configuration was given by analogy with solidagoic acids A–I. It should be noted that the specific rotation of compound **1** could not be reliably determined, owing to the limited sample quantity.

**Figure 3:**
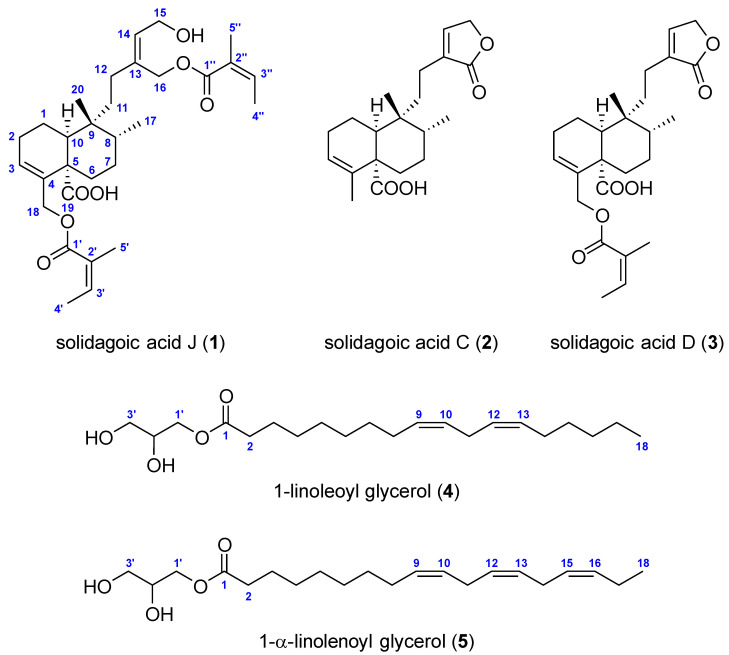
The chemical structures of the isolated compounds (**1**–**5**) with atomic numbering (blue).

**Figure 4:**
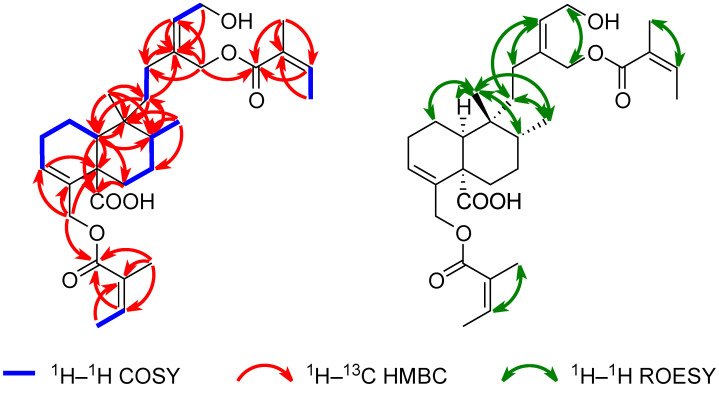
Key ^1^H–^1^H COSY (blue), ^1^H–^13^C HMBC (red), and ^1^H–^1^H ROESY (green) correlations of solidagoic acid J (**1**).

In addition, four known compounds, **2**–**5** (Figure 3), were identified based on their NMR and HRMS(/MS) data. Comparison of their spectroscopic and spectrometric data with values from the literature confirmed them as two *cis*-clerodane diterpenoids, solidagoic acid C (**2**) [33] and solidagoic acid D (**3**) [33], as well as two unsaturated monoacylglycerols, 1-linoleoyl glycerol (2,3-dihydroxypropyl (9*Z*,12*Z*)-9,12-octadecadienoate, 1-monolinolein) (**4**) [34,35] and 1-α-linolenoyl glycerol (2,3-dihydroxypropyl (9*Z*,12*Z*,15*Z*)-9,12,15-octadecatrienoate, 1-monolinolenin) (**5**) [34]. Due to the insufficient sample quantities and the low specific rotations [34,36,37], the enantiopurity and the absolute configuration of the C-2 chirality centre in compounds **4** and **5** could not be reliably determined.

Clerodane diterpenoids are a class of naturally occurring specialized metabolites known for their diverse biological and pharmacological activities, including antibacterial, antifungal, antitumor, insect antifeedant, anti-inflammatory, antiplasmodial, antiulcer, and cytotoxic effects [38]. Solidagoic acid J (**1**) is an undescribed *cis*-clerodane diterpenoid that has not yet been synthesized. Solidagoic acid C (**2**) was first prepared by Anthonsen et al. [32] and later isolated from the aerial parts of *S. virgaurea* [33]. It was also detected in the ethanolic leaf extract of *E. graminifolia* [39]. Solidagoic acid D (**3**) was similarly isolated from the aerial parts of *S. virgaurea* [33]. Solidagoic acids E and F, previously isolated by us from the leaves of *S. gigantea* [19], are the 15-hydroxy derivatives of solidagoic acids C (**2**) and D (**3**), respectively, indicating that the present study focused on slightly less polar compounds.

Monoacylglycerols, also known as monoglycerides, are low-abundance, transient glycerolipids that play a crucial role as intermediates in the lipid metabolism with antibacterial [40], antifungal [40], antiprotozoal [41], and antiviral [42] activities. The monoacylglycerols 1-linoleoyl glycerol (**4**) and 1-α-linolenoyl glycerol (**5**) were detected, identified, and isolated from various plant species [34,43,44,45,46] though not from the *Solidago* genus. Thus, compounds **2**–**5** were identified and isolated for the first time from *S. gigantea*, with compounds **4** and **5** also being first reported here from the *Solidago* genus.

### 2.3. Antimicrobial Assays

The antimicrobial potential of the isolated compounds (**1**–**5**) was evaluated against Gram-positive, non-pathogenic *B. subtilis* and a series of bacterial and fungal phytopathogens that were selected based on their relevance as common strains frequently associated with various plant diseases. Namely, the following pathogens were involved in this study: the Gram-positive pathogens *C. flaccumfaciens* pv. *flaccumfaciens*, isolated from bean; *C. michiganensis* (formerly *C. michiganensis* subsp. *michiganensis*), the causal agent of bacterial canker on tomato and *R. fascians*, isolated from chrysanthemum; the Gram-negative pathogens *P. syringae* pv. *tomato*, which infects tomato, and *Arabidopsis thaliana*; *X. arboricola* pv. *pruni*, which causes bacterial leaf and fruit spot on stone fruits; and the crop-pathogenic fungal strains *B. sorokiniana* and *F. graminearum*. The antibacterial, bactericidal, and antifungal activities of the isolates were compared to those of the respective positive controls, gentamicin (for bacteria) and benomyl (for fungi). The minimal inhibitory concentration (MIC) and minimal bactericidal concentration (MBC) values are depicted in Table 2.

As described in Table 2, compound **1** exhibited weak to moderate antibacterial activity against *B. subtilis*, *C. flaccumfaciens* pv. *flaccumfaciens*, and *C. michiganensis* with MIC values ranging from 67 to 133 μg/mL. For compound **1,** MIC and MBC values against certain bacterial strains could not be determined due to insufficient sample quantity. Compounds **2** and **4** showed a weak to moderate antibacterial effect only against *C. michiganensis* with MIC values of 133 and 33 μg/mL, respectively. Compounds **3** and **5** demonstrated weak to moderate antibacterial activity against *C. michiganensis* (MIC 33 and 17 μg/mL, respectively) and *R. fascians* (MIC 133 and 33 μg/mL, respectively). A weak antibacterial effect was also observed against *B. subtilis* for compound **3** with a MIC value of 133 μg/mL. Notably, only compound **1** was active against *C. flaccumfaciens* pv. *flaccumfaciens*, also inhibiting *C. michiganensis* with MIC values of 67 μg/mL However, the antibacterial activity of the positive control (antibiotic gentamicin) surpassed that of the tested compounds by 1–2 orders of magnitude. None of the isolates inhibited the bacterial growth of the Gram-negative *P. syringae* pv. *tomato* and *X. arboricola* pv. *pruni*. This fact may be attributed to the differences in the structural and functional properties of Gram-negative bacterial cell walls compared to those of Gram-positive bacteria, which often act as a barrier to many antibacterial agents [47]. Compounds **2** and **3** displayed a moderate antifungal effect against *B. sorokiniana* with 45% and 56% and against *F. graminearum* with 56% and 51% mycelium growth inhibition at the highest concentration (333 μg/mL), respectively. None of the isolated compounds demonstrated bactericidal activity at the tested concentrations, suggesting a bacteriostatic mode of action. Regarding structure–activity relationships, an angelate group in clerodane diterpenoids (compound **3** compared to **2**) was shown to enhance the antibacterial activity. Similarly, the presence of an additional double bond improved the antibacterial effect of monoglycerides (compound **5** compared to compound **4**).

Compounds **2** and **3** demonstrated moderate antibacterial activity against *Staphylococcus aureus* with MIC values of 60 and 30 µg/mL, respectively [33]. Compound **4** displayed antifungal inhibitory rates exceeding 50% against *Trichophyton mentagrophytes* [48]. Additionally, its antimicrobial effect was reported in an agar diffusion assay against a range of fungi, *Emericellopsis minima*, *Microascus* sp., *Monochaetia* sp., *Phoma* sp., and *Scopulariopsis candida*, all of them isolated from marine sponges [49]. Notably, 1-linoleoyl-(2*R*) glycerol was found to be more potent than its (*S*) enantiomer against *B. subtilis* (MIC 200 µg/mL vs. >200 µg/mL) and *S. aureus* (MIC 100 µg/mL vs. 200 µg/mL) [36]. However, it exhibited no antibacterial activity against *Erysipelothrix rhusiopathiae* (MIC > 100 µg/mL), *Escherichia coli* (MIC > 100 µg/mL), *Streptococcus suis* (MIC > 100 µg/mL), and *Pseudomonas aeruginosa* [50] and no antifungal effect (MIC > 100 µg/mL) was observed against the fungi *Botrytis cinerea*, *Verticillium dahliae*, *F. graminearum*, *Fusarium oxysporum*, and *Rhizoctonia solani* [50]. Our results are in agreement with data from the literature (MIC > 133 µg/mL against both *B. subtilis* and *F. graminearum*). The MIC value of compound **5** against *B. subtilis* was reported to be 400 µg/mL [51], which is consistent with the lack of inhibition at 133 µg/mL in the present study.

## 3. Materials and Methods

### 3.1. Materials

Glass- and aluminum-backed HPTLC and TLC silica gel 60 F_254_ plates (all 20 × 10 cm) were purchased from Merck (Darmstadt, Germany). Xprep preparative silica gel (pore size: 6.65 nm, particle size: 230–400 mesh) was supplied by LAB-EX (Budapest, Hungary). All chemicals were used as received from commercial suppliers without further purification. Solvents of analytical grade (acetone, *tert*-butyl alcohol, chloroform (stabilized with 5–50 ppm amylene), ethyl acetate, *n*-hexane, isopropyl alcohol, methanol) and gradient-grade methanol were obtained from Molar Chemicals (Halásztelek, Hungary). LC-MS-grade methanol was purchased from VWR (Radnor, PE, USA), while LC-MS-grade water was from Reanal (Budapest, Hungary). Bidistilled water was prepared by a Vitrotech VDB-3A apparatus (Vitro-Tech-Lab Ltd., Gyál, Hungary). Ultrapure water was generated by a Millipore Direct-Q 3 UV Water Purification System (Merck). Benomyl, formic acid (LC-MS grade), gentamicin, and *p*-anisaldehyde were from Sigma-Aldrich (Burlington, MA, USA). 3-(4,5-Dimethylthiazol-2-yl)-2,5-diphenyltetrazolium bromide (MTT) was acquired from Carl Roth (Karlsruhe, Germany), concentrated sulfuric acid (96%) from Carlo Erba (Milan, Italy), and acetic acid from Lach-Ner (Neratovice, Czech Republic). For NMR spectroscopy, chloroform-*d* (99.8 atom% D) was purchased from Eurisotop (Saint-Aubin, France) or Merck. Tryptone (from casein, pancreatic digest) and sodium chloride were obtained from Reanal, agar from Merck, and yeast extract from Scharlab (Barcelona, Spain). Nutrient Broth (NB) was from Biolab (Budapest, Hungary). The Gram-positive soil bacterium *Bacillus subtilis* (F1276) was a gift from József Farkas (Central Food Research Institute, Budapest, Hungary). *Curtobacterium flaccumfaciens* pv. *flaccumfaciens* (NCAIM B.01609) and *Clavibacter michiganensis* (NCAIM B.01813) were received from Dénes Dlauchy (National Collection of Agricultural and Industrial Microorganisms, Budapest, Hungary). *Fusarium graminearum* Schwabe (NCAIM F.00730) and *Rhodococcus fascians* (NCAIM B.01608) were purchased from the National Collection of Agricultural and Industrial Microorganisms (NCAIM, Budapest, Hungary). The *Xanthomonas arboricola* pv. *pruni* strain was isolated from *Prunus armeniaca* L. cv. Bergecot in 2016 (No. XapHU1, I. Schwarczinger, Plant Protection Institute, HUN-REN Centre for Agricultural Research, Budapest, Hungary). *Bipolaris sorokiniana* (Sacc.) Shoemaker H-299 (NCBI GenBank accession No. MH697869) was collected from barley in Hungary. *Pseudomonas syringae* pv. *tomato* DC3000 Lux was donated by Julia Vorholt (ETH Zurich, Zurich, Switzerland).

### 3.2. Plant Material

The leaves of *S. gigantea* were collected in July 2020 near Harta, Hungary (46° 41′ 51.5″ N 19° 02′ 52.4″ E, altitude: 90 m a. s. l.). A voucher specimen (Figure 1c) has been deposited at the Hungarian Natural History Museum, Budapest, Hungary, under the accession number HNHM-TRA 00027284. The fresh plant material was dried at room temperature and powdered with a coffee grinder (Sencor SCG 2050, Říčany, Czech Republic).

### 3.3. (High-Performance) TLC–UV/FLD Method

Samples were applied manually by a 10 µL microsyringe (Hamilton, Bonaduz, Switzerland) as 5 mm bands with 6–10 mm track distance and 8 mm distance from the lower edge on high-performance (HP)TLC silica gel 60 F_254_ plates (Merck). (HP)TLC separation was carried out in a pre-saturated (for 10 min) twin-trough chamber (CAMAG, Muttenz, Switzerland) with the mobile phase chloroform–ethyl acetate–methanol, 15:3:2 (*V*/*V*) up to 70 mm from the lower plate edge. After development, the plates were dried in a cold air stream of a hair dryer and documented with a digital camera (Cybershot DSC-HX60, Sony, Neu-Isenberg, Germany) under a UV lamp (CAMAG) at 254 nm and 366 nm.

#### 3.3.1. Detection via *p*-Anisaldehyde Sulfuric Acid Reagent

For the derivatization, the developed (HP)TLC chromatoplates were dipped into the *p*-anisaldehyde sulfuric acid reagent (500 μL of *p*-anisaldehyde, 10 mL of acetic acid, 100 mL of methanol, and 5 mL of concentrated sulfuric acid (96%)), followed by heating at 110 °C for 5 min (Advanced Hot Plate, VWR) and detecting at Vis under white light illumination in the transmittance mode (96891 Salobrena 2 LED lamp, EGLO Lux, Dunakeszi, Hungary).

#### 3.3.2. Detection via Planar *B. subtilis* Antibacterial Assay

The TLC–*B. subtilis* bioassay was performed according to the protocol previously described [52]. Briefly, the developed and dried TLC plates were immersed in the bacterial cell suspension (OD_600_ = 1.2) and then incubated for 2 h in a vapor chamber (at 37 °C and 100% relative humidity). Subsequently, the plates were immersed in an aqueous MTT solution (1 mg/mL) and incubated for an additional 15 min. The bioautograms were documented under visible light, where antibacterial compounds appeared as bright inhibition zones against a purplish background.

### 3.4. Extraction and Isolation

The air-dried, powdered leaves (100 g) of *S. gigantea* were extracted by maceration with *n*-hexane (3 × 700 mL, each for 72 h) at room temperature. The filtered (Reanal filter paper; pore size: 7–10 μm) and combined *n*-hexane crude extract was concentrated at 40 °C under reduced pressure with a rotary evaporator (Rotavapor R-134, Büchi, Flawil, Switzerland) to afford a dry residue (9.8 g). It was subjected to manual normal-phase column chromatography using a silica gel column (30 g) with stepwise gradient elution (chloroform–acetone 19:1, *V*/*V*; *n*-hexane–acetone 1:1, *V*/*V*; acetone) to obtain fractions A-C. The solvent flow was accelerated by applying external pressure with an air compressor (HYD-24F, Hyundai, Seoul, Republic of Korea). Fraction B (3.65 g) was dried with a rotary evaporator, re-suspended in *n*-hexane, and subjected to normal-phase flash column chromatography (CombiFlash NextGen 300, Teledyne Isco, Lincoln, NE, USA) using a silica gel column (RediSep Rf Gold, 20–40 μm, 40 g) with a gradient solvent system of *n*-hexane and acetone (0–0.5 min, 0%; 0.5–20.5 min, 0–30%; 20.5–25.5 min, 30–50%; 25.5–27.5 min, 50–100% acetone; flow rate: 30 mL/min), yielding 67 fractions. Fractions B38-39 (354 mg) and B40 (256 mg) were separately purified by reversed-phase flash column chromatography on a C_18_ column (RediSep Rf Gold C18, 20–40 μm, 30 g) using a gradient solvent system of water with 0.1% formic acid and methanol (0.0–0.6 min, 0%; 0.6–1.2 min, 0–65%; 1.2–34.4 min, 65–100% methanol; flow rate: 20 mL/min) to furnish 53-53 subfractions, respectively. Subfraction B38-39/24-26 (20.5 mg) was further separated by normal-phase flash column chromatography using a silica gel column (RediSep Rf Gold, 20–40 μm, 24 g) with an isocratic elution of *n*-hexane–*tert*-butyl alcohol 23:2, *V/V* (flow rate: 20 mL/min) to obtain compound **2** (subfraction B38-39/24-26/18-24, *t*_R_ = 11.7–16.5 min, 10.7 mg). Subfraction B38-39/38-40 (4.9 mg) was purified by normal-phase flash column chromatography using a silica gel column (RediSep Rf Gold, 20–40 μm, 12 g) with a gradient solvent system of *n*-hexane and isopropyl alcohol (0.0–0.5 min, 0%; 0.5–20.5 min, 0–10%; 20.5–22.5 min, 10–100% isopropyl alcohol; flow rate: 20 mL/min) to give compound **1** (subfraction B38-39/38-40/17, *t*_R_ = 9.5–10.5 min, 0.8 mg) and compound **4** (subfraction B38-39/38-40/22-23, *t*_R_ = 14.3–16.2 min, 2.4 mg). Subfractions B40/30-32 (10.5 mg) and B40/36-38 (2.3 mg) were separately subjected to normal-phase flash column chromatography using a silica gel column (RediSep Rf Gold, 20–40 μm, 12 g) with an isocratic elution of *n*-hexane–acetone 4:1, *V/V* (flow rate: 10 mL/min) to obtain compound **3** (subfraction B40/30-32/15-30; *t*_R_ = 18.5–27.8 min; 9.3 mg) and compound **5** (subfraction B40/36-38/11-12, *t*_R_ = 14.9–18.7 min, 2.1 mg), respectively.

### 3.5. Compound Characterization

Solidagoic acid J (**1**): White amorphous solid; UV (EtOH) *λ*_max_ (log *ε*) 205 nm (3.81), 209 nm (3.80); IR (ATR) *ν*_max_ 3429, 2958, 2926, 2856, 1715, 1648, 1546, 1457, 1439, 1381, 1354, 1260, 1231, 1190, 1155, 1085, 1043, 1027, 971, 911 cm^−1^; ^1^H (500 MHz, CDCl_3_) and ^13^C (126 MHz, CDCl_3_) NMR spectroscopic data, see Table 1; HR-ESI-MS *m*/*z* 539.2980 [M+Na]^+^ (calculated for C_30_H_44_O_7_Na^+^, *m*/*z* 539.2979 [M+Na]^+^, error: 0.1 ppm), *m*/*z* 515.3013 [M−H]^−^ (calculated for C_30_H_43_O_7_^−^, *m*/*z* 515.3014 [M−H]^−^, error: −0.3 ppm); TLC: *R*_F_ 0.81 (chloroform–ethyl acetate–methanol 15:3:2, *V*/*V*).

Solidagoic acid C (**2**): White amorphous solid; ^1^H NMR (500 MHz, CDCl_3_) *δ* 7.16 (p, *J* = 1.2 Hz, 1H, H-14), 5.53 (t, *J* = 4.0 Hz, 1H, H-3), 4.76 (m, 2H, H_2_-15), 2.53 (td, *J* = 13.8, 1.7 Hz, 1H, H_2_-12b), 2.35 (ov., 1H, H-10), 2.32 (ov., 1H, H_2_-6a), 2.17 (td, *J* = 13.3, 4.7 Hz, 1H, H_2_-12a), 2.10 (m, 2H, H_2_-2), 1.73 (m, 1H, H_2_-1a), 1.70 (ov., 1H, H_2_-11a), 1.65 (ov., 1H, H-8), 1.65 (ov., 1H, H_2_-7a), 1.58 (q, *J* = 1.8 Hz, 3H, H_3_-18), 1.53 (m, 1H, H_2_-1b), 1.42 (m, 1H, H_2_-6b), 1.34 (m, 1H, H_2_-7b), 1.20 (td, *J* = 13.7, 5.0 Hz, 1H, H_2_-11b), 0.98 (s, 3H, H_3_-20), 0.80 (d, *J* = 5.9 Hz, 3H, H_3_-17); ^13^C NMR (126 MHz, CDCl_3_) *δ* 179.6 (C, C-19), 175.8 (C, C-16), 146.1 (CH, C-14), 136.0 (C, C-4), 135.4 (C, C-13), 123.6 (CH, C-3), 70.5 (CH_2_, C-15), 51.0 (C, C-5), 42.6 (CH, C-10), 38.9 (C, C-9), 37.1 (CH, C-8), 31.3 (CH_2_, C-11), 29.3 (CH_2_, C-6), 28.0 (CH_2_, C-7), 26.9 (CH_3_, C-20), 26.6 (CH_2_, C-2), 20.1 (CH_2_, C-12), 19.7 (CH_2_, C-1), 19.1 (CH_3_, C-18), 15.9 (CH_3_, C-17); HR-ESI-MS *m*/*z* 355.1879 [M+Na]^+^ (calculated for C_20_H_28_O_4_Na^+^, *m*/*z* 355.1880 [M+Na]^+^, error: −0.1 ppm), *m*/*z* 331.1915 [M−H]^−^ (calculated for C_20_H_27_O_4_^−^, *m*/*z* 331.1915 [M−H]^−^, error: −0.1 ppm); TLC: *R*_F_ 0.73 (chloroform–ethyl acetate–methanol 15:3:2, *V*/*V*).

Solidagoic acid D (**3**): White amorphous solid; ^1^H NMR (500 MHz, CDCl_3_) *δ* 7.18 (br s, 1H, H-14), 6.04 (qq, *J* = 7.3, 1.6 Hz, 1H, H-3′), 5.93 (t, *J* = 4.1 Hz, 1H, H-3), 4.78 (q, *J* = 1.4 Hz, 2H, H_2_-15), 4.53 (m, 2H, H_2_-18), 2.53 (br t, *J* = 13.3 Hz, 1H, H_2_-12a), 2.42 (ov., 1H, H_2_-6a), 2.40 (ov., 1H, H-10), 2.20 (m, 2H, H_2_-2), 2.17 (ov., 1H, H_2_-12b), 1.98 (dq, *J* = 7.3, 1.6 Hz, 3H, H_3_-4′), 1.89 (p, *J* = 1.6 Hz, 3H, H_3_-5′), 1.77 (ov., 1H, H_2_-1a), 1.76 (ov., 1H, H_2_-11a), 1.67 (ov., 1H, H_2_-7a), 1.66 (ov., 1H, H-8), 1.57 (ov., 1H, H_2_-1b), 1.53 (ov., 1H, H_2_-6b), 1.33 (ov., 1H, H_2_-7b), 1.16 (ov., 1H, H_2_-11b), 0.99 (s, 3H, H_3_-20), 0.80 (d, *J* = 6.1 Hz, 3H, H_3_-17); ^13^C NMR (126 MHz, CDCl_3_) *δ* 178.3* (C, C-19), 175.9 (C, C-16), 167.7 (C, C-1′), 146.4 (CH, C-14), 138.3 (CH, C-3′), 135.8* (C, C-4), 135.5 (C, C-13), 128.2* (C, C-2′), 128.0 (CH, C-3), 70.6 (CH_2_, C-15), 64.4 (CH_2_, C-18), 50.0 (C, C-5), 42.7 (CH, C-10), 39.0 (C, C-9), 37.0 (CH, C-8), 31.5 (CH_2_, C-11), 30.0 (CH_2_, C-6), 28.0 (CH_2_, C-7), 27.1 (CH_3_, C-20), 26.6 (CH_2_, C-2), 20.7 (CH_3_, C-5′), 20.1 (CH_2_, C-12), 19.7 (CH_2_, C-1), 15.9 (CH_3_, C-4′), 15.8 (CH_3_, C-17); HR-ESI-MS *m*/*z* 453.2247 [M+Na]^+^ (calculated for C_25_H_34_O_6_Na^+^, *m*/*z* 453.2248 [M+Na]^+^, error: 0.1 ppm), *m*/*z* 429.2280 [M−H]^−^ (calculated for C_25_H_33_O_6_^−^, *m*/*z* 429.2283 [M−H]^−^, error: −0.5 ppm); TLC: *R*_F_ 0.71 (chloroform–ethyl acetate–methanol 15:3:2, *V*/*V*).

1-Linoleoyl glycerol (**4**): Yellow oil; ^1^H NMR (500 MHz, CDCl_3_) *δ* 5.34 (m, 4H, H-9, H-10, H-12, H-13), 4.21 (dd, *J* = 11.4, 4.8 Hz, 1H, H_2_-1′a), 4.15 (dd, *J* = 11.4, 6.0 Hz, 1H, H_2_-1′b), 3.93 (m, 1H, H-2′), 3.70 (dd, *J* = 11.4, 3.6 Hz, 1H, H_2_-3′a), 3.60 (dd, *J* = 11.4, 6.0 Hz, 1H, H_2_-3′b), 2.77 (t, *J* = 6.9 Hz, 2H, H_2_-11), 2.34 (t, *J* = 7.7 Hz, 2H, H_2_-2), 2.03 (m, 4H, H_2_-8, H_2_-14), 1.63 (m, 2H, H_2_-3), 1.31 (m, 14H, H_2_-4, H_2_-5, H_2_-6, H_2_-7, H_2_-15, H_2_-16, H_2_-17), 0.87 (m, 3H, H_3_-18); ^13^C NMR (126 MHz, CDCl_3_) *δ* 174.4* (C, C-1), 130.4/130.2 (CH, C-9/C-13), 128.3/128.1 (CH, C-10/C-12), 70.5 (CH, C-2′), 65.4 (CH_2_, C-1′), 63.5 (CH_2_, C-3′), 34.3 (CH_2_, C-2), 31.7 (CH_2_, C-16), 29.8/29.7/29.5/29.3/29.2 (CH_2_, C-4/C-5/C-6/C-7/C-15), 27.4/27.3 (CH_2_, C-8/C-14), 25.8 (CH_2_, C-11), 25.1 (CH_2_, C-3), 22.7 (CH_2_, C-17), 14.3 (CH_3_, C-18); HR-ESI-MS *m*/*z* 377.2662 [M+Na]^+^ (calculated for C_21_H_38_O_4_Na^+^, *m*/*z* 377.2662 [M+Na]^+^, error: 0.0 ppm); *m*/*z* 355.2843 [M+H]^+^ (calculated for C_21_H_39_O_4_^+^, *m*/*z* 355.2843 [M+H]^+^, error: 0.0 ppm); TLC: *R*_F_ 0.59 (chloroform–ethyl acetate–methanol 15:3:2, *V*/*V*).

1-α-Linolenoyl glycerol (**5**): Yellow oil; ^1^H NMR (500 MHz, CDCl_3_) *δ* 5.36 (m, 6H, H-9, H-10, H-12, H-13, H-15, H-16), 4.21 (dd, *J* = 11.6, 4.3 Hz, 1H, H_2_-1′a), 4.15 (dd, *J* = 11.6, 6.0 Hz, 1H, H_2_-1′b), 3.93 (m, 1H, H-2′), 3.70 (dd, *J* = 11.5, 3.9 Hz, 1H, H_2_-3′a), 3.60 (dd, *J* = 11.5, 5.7 Hz, 1H, H_2_-3′b), 2.81 (t, *J* = 6.3 Hz, 4H, H_2_-11, H_2_-14), 2.35 (t, *J* = 7.6 Hz, 2H, H_2_-2), 2.07 (m, 4H, H_2_-8, H_2_-17), 1.63 (m, 2H, H_2_-3), 1.32 (m, 8H, H_2_-4–H_2_-7), 0.98 (t, *J* = 7.5 Hz, 3H, H_3_-18); ^13^C NMR (126 MHz, CDCl_3_) *δ* 174.4* (C, C-1), 132.1 (CH, C-16), 130.4 (CH, C-9/C-10), 128.5/128.4 (CH, C-12/C-13), 127.9 (CH, C-9/C-10), 127.3 (CH, C-15), 70.5 (CH, C-2′), 65.4 (CH_2_, C-1′), 63.5 (CH_2_, C-3′), 34.3 (CH_2_, C-2), 29.9/29.7/29.3/29.2 (CH_2_, C-4/C-5/C-6/C-7), 27.4 (CH_2_, C-8), 25.8/25.7 (CH_2_, C-11/C-14), 25.1 (CH_2_, C-3), 20.7 (CH_2_, C-17), 14.4 (CH_3_, C-18).; HR-ESI-MS *m*/*z* 375.2506 [M+Na]^+^ (calculated for C_21_H_36_O_4_Na^+^, *m*/*z* 375.2506 [M+Na]^+^, error: 0.0 ppm); *m*/*z* 353.2686 [M+H]^+^ (calculated for C_21_H_37_O_4_^+^, *m*/*z* 353.2686 [M+H]^+^, error: 0.0 ppm); TLC: *R*_F_ 0.58 (chloroform–ethyl acetate–methanol 15:3:2, *V*/*V*).

Chemical shifts marked with an asterisk sign (*) were determined from HMBC spectra. The multiplicity of overlapped (ov.) resonances is not reported.

### 3.6. TLC–ESI-MS

For TLC–MS, methanol was delivered at a flow rate of 0.2 mL/min by a binary HPLC pump (LC-20AB, Shimadzu, Kyoto, Japan) through the oval elution head (4 mm × 2 mm) of the TLC-MS Interface 2 (CAMAG) into a single quadrupole electrospray ionization mass spectrometer (LCMS-2020, Shimadzu). The mass spectrometric conditions were as follows: the nebulizer gas (N_2_) flow rate, 1.5 L/min; the drying gas (N_2_) flow rate, 10 L/min; the interface temperature, 350 °C; the heat block temperature, 400 °C, and the desolvation line temperature, 250 °C. The detector voltage was set to +4.5 kV in positive and −4.5 kV in negative ionization modes. Full scan mass spectra were recorded in positive and negative ionization modes in the *m*/*z* range of 200–950, with a scan speed of 790 amu/s. Instrument control, data acquisition, and data analysis were performed using the LabSolutions software version 5.42v (Shimadzu).

### 3.7. FIA–HR-HESI-MS(/MS)

HR-HESI-MS(/MS) spectra of the isolated compounds were acquired via flow injection analysis (FIA). A UHPLC (Vanquish Flex VF-P10, Dionex Softron, Germering, Germany) instrument was coupled to a hybrid quadrupole-orbitrap mass spectrometer equipped with an HESI-II probe (Orbitrap Exploris 120, Thermo Fisher Scientific, Bremen, Germany). The flow rate of methanol was 0.2 mL/min. Full scan mass spectra were obtained in both positive and negative ionization modes in the range of *m*/*z* 100–1000 with a resolution of 120,000. The spray voltage was set to 3.4 kV in positive mode and −2.0 kV in negative mode. The ion transfer tube temperature was maintained at 320 °C and the vaporizer temperature at 250 °C. Nitrogen, produced by a Peak Scientific gas generator (Genius XE 35, Glasgow, UK), was used as both sheath and auxiliary gas at flow rates of 10 and 5 arbitrary units, respectively.

Tandem mass (MS/MS) spectra were recorded in HCD fragmentation mode using normalized collision energies ranging from 20% to 60%. Precursor ions were selected with a quadrupole isolation window of *m*/*z* 0.4. MS/MS spectra were acquired with a resolution of 120,000 without lock mass correction. Instrument control and data processing were carried out using Xcalibur software version 4.7.69 (Thermo Fisher Scientific).

### 3.8. NMR Spectroscopy

Samples were dissolved in chloroform-*d* (CDCl_3_) and transferred to standard 5 mm NMR tubes for analysis. All NMR spectra were acquired on either a Varian Unity INOVA 500 (^1^H: 499.6 MHz, ^13^C: 125.7 MHz; 11.7 T) spectrometer equipped with an inverse-detection pulsed-field gradient (IDPFG) probe head or a Bruker AVANCE III 500 (^1^H: 500.1 MHz, ^13^C: 125.8 MHz; 11.7 T) spectrometer equipped with a 5 mm triple-resonance, *z*-gradient cryoprobe (CP TCI 500S2 H-C/N-D-05 Z) (Bruker Corporation, Billerica, MA, USA) at 303 or 296 K, respectively. The instruments were operated and controlled by VnmrJ 4.2 or Bruker TopSpin 3.5 software. All standard pulse sequences were taken from the spectrometer software libraries (VnmrJ 4.2 or TopSpin 3.5). ^1^H and ^13^C chemical shifts are reported on the delta (*δ*) scale as parts per million (ppm) referenced to the NMR solvent used (CHCl_3_ residual peak at *δ*_H_ = 7.26 ppm and CDCl_3_ at *δ*_C_ = 77.16 ppm). Spin–spin coupling constant (*J*) values are reported in hertz (Hz). Signal multiplicities are denoted as follows: s—singlet; br s—broad singlet; d—doublet; t—triplet; br t—broad triplet; q—quartet; p—pentet; m—multiplet; dd—doublet of doublets; dt—doublet of triplets; td—triplet of doublets; dq—doublet of quartets; and qq—quartet of quartets. The complete ^1^H and ^13^C resonance assignments were carried out using conventional one-dimensional (1D) ^1^H, ^13^C, and ^13^C DEPTQ as well as two-dimensional (2D) homonuclear ^1^H–^1^H COSY, ^1^H–^1^H double-quantum-filtered (DQF)-COSY, ^1^H–^1^H TOCSY, ^1^H–^1^H ROESY, and heteronuclear ^1^H–^13^C multiplicity-edited HSQC (edHSQC) and ^1^H–^13^C HMBC (optimized for *^n^J*_C–H_ = 8 Hz) experiments. For compound **1**, 2D band-selective ^1^H–^13^C HSQC and HMBC spectra were also acquired to enhance the spectral resolution in the F1 dimension.

### 3.9. Polarimetry, UV, and ATR-FTIR Spectroscopy

#### 3.9.1. Polarimetry

Optical rotations were recorded at 25 °C at the sodium D-line (589.3 nm) using a PerkinElmer 341 LC polarimeter (PerkinElmer, Waltham, MA, USA) with an optical path length of 1 dm. Chloroform was used as a solvent.

#### 3.9.2. UV Spectroscopy

UV spectra were obtained using a PerkinElmer Lambda 35 spectrophotometer at room temperature. The spectra were acquired in the range of 190–400 nm with a scan speed of 60 nm/min and a slit width of 1 nm. Data processing and analysis were performed by the UV WinLab 5.2.0.0646 software.

#### 3.9.3. ATR-FTIR Spectroscopy

ATR-FTIR spectra were recorded using a PerkinElmer Spectrum 400 FT-IR/FT-NIR spectrometer equipped with a diamond/ZnSe ATR crystal and a MIR TGS detector. The spectra were collected in the range of 4000–650 cm^−1^ with a spectral resolution of 4 cm^−1^, averaging 32 scans. Data were processed and analyzed by PerkinElmer Spectrum Software version 6.3.1.

### 3.10. In Vitro Antimicrobial Activity Assays

#### 3.10.1. Determination of Minimal Inhibitory Concentration (MIC) Values

*B. subtilis* was cultured in Luria–Bertani (LB) broth (10 g/L tryptone, 5 g/L yeast extract, 10 g/L sodium chloride) at 37 °C for 24 h, *P. syringae* pv. *tomato* in LB broth at 28 °C for 24 h, *C. flaccumfaciens* pv. *flaccumfaciens*, *C. michiganensis*, and *X. arboricola* pv. *pruni* in Nutrient Broth (11 g/L peptones, 5 g/L sodium chloride) at 28 °C for 24 h, and *R. fascians* in Waksman broth (5 g/L peptone, 5 g/L meat extract, 5 g/L sodium chloride, 10 g/L glucose, pH adjusted to 7.2 with a 40% aqueous sodium hydroxide solution) at 30 °C for 24 h. Prior to the antibacterial assays, each bacterial suspension was diluted with the broth specified above to reach 10^5^ CFU/mL. *F. graminearum* and *B. sorokiniana* were grown in LB broth at 21 °C for 72 h by shaking at 120 rpm. Prior to the antifungal assays, mycelia were washed in fresh LB medium and fragmented using a FastPrep^®^-24 Classic homogenizer (MP Biomedicals, Irvine, CA, USA). The mycelia were suspended in 1 mL of the same medium and transferred into 2 mL Eppendorf tubes containing 7 glass beads (2 mm diameter). Homogenization was performed at an acceleration of 4.5 m/s for 2 × 20 s and finally a mycelium suspension of OD_600_ = 0.2 was prepared in LB broth.

The in vitro antimicrobial activities of the isolated compounds were evaluated based on their minimal inhibitory concentration (MIC) and minimal bactericidal concentration (MBC) values against the Gram-positive *B. subtilis*, *C. flaccumfaciens* pv. *flaccumfaciens*, *C. michiganensis*, and *R. fascians* and the Gram-negative *P. syringae* pv. *tomato*, and *X. arboricola* pv. *pruni* bacterial strains as well as against the *F. graminearum* and *B. sorokiniana* fungal strains using a microplate assay as previously described [24] with minor modifications. For the antibacterial assays, non-treated, flat-bottom 96-well microplates (VWR, cat #734-2781) were used, whereas for the antifungal assays, non-treated, U-bottom, 96-well microtiter plates (Nest Scientific, Woodbridge, NJ, USA, cat #701111) were utilized. A two-fold ethanolic dilution series of 10 µL of the isolates (for antibacterial assays, 2 mg/mL in ethanol for each compound, whereas for antifungal assays, 4 mg/mL in ethanol for compounds **2** and **3** as well as 2 mg/mL in ethanol for compounds **1**, **4**, and **5**) was prepared in the microplates in triplicate. Gentamicin (0.1 mg/mL in water) and benomyl (25 mg/mL in ethanol) were used as the positive controls against the bacteria and fungi, respectively, while ethanol was the negative control. After dilution, ethanol was evaporated from the wells in a sterile box. A total of 150 µL of bacterial suspension (10^5^ CFU/mL) for the antibacterial assay or 70 µL of LB broth and then 50 µL of a mycelium suspension (OD_600_ = 0.2 in LB broth) for the antifungal assay was added to each well. The absorbance was measured at 600 nm by a CLARIOstar^®^ Plus microplate reader (BMG LABTECH, Ortenberg, Germany) immediately and after 24 h (*B. subtilis* at 37 °C, *P. syringae* pv. *tomato*, and *X. arboricola* pv. *pruni* at 28 °C) or 48 h (*C. flaccumfaciens* pv. *flaccumfaciens* and *C. michiganensis* at 28 °C, *R.* fascians at 30 °C) incubation by shaking at 500 rpm with a Grant PHMP microplate thermoshaker (Grant Instruments, Royston, UK) or after 72 h incubation without shaking (*F. graminearum* and *B. sorokiniana* at 21 °C). The experiments were repeated on two separate occasions.

#### 3.10.2. Determination of Minimal Bactericidal Concentration (MBC) Values

An aliquot (10 µL) was taken from the microplate wells where no bacterial growth was observed after 24 h or 48 h incubation and was then dotted onto the surface of LB (*B. subtilis*) or NB (*C. flaccumfaciens* pv. *flaccumfaciens* and *C. michiganensis*) agar plates. The MBC was defined as the lowest concentration of the isolated compound at which no colonies formed after 24 h (*B. subtilis*) or 48 h (*C. flaccumfaciens* pv. *flaccumfaciens* and *C. michiganensis*) incubation at 37 °C or 28 °C, respectively.

## 4. Conclusions

In summary, a non-targeted, effect-directed phytochemical investigation of a *n*-hexane leaf extract of *S. gigantea* led to the bioassay-guided isolation, characterization, and structure elucidation of five compounds, including diangelate solidagoic acid J (**1**), a previously undescribed *cis*-clerodane diterpenoid, as well as two known *cis*-clerodane diterpenoids, solidagoic acid C (**2**) and solidagoic acid D (**3**), and two known monoglycerides, 1-linoleoyl glycerol (**4**) and 1-α-linolenoyl glycerol (**5**), which are reported here for the first time from this species. The evaluation of their antimicrobial activity against several plant pathogenic bacterial and fungal strains revealed that all isolated compounds exhibited weak to moderate antibacterial activity against *C. michiganensis*. Compound **1** was the most effective against *B. subtilis* and *C. flaccumfaciens* pv. *flaccumfaciens*, whereas compound **5** demonstrated the highest antibacterial activity against *C. michiganensis* and *R. fascians*. The moderate antifungal activity of compounds **2** and **3** was also observed against *B. sorokiniana* and *F. graminearum*.

The present study enriches the phytochemical and biological knowledge of *S. gigantea* and highlights the potential of thin-layer chromatography combined with direct bioautography and mass spectrometry as an effective tool for identifying novel bioactive compounds within a bioassay-guided isolation workflow. Furthermore, the isolated compounds show promising potential to reduce the ecological impact of synthetic pesticides and may serve as leads for the development of more effective, plant-derived pesticide candidates for sustainable plant protection.

## Figures and Tables

**Table 1:** ^1^H NMR (500 MHz) and ^13^C NMR (126 MHz) spectroscopic data of solidagoic acid J (**1**) in CDCl_3_ (*δ* in ppm, *J* in Hz). The multiplicity of overlapped (ov.) signals is not reported.

Position	Solidagoic Acid J (1)	
	*δ*_H_ (ppm), Multiplicity, *J* (Hz)	*δ*_C_ (ppm), Type
1a	1.75, m	19.6, CH_2_
1b	1.54, ov.	
2a	2.17, m	26.5, CH_2_
2b		
3	5.91, t (4.0)	128.1, CH
4	–	136.2, C
5	–	50.0, C
6a	2.41, dt (13.9, 2.8)	30.1, CH_2_
6b	1.51, ov.	
7a	1.68, ov.	28.0, CH_2_
7b	1.33, m	
8	1.65, ov.	37.0, CH
9	–	38.7, C
10	2.32, dd (12.9, 1.5)	42.6, CH
11a	1.59, ov.	30.0, CH_2_
11b	1.22, td (13.7, 4.8)	
12a	2.26, td (14.3, 2.4)	29.4, CH_2_
12b	1.94, td (13.6, 4.7)	
13	–	139.1, C
14	5.72, t (7.1)	128.7, CH
15	4.26, d (7.1)	58.7, CH_2_
16a	4.80, d (12.3)	61.7, CH_2_
16b	4.66, d (12.3)	
17	0.79, d (6.4)	15.8, CH_3_
18	4.51, m	64.5, CH_2_
19	–	178.8, C
20	0.92, s	27.0, CH_3_
1′	–	167.7, C
2′	–	128.0, C
3′	6.04, qq (7.2, 1.3)	138.3, CH
4′	1.97, ov.	15.9, CH_3_
5′	1.88, ov.	20.8, CH_3_
1″	–	168.7, C
2″	–	127.7, C
3″	6.10, qq (7.2, 1.3)	139.3, CH
4″	1.97, ov.	16.0, CH_3_
5″	1.86, ov.	20.7, CH_3_

ov.: overlapping peaks.

**Table 2:** The minimal inhibitory concentration (MIC) and minimal bactericidal concentration (MBC) values of the isolated compounds (**1**–**5**) and positive controls, gentamicin (Gent) and benomyl (Ben), against the *Bacillus subtilis* (*Bs*), *Curtobacterium flaccumfaciens* pv. *flaccumfaciens* (*Cff*), *Clavibacter michiganensis* (*Cm*), *Pseudomonas syringae* pv. *tomato* (*Pstom*), *Rhodococcus fascians* (*Rf*), and *Xanthomonas arboricola* pv. *pruni* (*Xap*) bacterial strains and against the *Bipolaris sorokiniana* (*Bip*) and *Fusarium graminearum* (*Fg*) fungal strains given in µg/mL. Gram-positive and Gram-negative bacteria are denoted as G+ and G−, respectively. For compound **1,** MIC and MBC values against certain bacterial strains could not be determined due to insufficient sample quantity.

Compounds	*Bs* (G+)		*Cff* (G+)		*Cm* (G+)		*Rf* (G+)		*Pstom* (G−)		*Xap* (G−)		*Bip*	*Fg*
	MIC	MBC	MIC	MBC	MIC	MBC	MIC	MBC	MIC	MBC	MIC	MBC	MIC	MIC
**1**	67	>133	133	>133	67	>133	N/A	N/A	N/A	N/A	N/A	N/A	N/A	N/A
**2**	>133	>133	>133	>133	133	>133	>133	>133	>133	>133	>133	>133	>333 ^b^	>333 ^c^
**3**	133	>133	>133	>133	33	>133	133	>133	>133	>133	>133	>133	>333 ^c^	>333 ^d^
**4**	>133	>133	>133	>133	33	>133	>133	>133	>133	>133	>133	>133	>167	>167
**5**	>133	>133	>133	>133	17	>133	33	>133	>133	>133	>133	>133	>167	>167
**Gent** ^a^	0.8	1.7	0.8	0.8	1.7	1.7	0.8	1.7	0.4	0.8	1.7	1.7		
**Ben** ^a^													1042	521

N/A—no data available; ^a^ positive control; ^b^ 45% inhibition at 333 µg/mL; ^c^ 56% inhibition at 333 µg/mL; ^d^ 51% inhibition at 333 µg/mL.

## Data Availability

The data supporting the findings of this study are available in the Supporting Information and from the corresponding author upon reasonable request.

## Supplementary Materials

The following supporting information can be downloaded at https://www.mdpi.com/article/10.3390/plants14142152/s1. Figures S1–S18: One-dimensional and two-dimensional NMR, HR-ESI-MS(/MS), UV, and ATR-FTIR spectra for compound **1**; Figures S19–S29: One-dimensional and two-dimensional NMR and HR-ESI-MS(/MS) spectra for compound **2**; Figures S30–S40: One-dimensional and two-dimensional NMR and HR-ESI-MS(/MS) spectra for compound **3**; Figures S41–S48: One-dimensional and two-dimensional NMR and HR-ESI-MS(/MS) spectra for compound **4**; Figures S49–S56: One-dimensional and two-dimensional NMR and HR-ESI-MS(/MS) spectra for compound **5**.
