# Supplementary material for: Bioassay-Guided Isolation of cis-Clerodane Diterpenoids and Monoglycerides from the Leaves of Solidago gigantea and Their Antimicrobial Activities
Source: Plants (Basel). 2025 Jul 11;14(14):2152. doi: 10.3390/plants14142152 (PMC12300610; doi:10.3390/plants14142152)
Supplement: Supplementary file 1 [file plants-14-02152-s001.zip › plants-3731490-supplementary.pdf]

Supplementary Materials

Table of contents

No.	Legend	Page
**Figure S1.**	^{1}H NMR spectrum of solidagoic acid J (**1**) ($CDCl_3$, 500 MHz).	S-4
**Figure S2.**	Partial ^{1}H NMR spectrum of solidagoic acid J (**1**) ($CDCl_3$, 500 MHz) – downfield region (δ_H 6.3–4.1).	S-4
**Figure S3.**	Partial ^{1}H NMR spectrum of solidagoic acid J (**1**) ($CDCl_3$, 500 MHz) – aliphatic region (δ_H 2.5–0.7).	S-5
**Figure S4.**	^{13}C DEPTQ NMR spectrum of solidagoic acid J (**1**) ($CDCl_3$, 126 MHz).	S-5
**Figure S5.**	^{1}H–^{1}H COSY NMR spectrum of solidagoic acid J (**1**) ($CDCl_3$, 500 MHz).	S-6
**Figure S6.**	^{1}H–^{13}C edHSQC NMR spectrum of solidagoic acid J (**1**) ($CDCl_3$, 500/126 MHz).	S-6
**Figure S7.**	^{1}H–^{13}C band-selective HSQC NMR spectrum of solidagoic acid J (**1**) ($CDCl_3$, 500/126 MHz) – aliphatic region (δ_C 31.0–15.0).	S-7
**Figure S8.**	^{1}H–^{13}C HMBC NMR spectrum of solidagoic acid J (**1**) ($CDCl_3$, 500/126 MHz).	S-7
**Figure S9.**	^{1}H–^{13}C band-selective HMBC NMR spectrum of solidagoic acid J (**1**) ($CDCl_3$, 500/126 MHz) – aliphatic region (δ_C 21.0–15.5).	S-8
**Figure S10.**	^{1}H–^{13}C band-selective HMBC NMR spectrum of solidagoic acid J (**1**) ($CDCl_3$, 500/126 MHz) – double bond region (δ_C 128.2–127.5).	S-8
**Figure S11.**	^{1}H–^{1}H TOCSY NMR spectrum of solidagoic acid J (**1**) ($CDCl_3$, 500 MHz).	S-9
**Figure S12.**	^{1}H–^{1}H ROESY NMR spectrum of solidagoic acid J (**1**) ($CDCl_3$, 500 MHz).	S-9
**Figure S13.**	HR-ESI^{+}-MS spectrum of solidagoic acid J (**1**).	S-10
**Figure S14.**	HR-ESI^{+}-MS/MS of solidagoic acid J (**1**) with a normalized HCD collision energy of 40%.	S-10
**Figure S15.**	HR-ESI^{-}-MS spectrum of solidagoic acid J (**1**).	S-10
**Figure S16.**	HR-ESI^{-}-MS/MS of solidagoic acid J (**1**) with a normalized HCD collision energy of 40%.	S-11
**Figure S17.**	UV spectrum of solidagoic acid J (**1**) in ethanol.	S-11
**Figure S18.**	ATR-FTIR spectrum of solidagoic acid J (**1**).	S-12
**Figure S19.**	^{1}H NMR spectrum of solidagoic acid C (**2**) ($CDCl_3$, 500 MHz).	S-12
**Figure S20.**	^{13}C NMR spectrum of solidagoic acid C (**2**) ($CDCl_3$, 126 MHz).	S-13
**Figure S21.**	^{1}H–^{1}H DQF-COSY NMR spectrum of solidagoic acid C (**2**) ($CDCl_3$, 500 MHz).	S-13
**Figure S22.**	^{1}H–^{13}C edHSQC NMR spectrum of solidagoic acid C (**2**) ($CDCl_3$, 500/126 MHz).	S-14
**Figure S23.**	^{1}H–^{13}C HMBC NMR spectrum of solidagoic acid C (**2**) ($CDCl_3$, 500/126 MHz).	S-14
**Figure S24.**	^{1}H–^{1}H TOCSY NMR spectrum of solidagoic acid C (**2**) ($CDCl_3$, 500 MHz).	S-15
**Figure S25.**	^{1}H–^{1}H ROESY NMR spectrum of solidagoic acid C (**2**) ($CDCl_3$, 500 MHz).	S-15
**Figure S26.**	HR-ESI^{+}-MS spectrum of solidagoic acid C (**2**).	S-16
**Figure S27.**	HR-ESI^{+}-MS/MS of solidagoic acid C (**2**) with a normalized HCD collision energy of 40%.	S-16
**Figure S28.**	HR-ESI^{-}-MS spectrum of solidagoic acid C (**2**).	S-16
**Figure S29.**	HR-ESI^{-}-MS/MS of solidagoic acid C (**2**) with a normalized HCD collision energy of 40%.	S-17
**Figure S30.**	^{1}H NMR spectrum of solidagoic acid D (**3**) ($CDCl_3$, 500 MHz).	S-17

**Figure S31.**	^{13}C NMR spectrum of solidagoic acid D (**3**) ($CDCl_3$, 126 MHz).	S-18
**Figure S32.**	^{1}H–^{1}H DQF-COSY NMR spectrum of solidagoic acid D (**3**) ($CDCl_3$, 500 MHz).	S-18
**Figure S33.**	^{1}H–^{13}C edHSQC NMR spectrum of solidagoic acid D (**3**) ($CDCl_3$, 500/126 MHz).	S-19
**Figure S34.**	^{1}H–^{13}C HMBC NMR spectrum of solidagoic acid D (**3**) ($CDCl_3$, 500/126 MHz).	S-19
**Figure S35.**	^{1}H–^{1}H TOCSY NMR spectrum of solidagoic acid D (**3**) ($CDCl_3$, 500 MHz).	S-20
**Figure S36.**	^{1}H–^{1}H ROESY NMR spectrum of solidagoic acid D (**3**) ($CDCl_3$, 500 MHz).	S-20
**Figure S37.**	HR-ESI^{+}-MS spectrum of solidagoic acid D (**3**).	S-21
**Figure S38.**	HR-ESI^{+}-MS/MS of solidagoic acid D (**3**) with a normalized HCD collision energy of 20%.	S-21
**Figure S39.**	HR-ESI^{-}-MS spectrum of solidagoic acid D (**3**).	S-21
**Figure S40.**	HR-ESI^{-}-MS/MS of solidagoic acid D (**3**) with a normalized HCD collision energy of 50%.	S-22
**Figure S41.**	^{1}H NMR spectrum of 1-linoleoyl glycerol (**4**) ($CDCl_3$, 500 MHz).	S-22
**Figure S42.**	^{13}C NMR spectrum of 1-linoleoyl glycerol (**4**) ($CDCl_3$, 126 MHz).	S-23
**Figure S43.**	^{1}H–^{1}H DQF-COSY NMR spectrum of 1-linoleoyl glycerol (**4**) ($CDCl_3$, 500 MHz).	S-23
**Figure S44.**	^{1}H–^{13}C edHSQC NMR spectrum of 1-linoleoyl glycerol (**4**) ($CDCl_3$, 500/126 MHz).	S-24
**Figure S45.**	^{1}H–^{13}C HMBC NMR spectrum of 1-linoleoyl glycerol (**4**) ($CDCl_3$, 500/126 MHz).	S-24
**Figure S46.**	^{1}H–^{1}H TOCSY NMR spectrum of 1-linoleoyl glycerol (**4**) ($CDCl_3$, 500 MHz).	S-25
**Figure S47.**	HR-ESI^{+}-MS spectrum of 1-linoleoyl glycerol (**4**).	S-25
**Figure S48.**	HR-ESI^{+}-MS/MS of 1-linoleoyl glycerol (**4**) with a normalized HCD collision energy of 50%.	S-26
**Figure S49.**	^{1}H NMR spectrum of 1-α-linolenoyl glycerol (**5**) ($CDCl_3$, 500 MHz).	S-26
**Figure S50.**	^{13}C NMR spectrum of 1-α-linolenoyl glycerol (**5**) ($CDCl_3$, 126 MHz).	S-27
**Figure S51.**	^{1}H–^{1}H DQF-COSY NMR spectrum of 1-α-linolenoyl glycerol (**5**) ($CDCl_3$, 500 MHz).	S-27
**Figure S52.**	^{1}H–^{13}C edHSQC NMR spectrum of 1-α-linolenoyl glycerol (**5**) ($CDCl_3$, 500/126 MHz).	S-28
**Figure S53.**	^{1}H–^{13}C HMBC NMR spectrum of 1-α-linolenoyl glycerol (**5**) ($CDCl_3$, 500/126 MHz).	S-28
**Figure S54.**	^{1}H–^{1}H TOCSY NMR spectrum of 1-α-linolenoyl glycerol (**5**) ($CDCl_3$, 500 MHz).	S-29
**Figure S55.**	HR-ESI^{+}-MS spectrum of 1-α-linolenoyl glycerol (**5**).	S-29
**Figure S56.**	HR-ESI^{+}-MS/MS of 1-α-linolenoyl glycerol (**5**) with a normalized HCD collision energy of 50%.	S-30

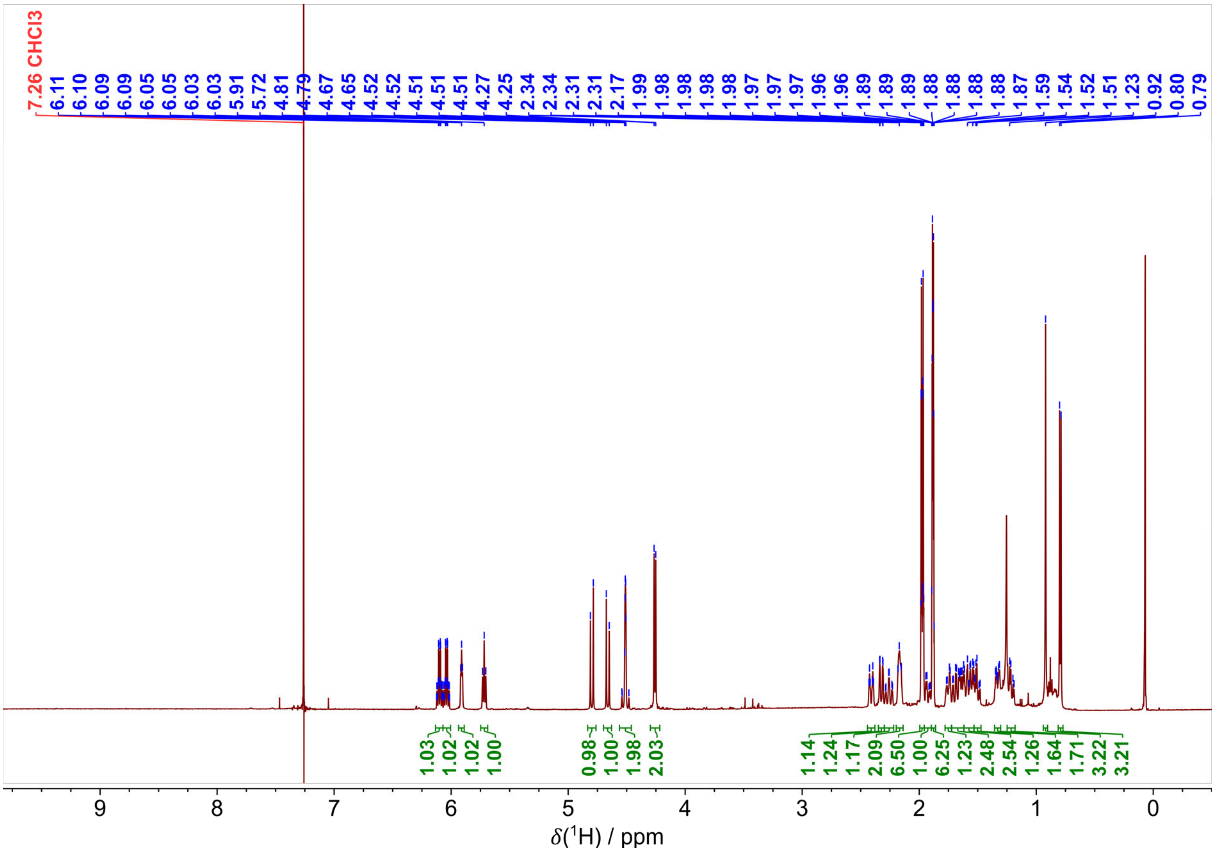

Figure S1. ^{1}H NMR spectrum of solidagoic acid J (**1**) ($CDCl_3$, 500 MHz).

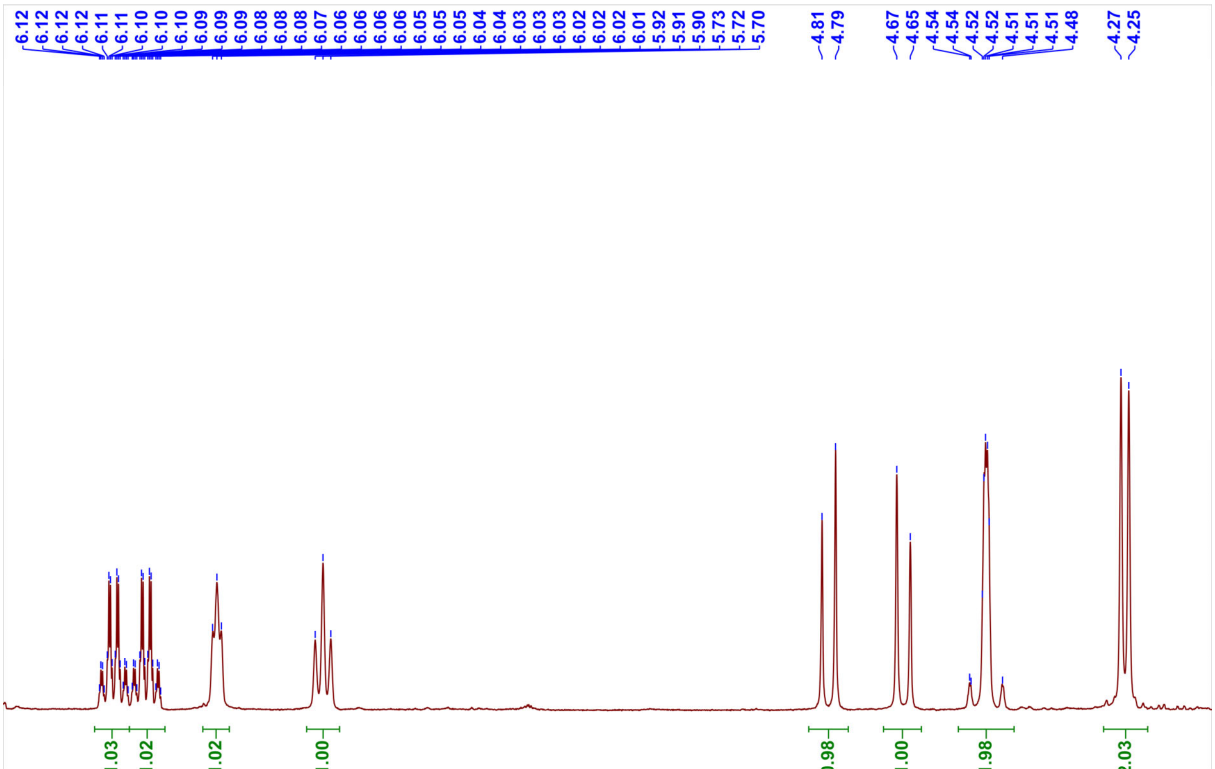

Figure S2. Partial ^{1}H NMR spectrum of solidagoic acid J (**1**) ($CDCl_3$, 500 MHz) – downfield region (δ_H 6.3–4.1).

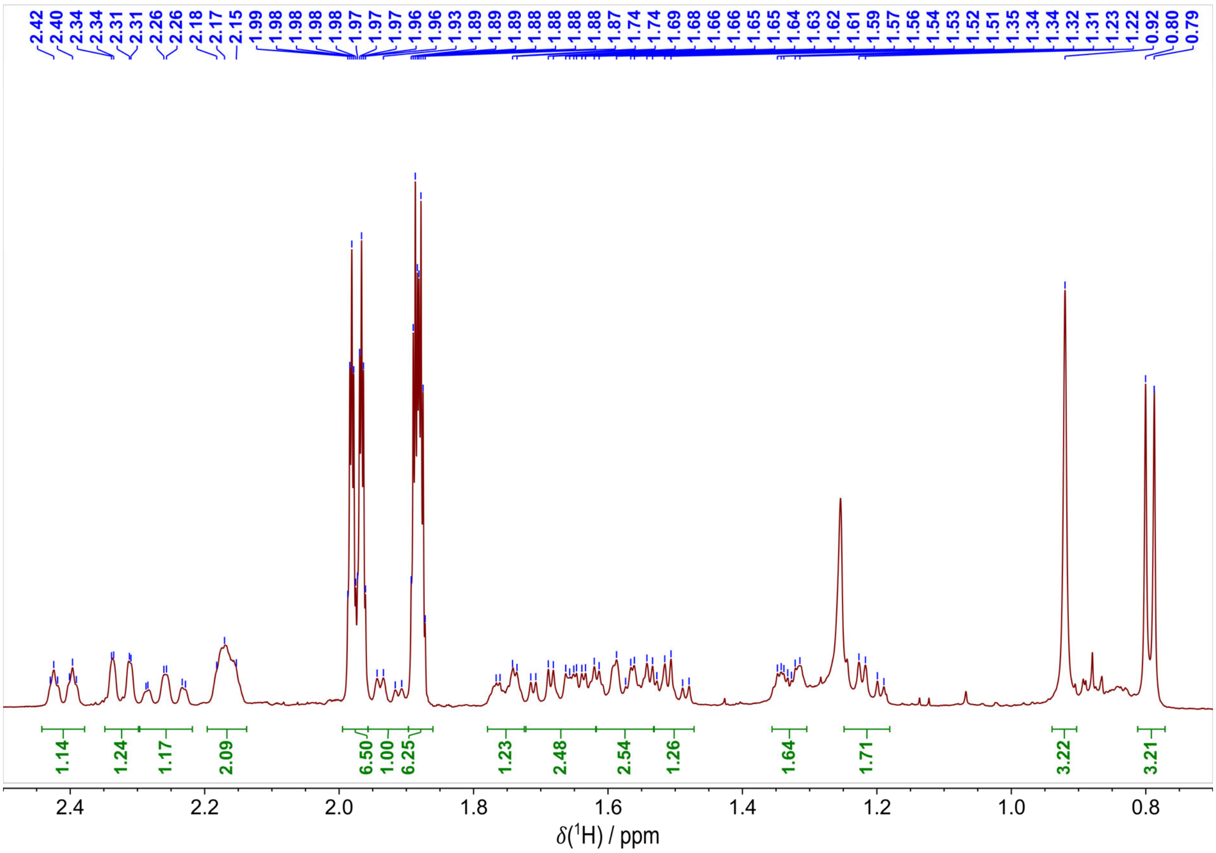

Figure S3. Partial ^{1}H NMR spectrum of solidagoic acid J (**1**) ($CDCl_3$, 500 MHz) – aliphatic region (δ_H 2.5–0.7).

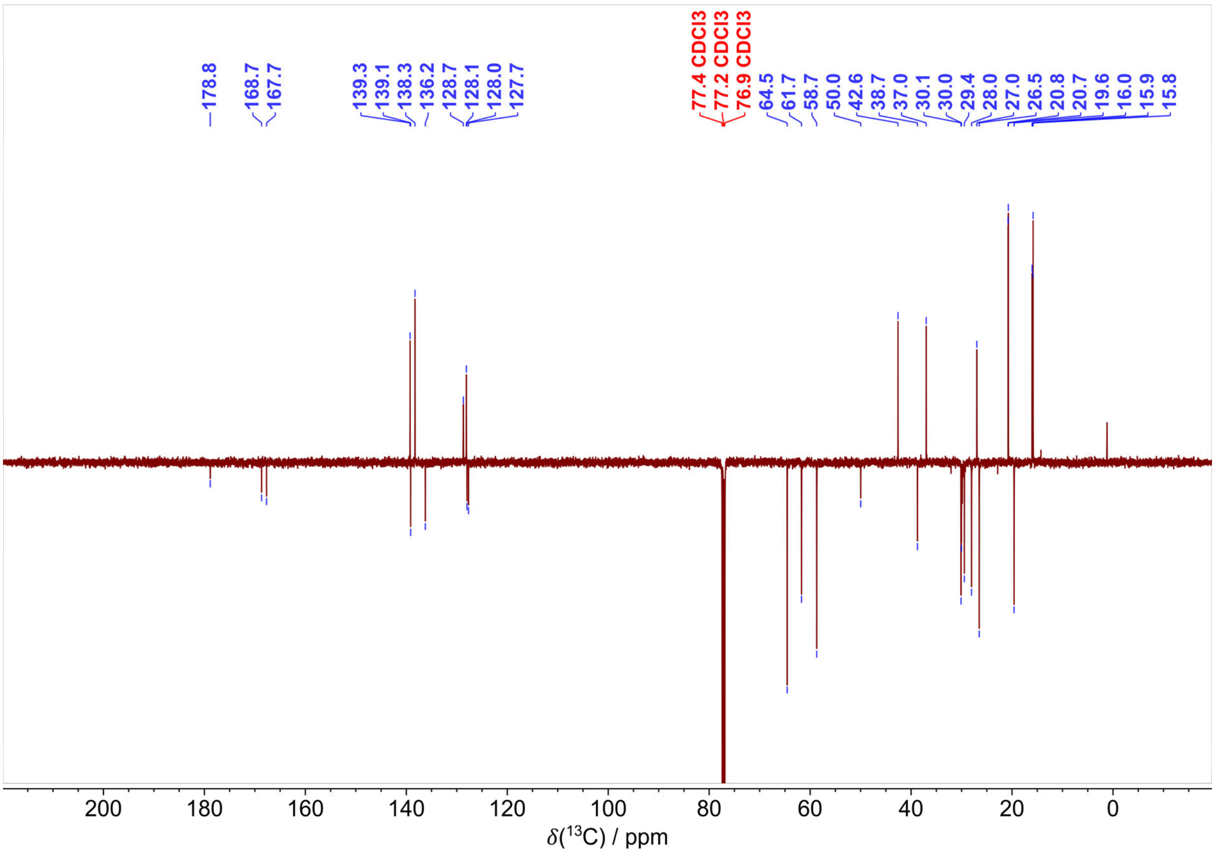

Figure S4. ^{13}C DEPTQ NMR spectrum of solidagoic acid J (**1**) ($CDCl_3$, 126 MHz).

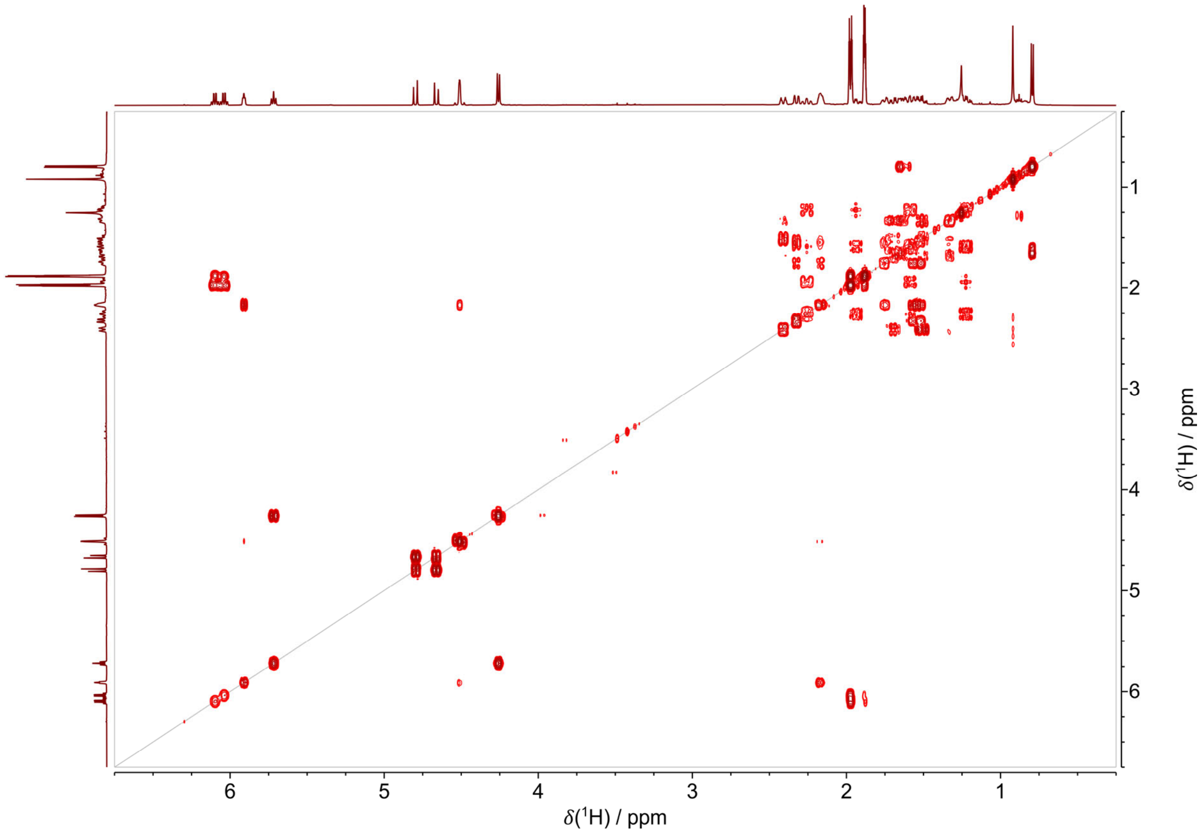

Figure S5. ^{1}H–^{1}H COSY NMR spectrum of solidagoic acid J (**1**) ($CDCl_3$, 500 MHz).

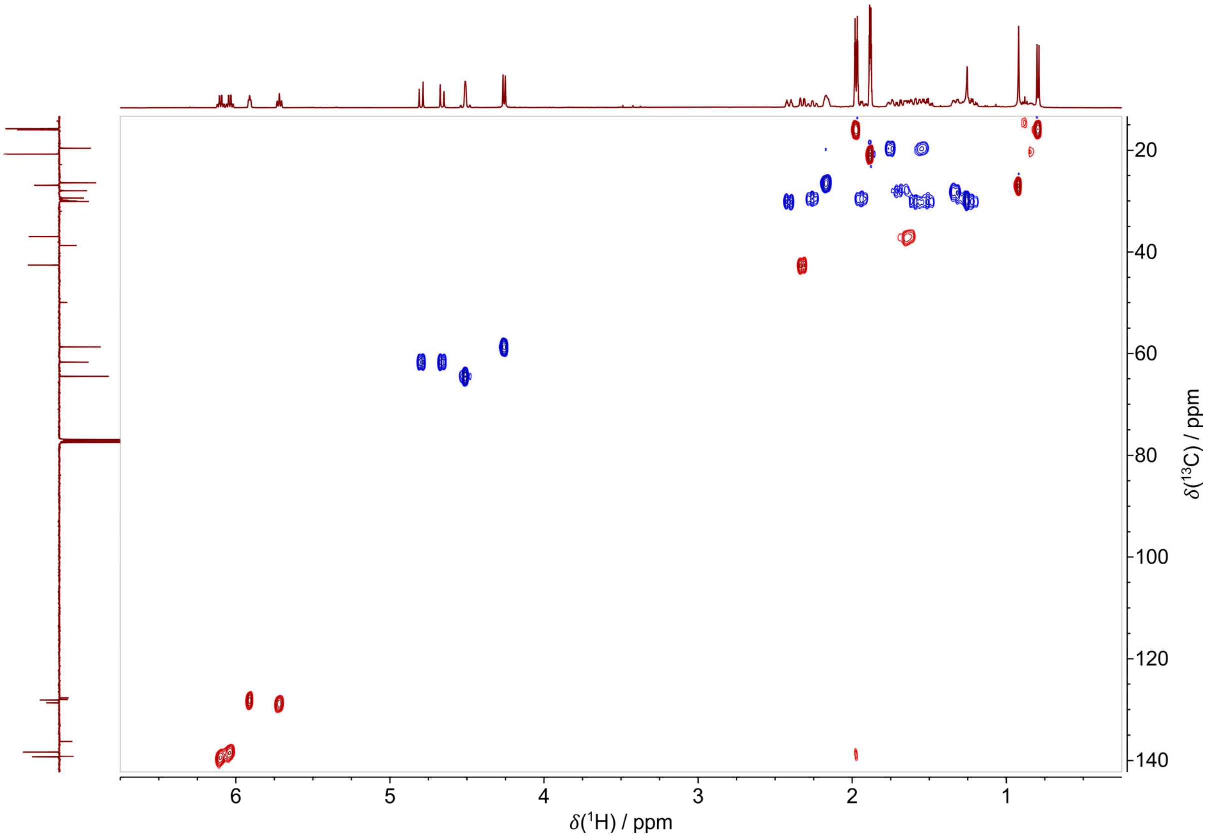

Figure S6. ^{1}H–^{13}C edHSQC NMR spectrum of solidagoic acid J (**1**) ($CDCl_3$, 500/126 MHz).

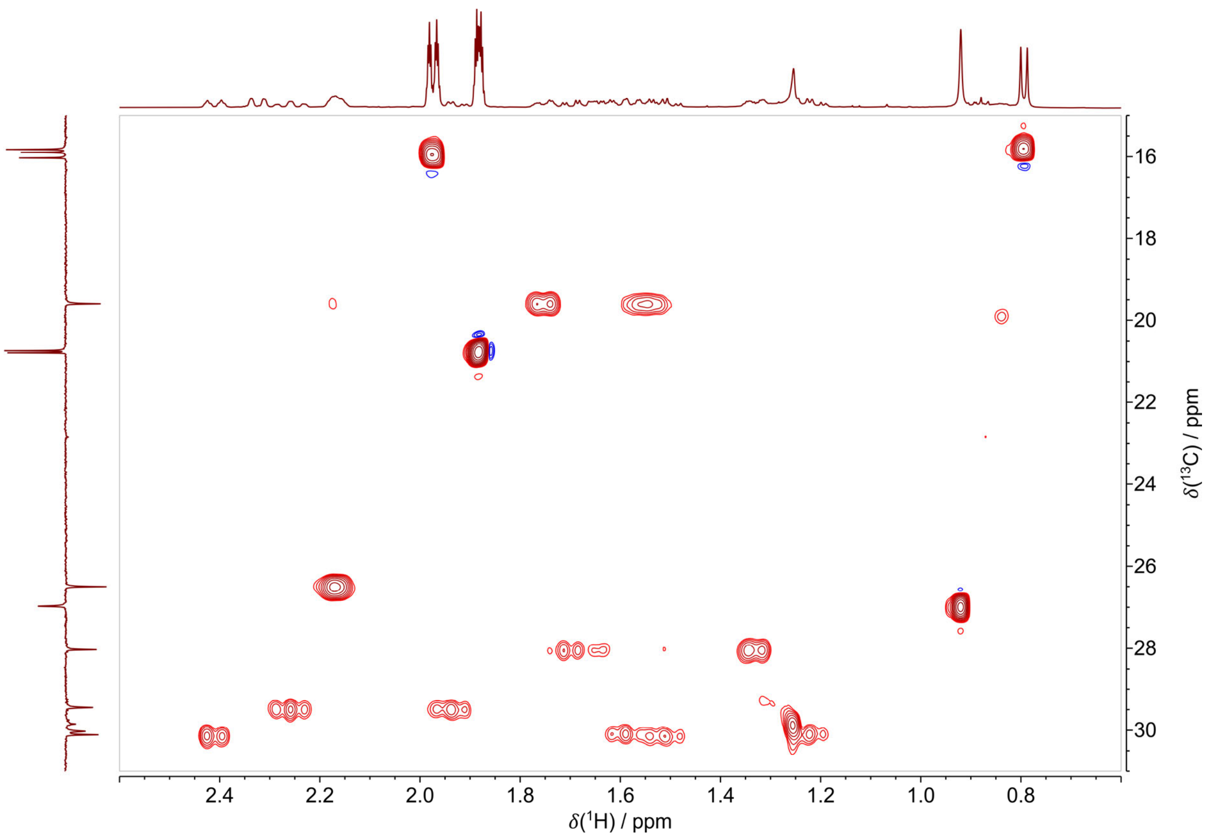

Figure S7. ^{1}H–^{13}C band-selective HSQC NMR spectrum of solidagoic acid J (**1**) ($CDCl_3$, 500/126 MHz) – aliphatic region (δ_C 31.0–15.0).

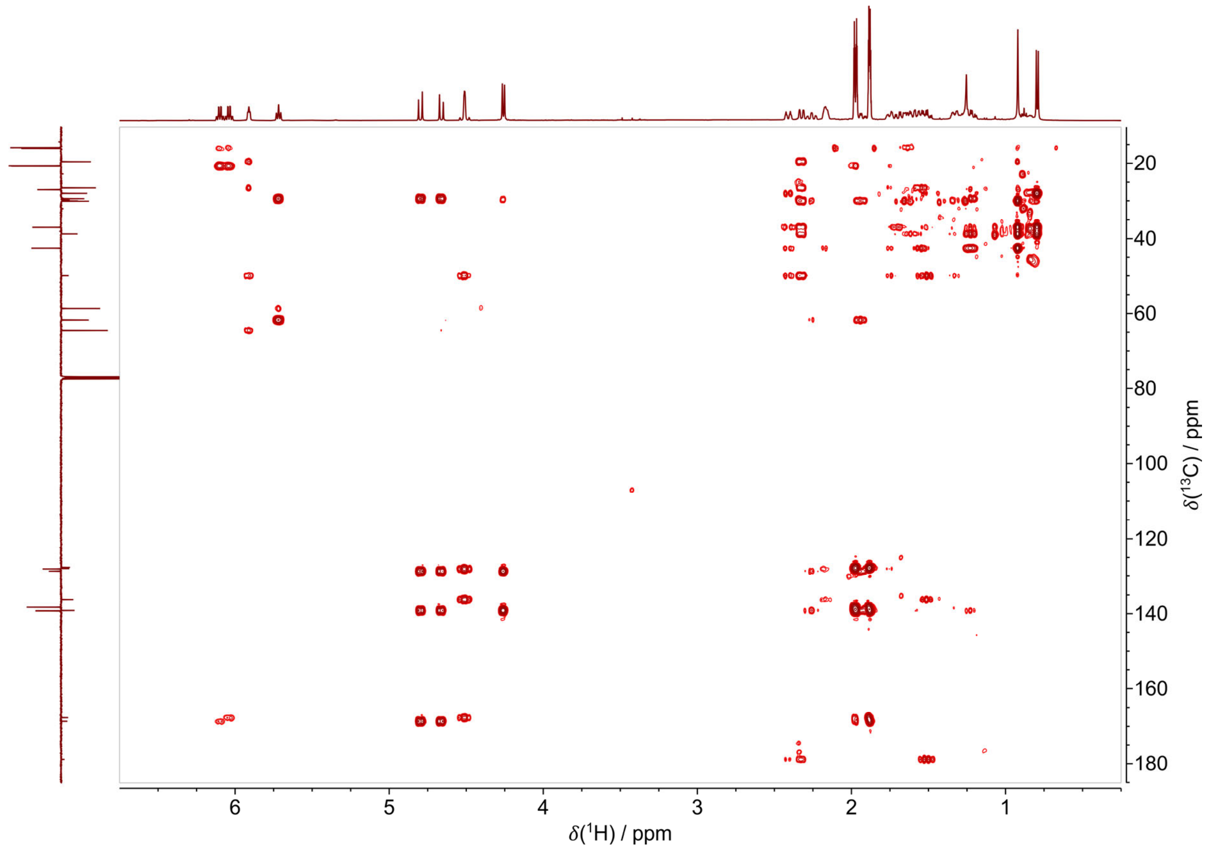

Figure S8. ^{1}H–^{13}C HMBC NMR spectrum of solidagoic acid J (**1**) ($CDCl_3$, 500/126 MHz).

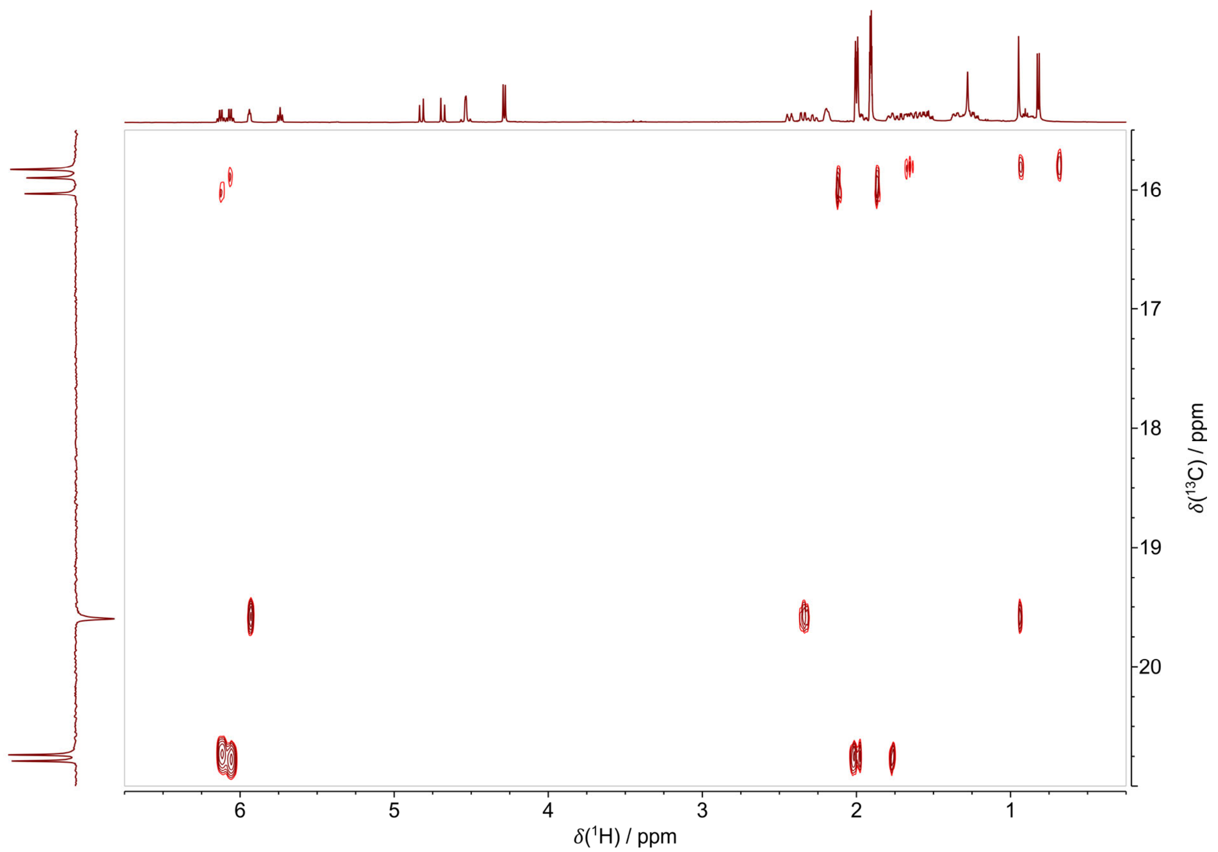

Figure S9. ^{1}H–^{13}C band-selective HMBC NMR spectrum of solidagoic acid J (**1**) ($CDCl_3$, 500/126 MHz) – aliphatic region (δ_C 21.0–15.5).

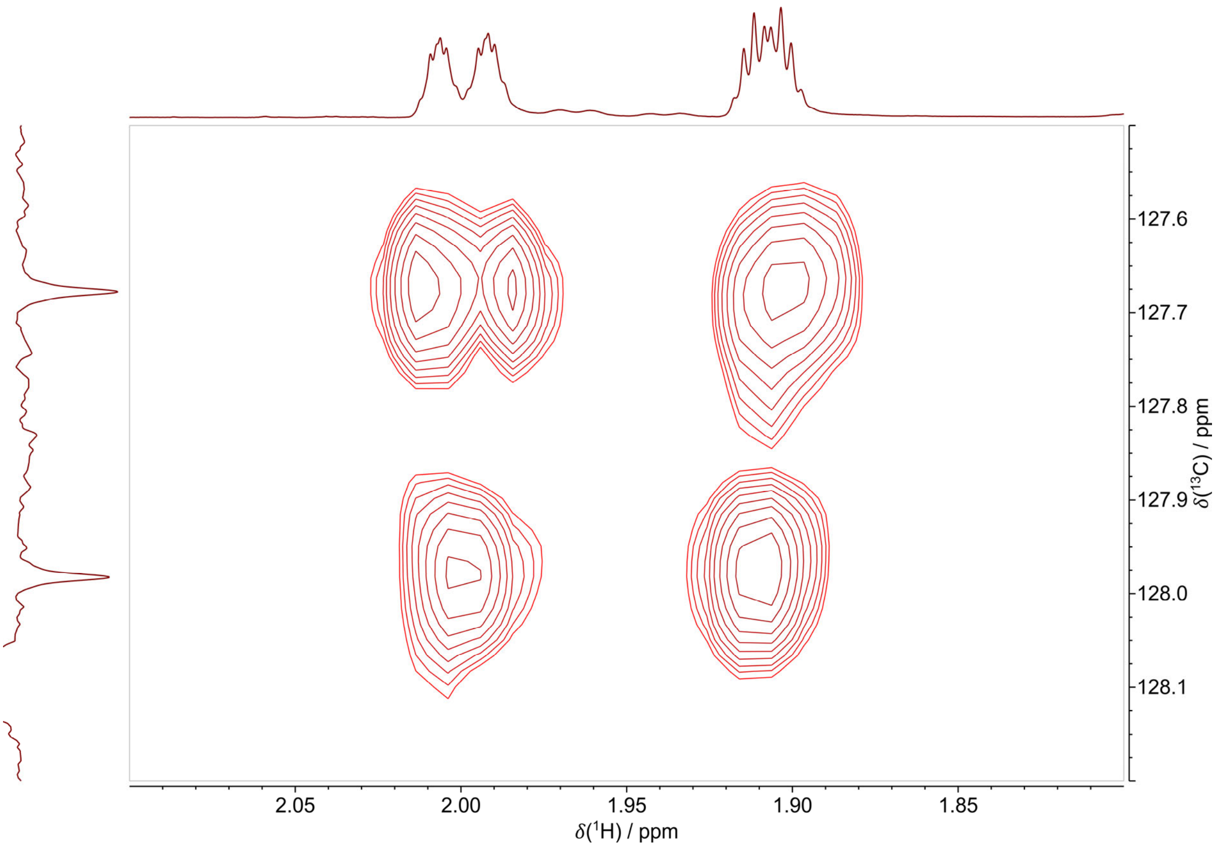

Figure S10. ^{1}H–^{13}C band-selective HMBC NMR spectrum of solidagoic acid J (**1**) ($CDCl_3$, 500/126 MHz) – double bond region (δ_C 128.2–127.5).

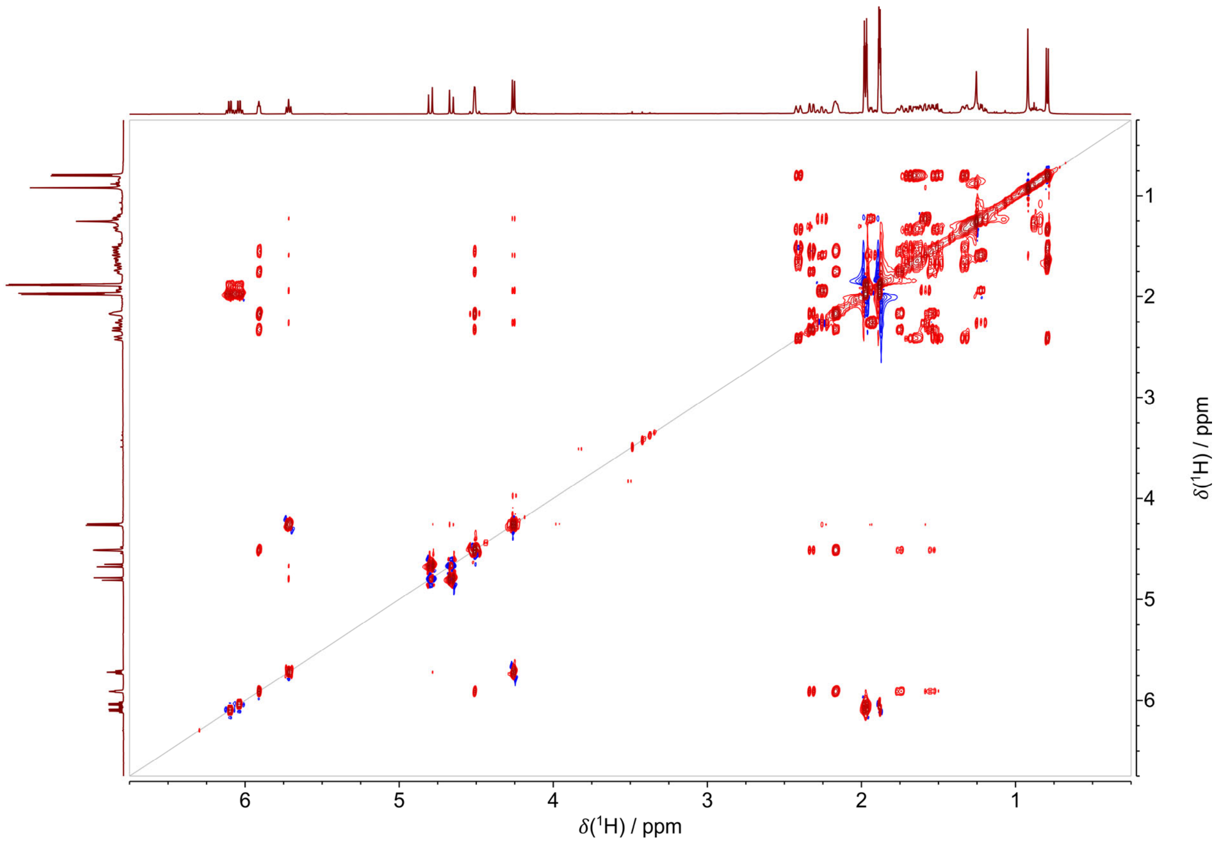

Figure S11. ^{1}H–^{1}H TOCSY NMR spectrum of solidagoic acid J (**1**) ($CDCl_3$, 500 MHz).

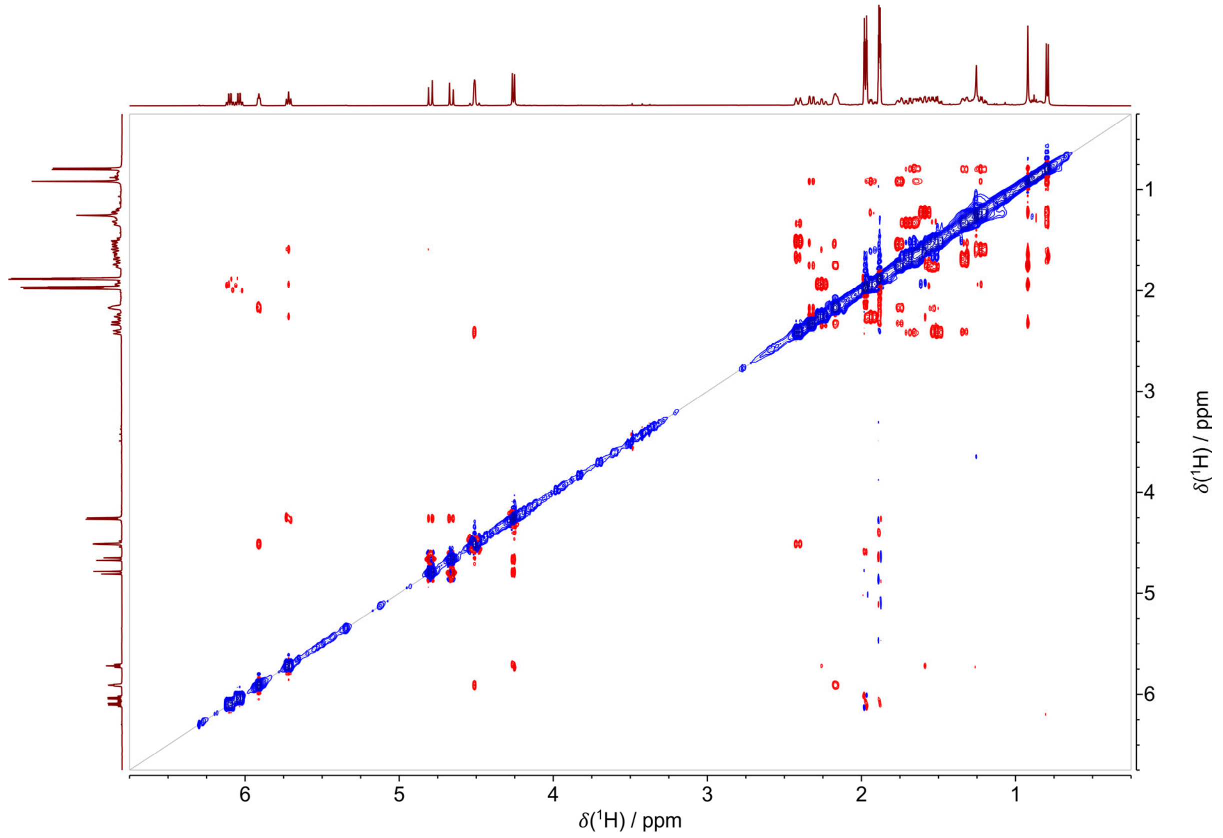

Figure S12. ^{1}H–^{1}H ROESY NMR spectrum of solidagoic acid J (**1**) ($CDCl_3$, 500 MHz).

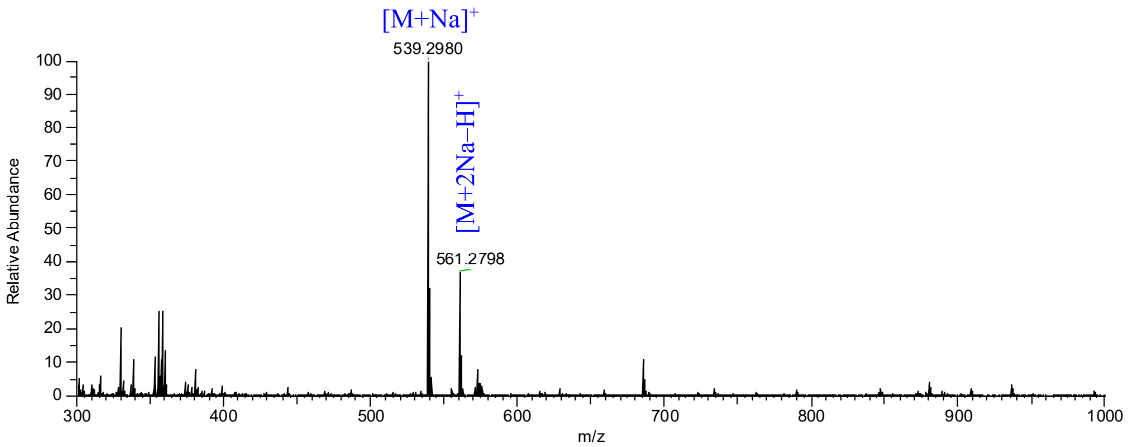

Figure S13. HR-ESI$^+$-MS spectrum of solidagoic acid J (**1**), *m/z* 539.2980 $[M+Na]^+$ (calculated for $C_{30}H_{44}O_7Na^+$, *m/z* 539.2979 $[M+Na]^+$, error: 0.1 ppm).

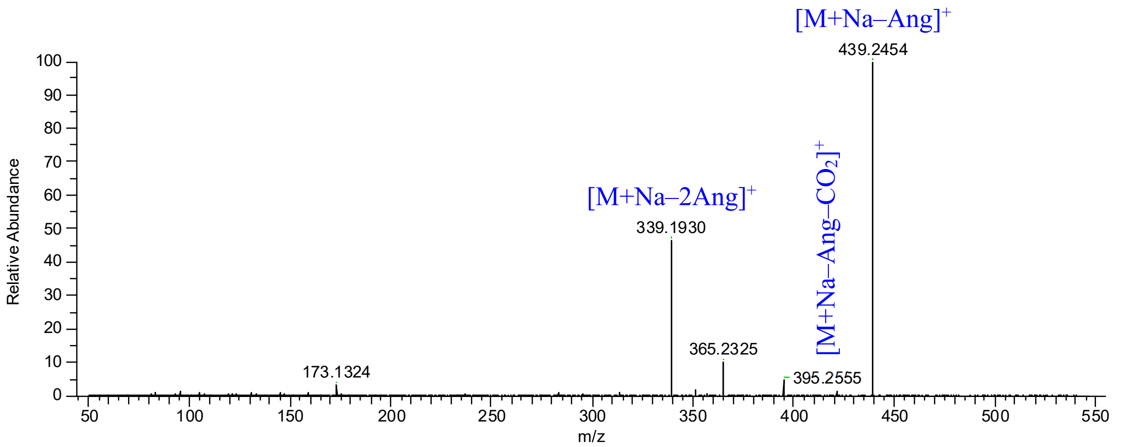

Figure S14. HR-ESI$^+$-MS/MS of solidagoic acid J (**1**) with a normalized HCD collision energy of 40%. Precursor ion: *m/z* 539.2979 $[M+Na]^+$, $C_{30}H_{44}O_7Na^+$. Ang denotes an angelate group ($C_5H_8O_2$).

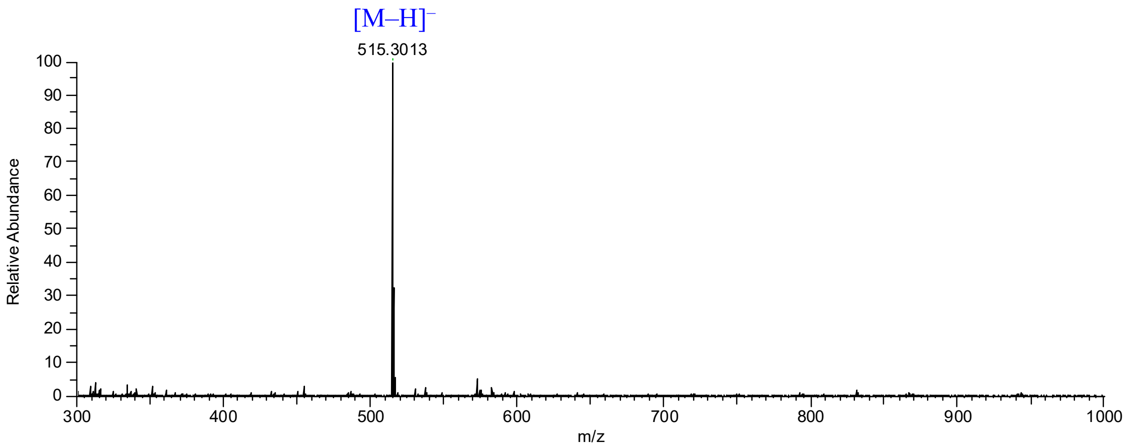

Figure S15. HR-ESI$^-$-MS spectrum of solidagoic acid J (**1**), *m/z* 515.3013 $[M–H]^-$ (calculated for $C_{30}H_{43}O_7^-$, *m/z* 515.3014 $[M–H]^-$, error: –0.3 ppm).

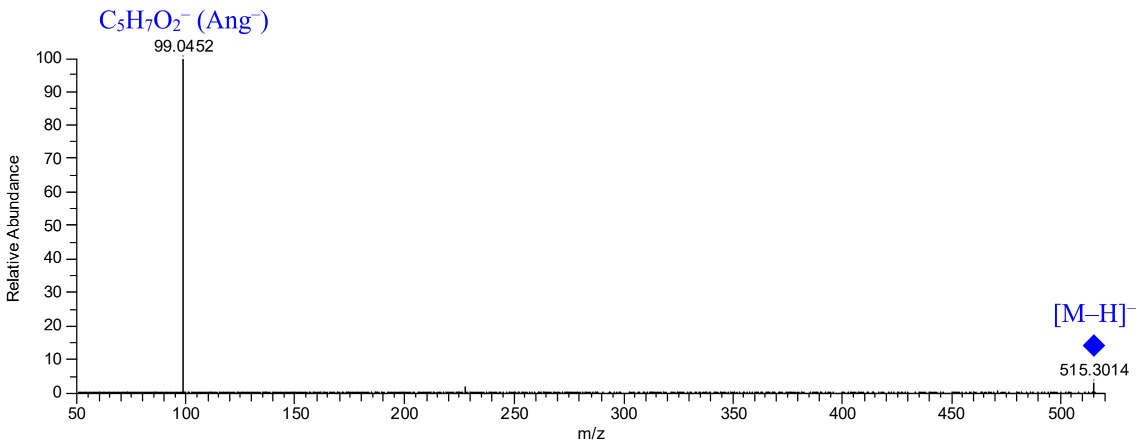

Figure S16. HR-ESI⁻-MS/MS of solidagoic acid J (**1**) with a normalized HCD collision energy of 40%.

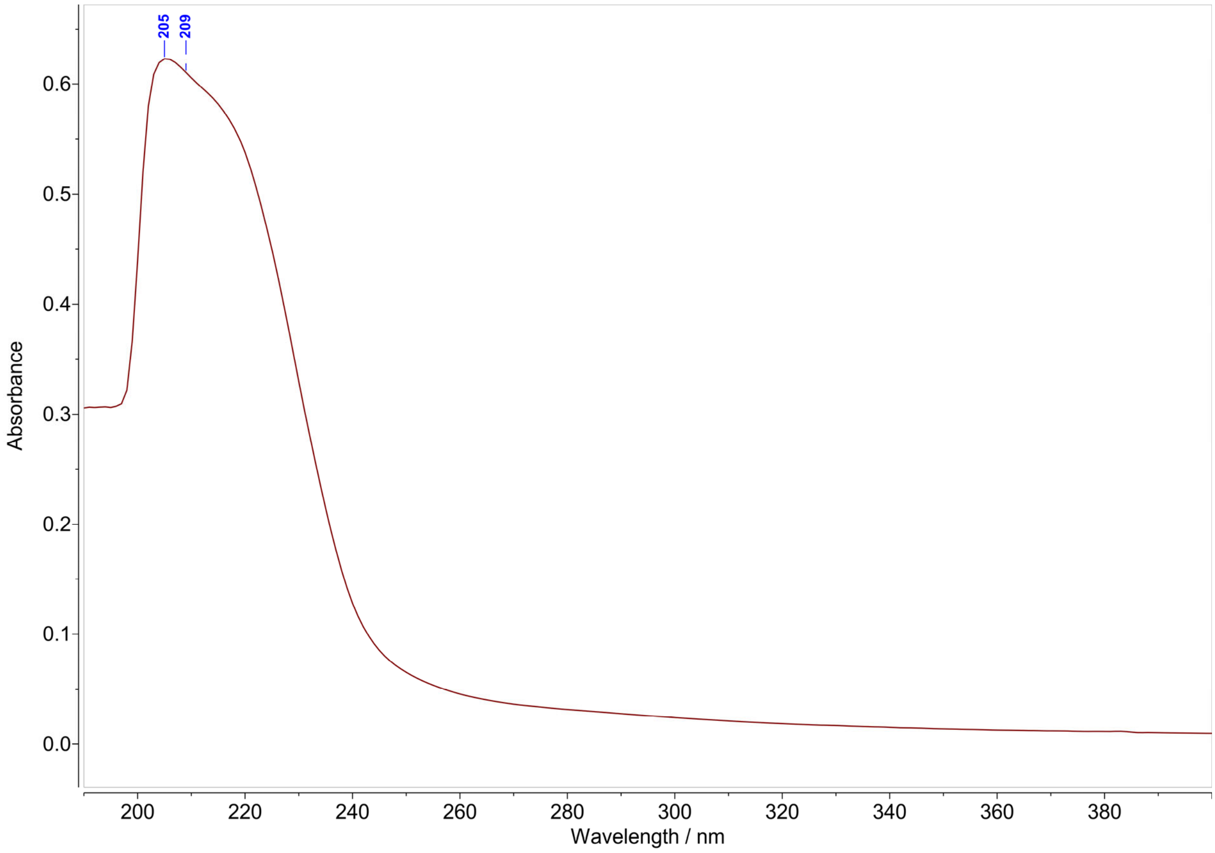

Figure S17. UV spectrum of solidagoic acid J (**1**) in ethanol.

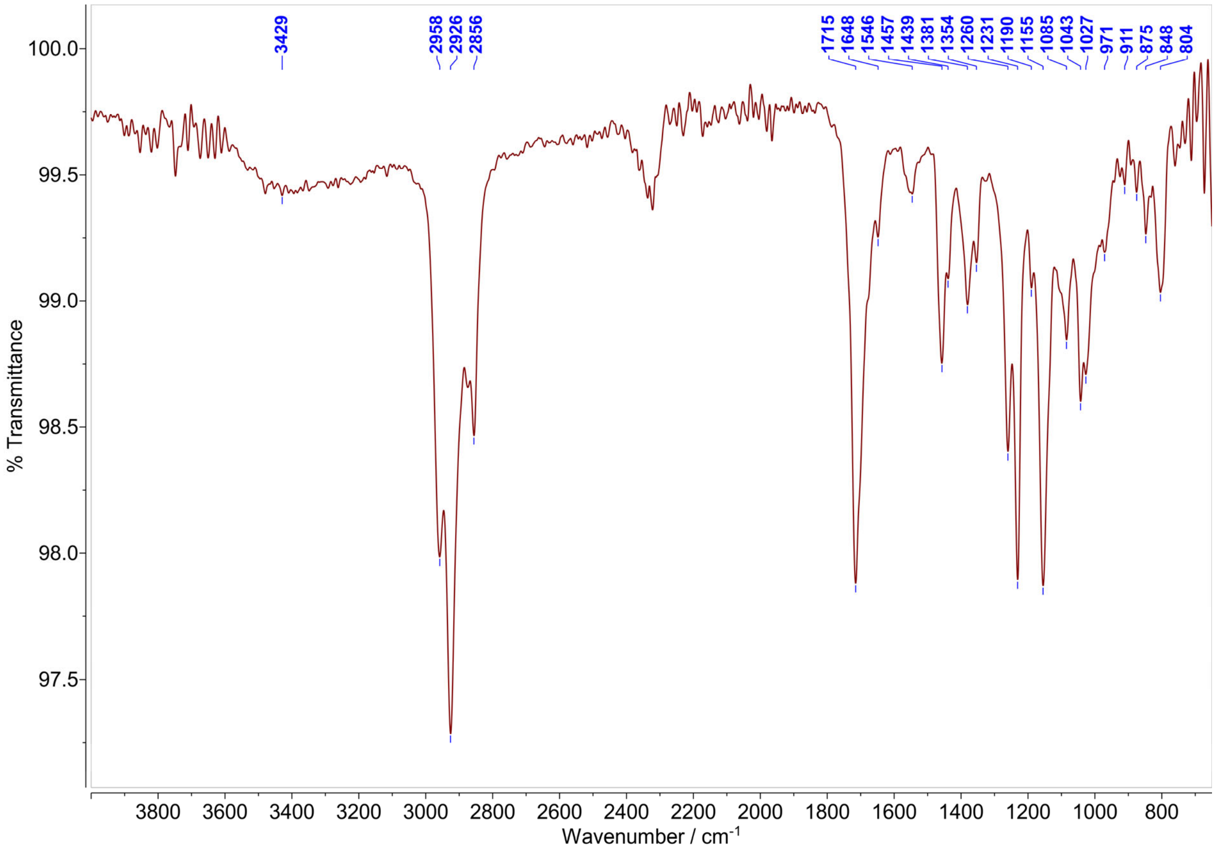

Figure S18. ATR-FTIR spectrum of solidagoic acid J (**1**).

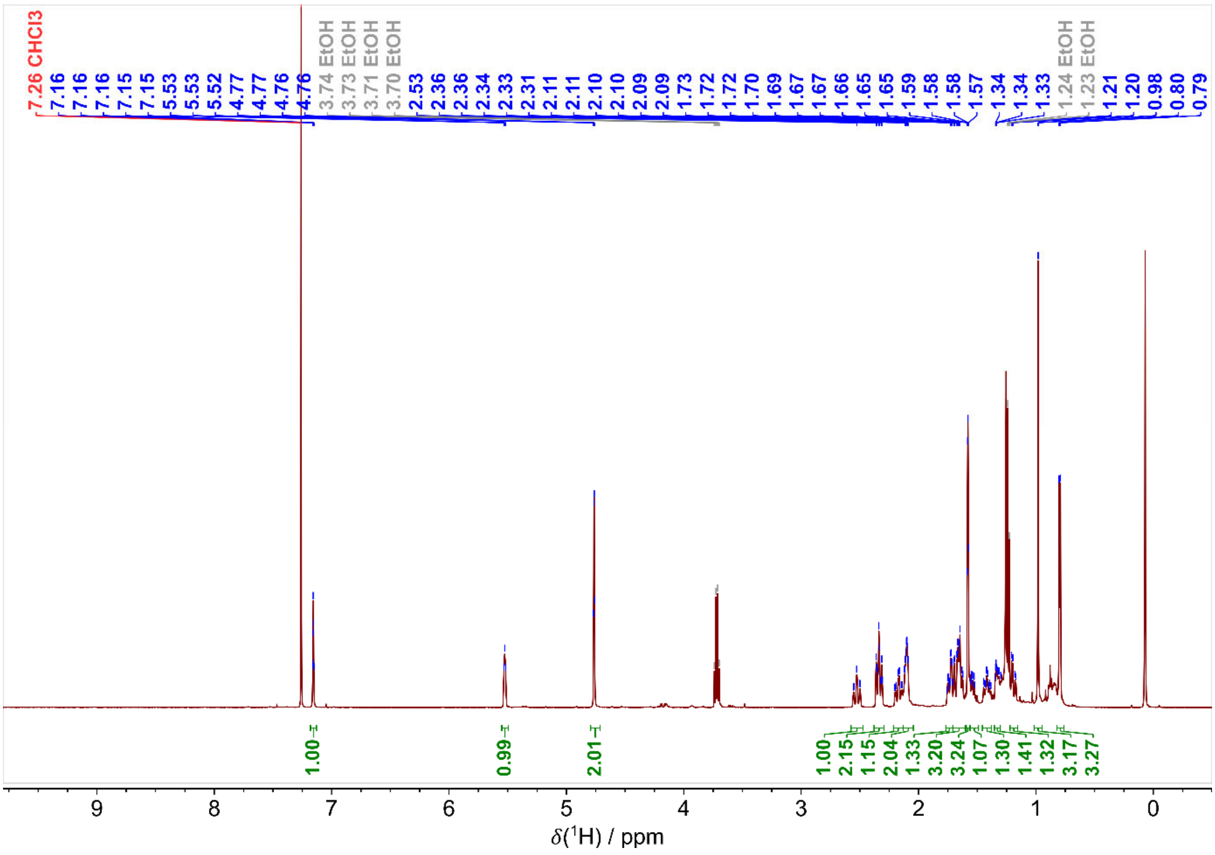

Figure S19. ^{1}H NMR spectrum of solidagoic acid C (**2**) ($CDCl_3$, 500 MHz).

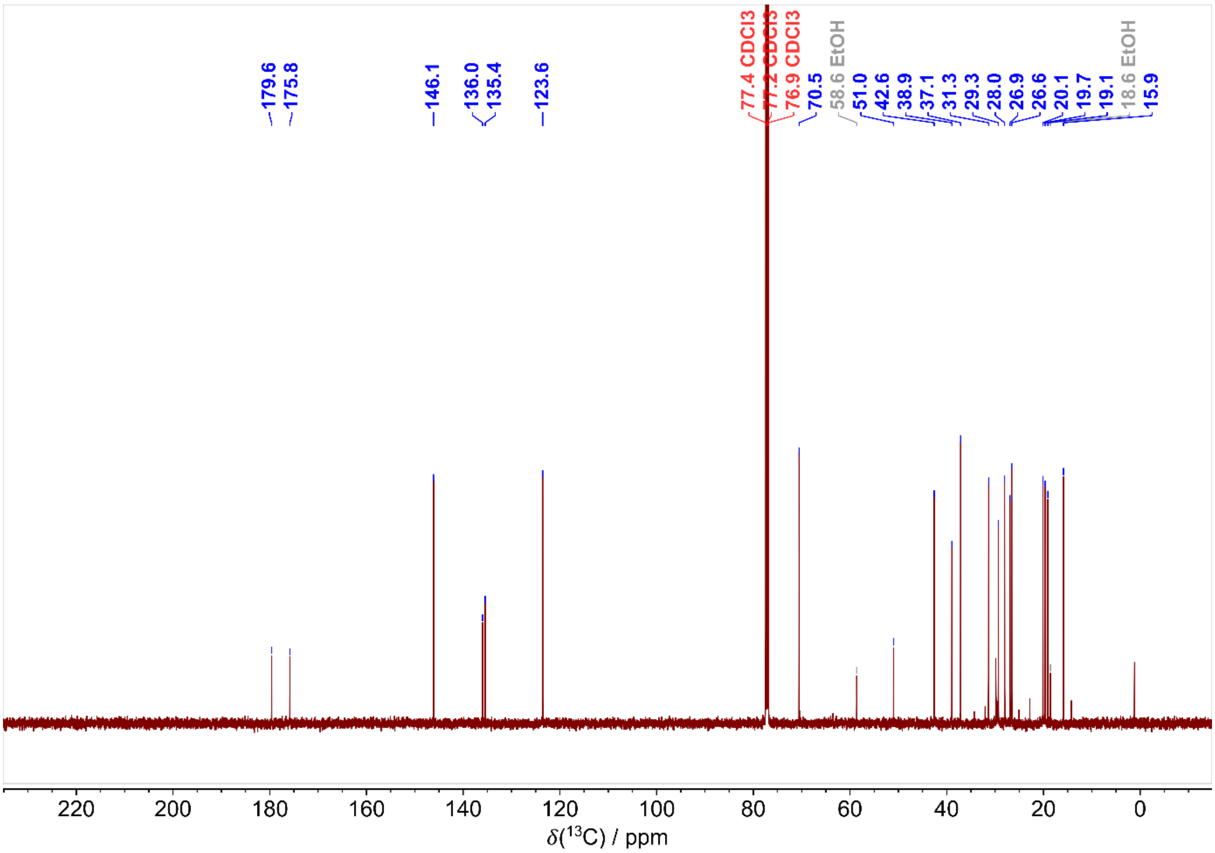

Figure S20. ^{13}C NMR spectrum of solidagoic acid C (**2**) ($CDCl_3$, 126 MHz).

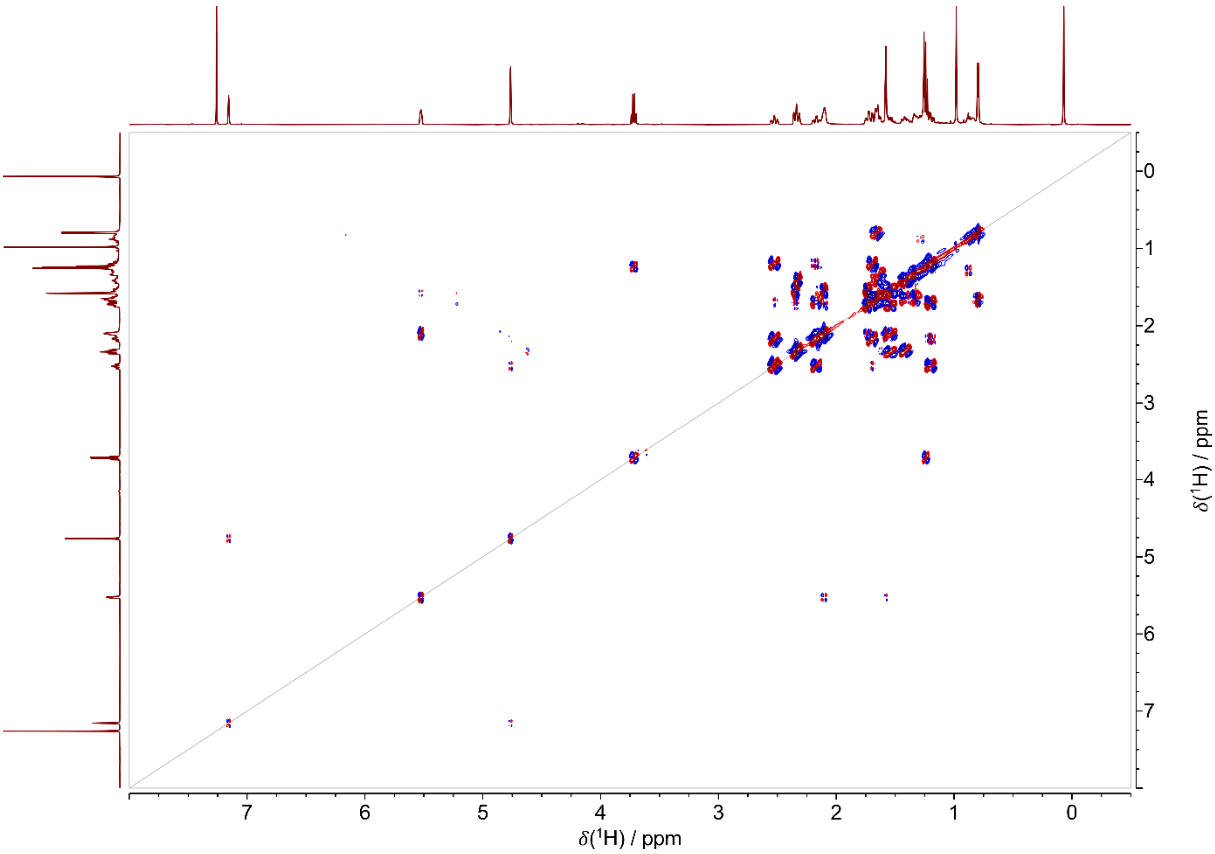

Figure S21. ^{1}H–^{1}H DQF-COSY NMR spectrum of solidagoic acid C (**2**) ($CDCl_3$, 500 MHz).

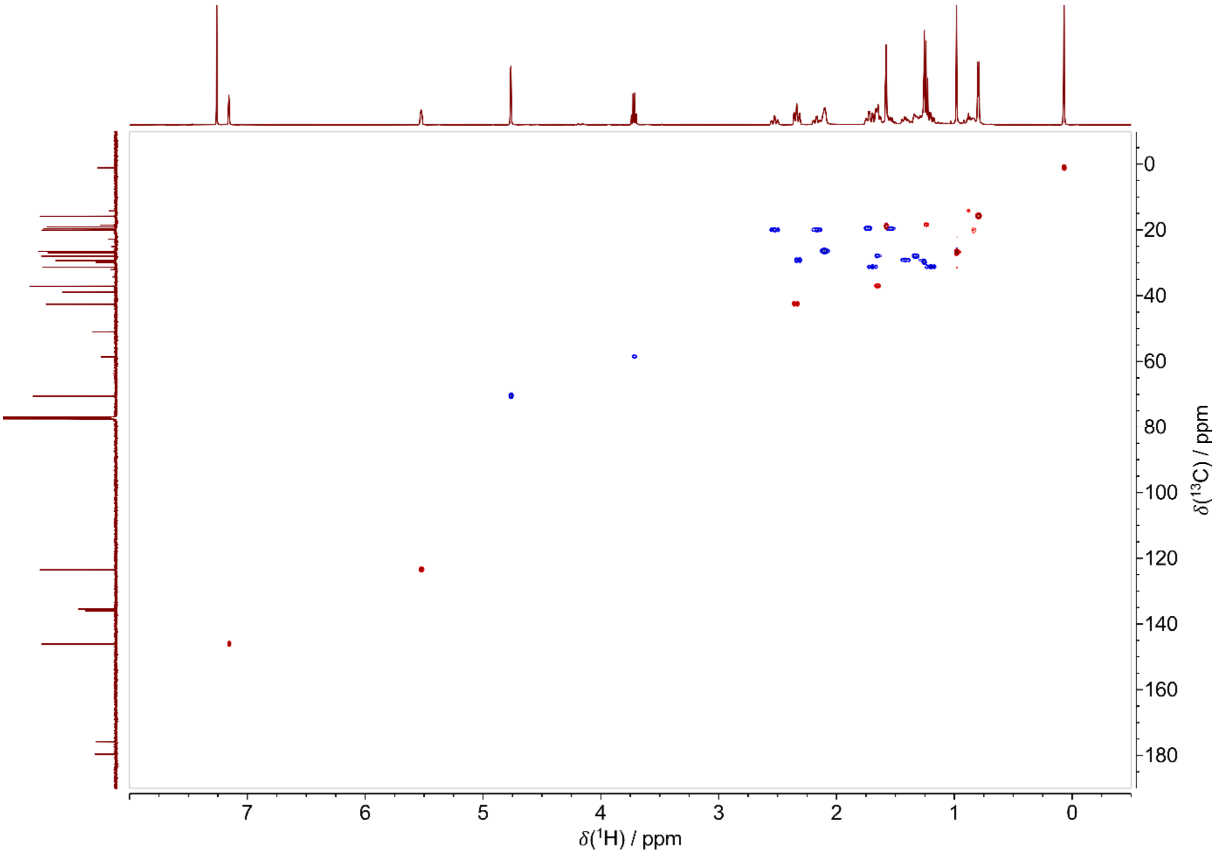

Figure S22. ¹H–¹³C edHSQC NMR spectrum of solidagoic acid C (**2**) ($CDCl_3$, 500/126 MHz).

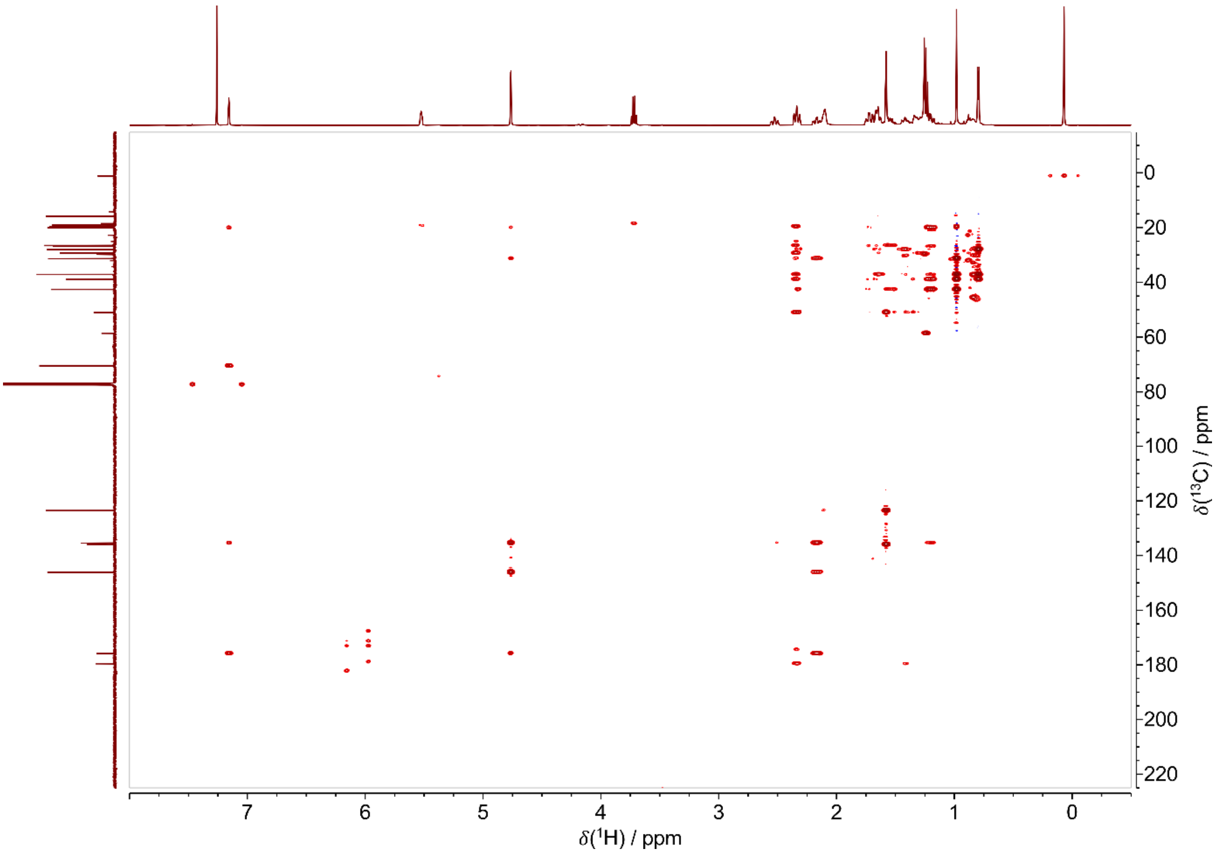

Figure S23. ¹H–¹³C HMBC NMR spectrum of solidagoic acid C (**2**) ($CDCl_3$, 500/126 MHz).

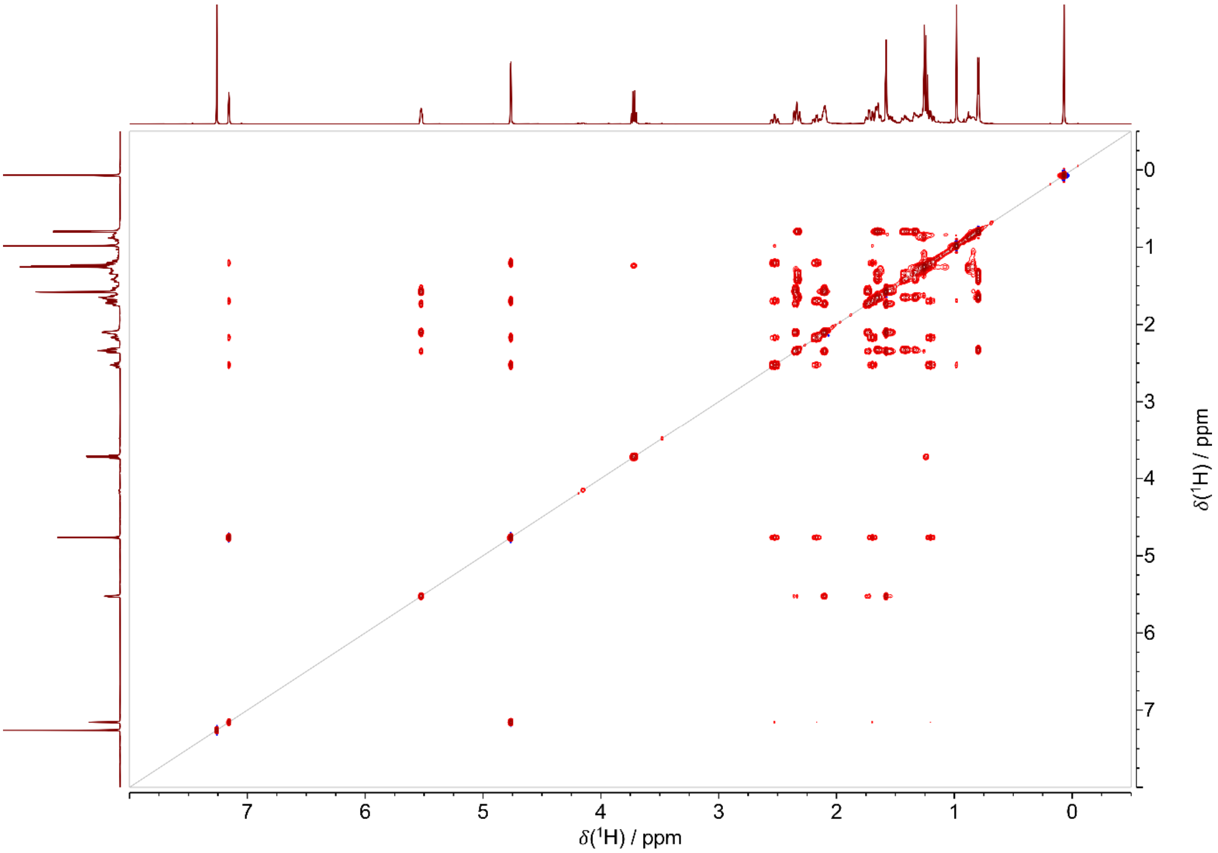

Figure S24. ^{1}H–^{1}H TOCSY NMR spectrum of solidagoic acid C (**2**) ($CDCl_3$, 500 MHz).

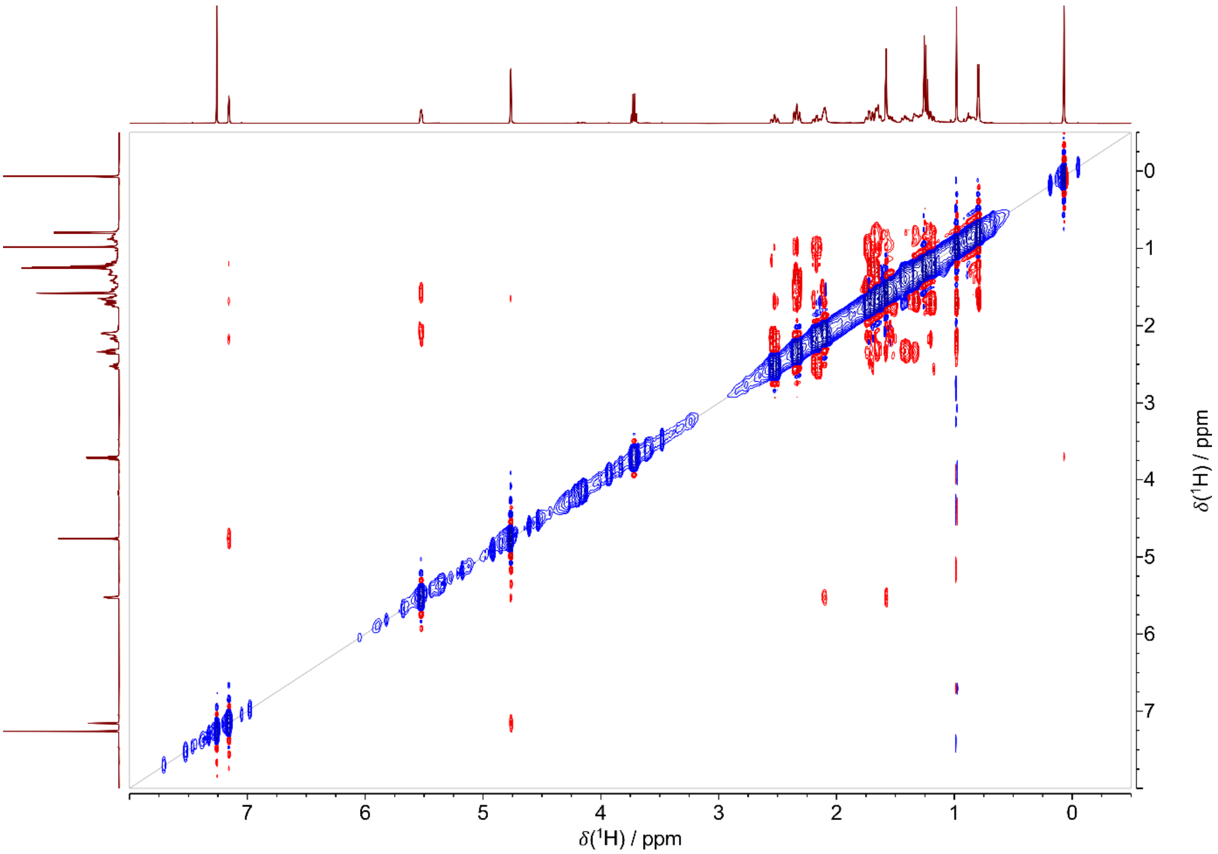

Figure S25. ^{1}H–^{1}H ROESY NMR spectrum of solidagoic acid C (**2**) ($CDCl_3$, 500 MHz).

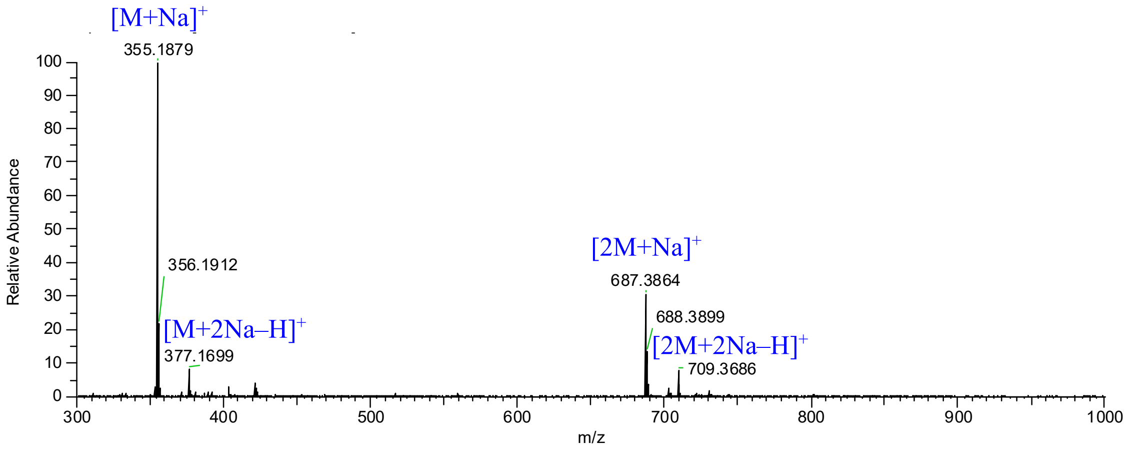

Figure S26. HR-ESI$^+$-MS spectrum of solidagoic acid C (**2**), *m/z* 355.1879 $[M+Na]^+$ (calculated for $C_{20}H_{28}O_4Na^+$, *m/z* 355.1880 $[M+Na]^+$, error: –0.1 ppm).

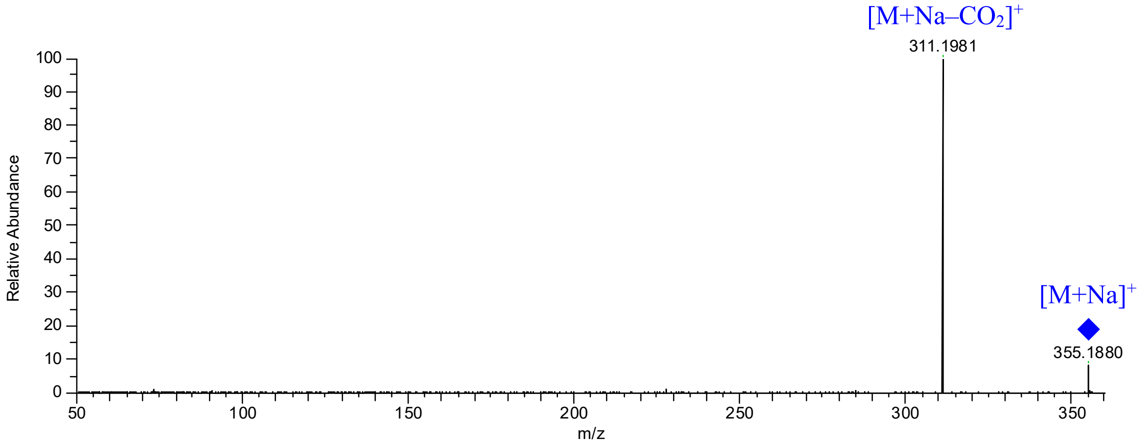

Figure S27. HR-ESI$^+$-MS/MS of solidagoic acid C (**2**) with a normalized HCD collision energy of 40%. Precursor ion: *m/z* 355.1880 $[M+Na]^+$, $C_{20}H_{28}O_4Na^+$.

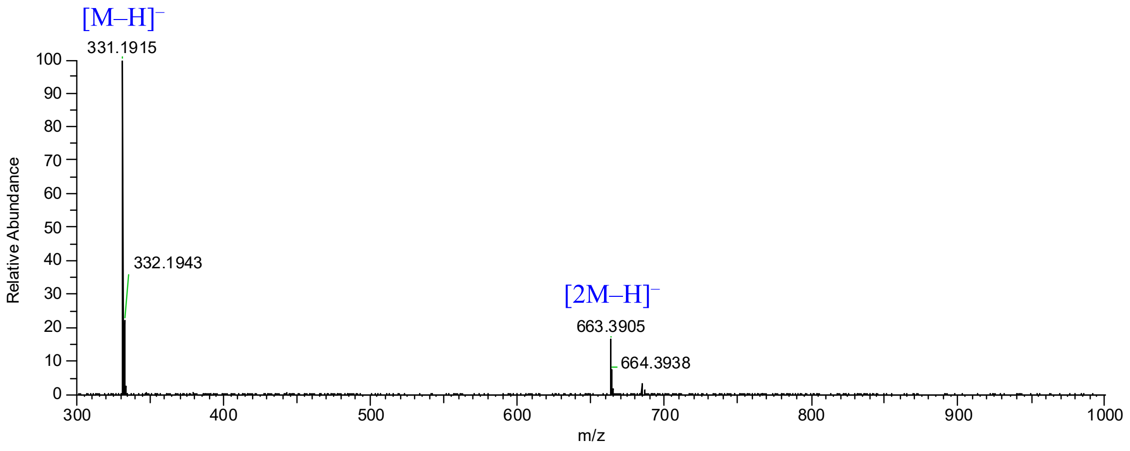

Figure S28. HR-ESI$^-$-MS spectrum of solidagoic acid C (**2**), *m/z* 331.1915 $[M–H]^-$ (calculated for $C_{20}H_{27}O_4^-$, *m/z* 331.1915 $[M–H]^-$, error: –0.1 ppm).

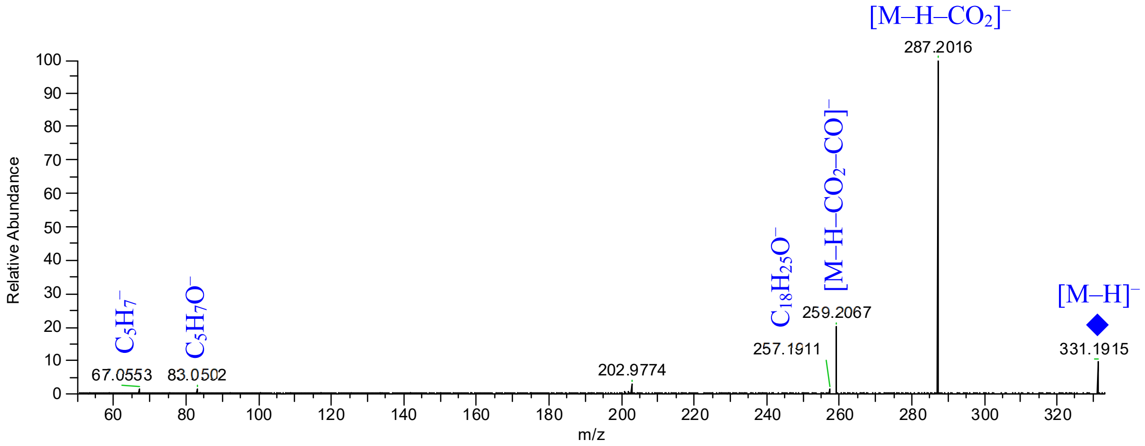

Figure S29. HR-ESI⁻-MS/MS of solidagoic acid C (**2**) with a normalized HCD collision energy of 40%. Precursor ion: *m*/*z* 331.1915 [M–H]⁻, $C_{20}H_{27}O_4^-$.

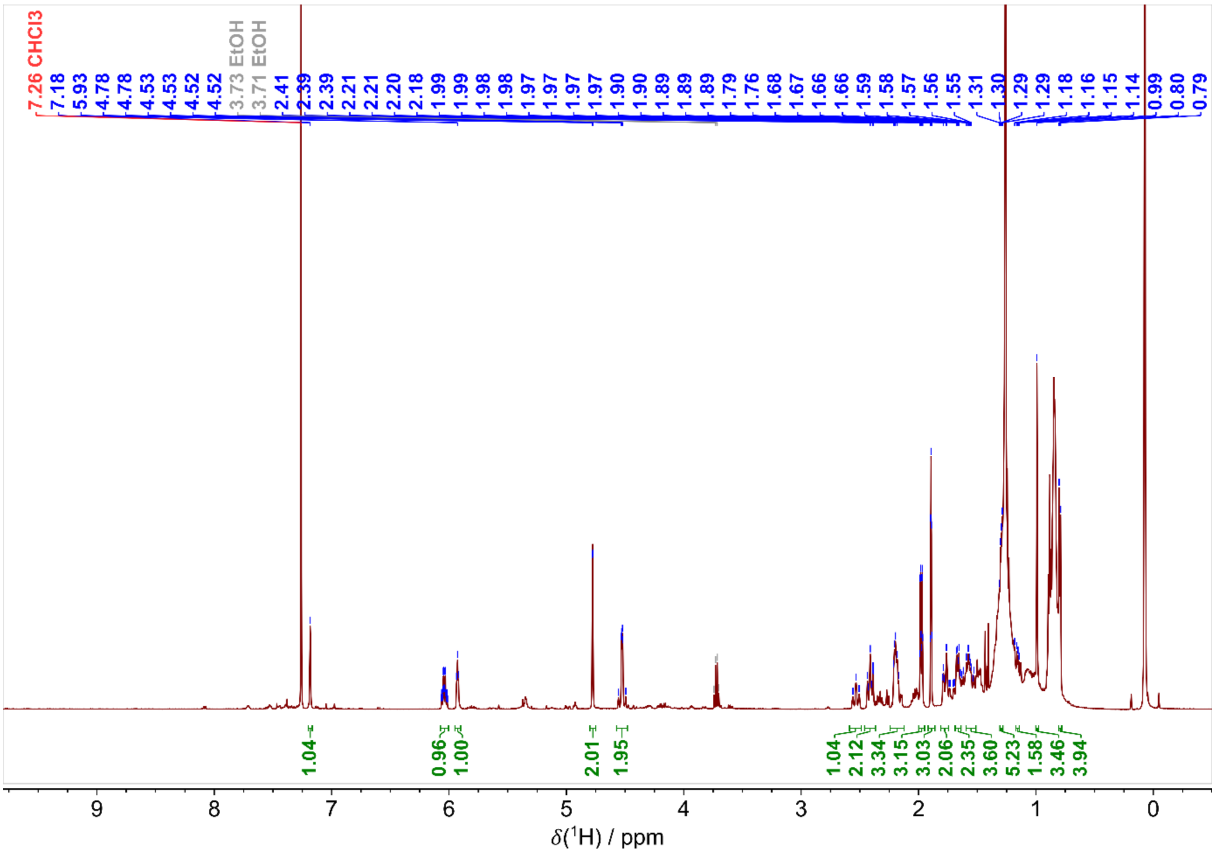

Figure S30. ^{1}H NMR spectrum of solidagoic acid D (**3**) ($CDCl_3$, 500 MHz).

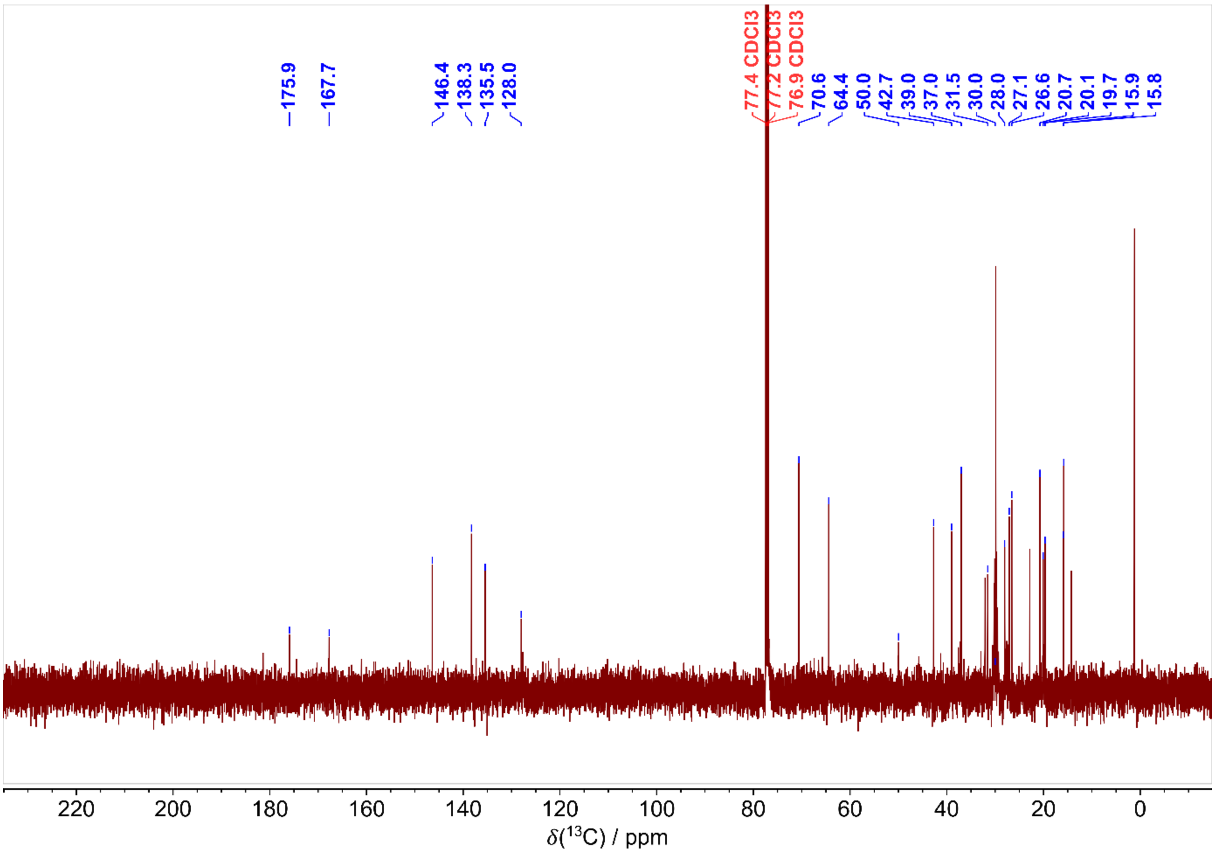

Figure S31. ^{13}C NMR spectrum of solidagoic acid D (**3**) ($CDCl_3$, 126 MHz).

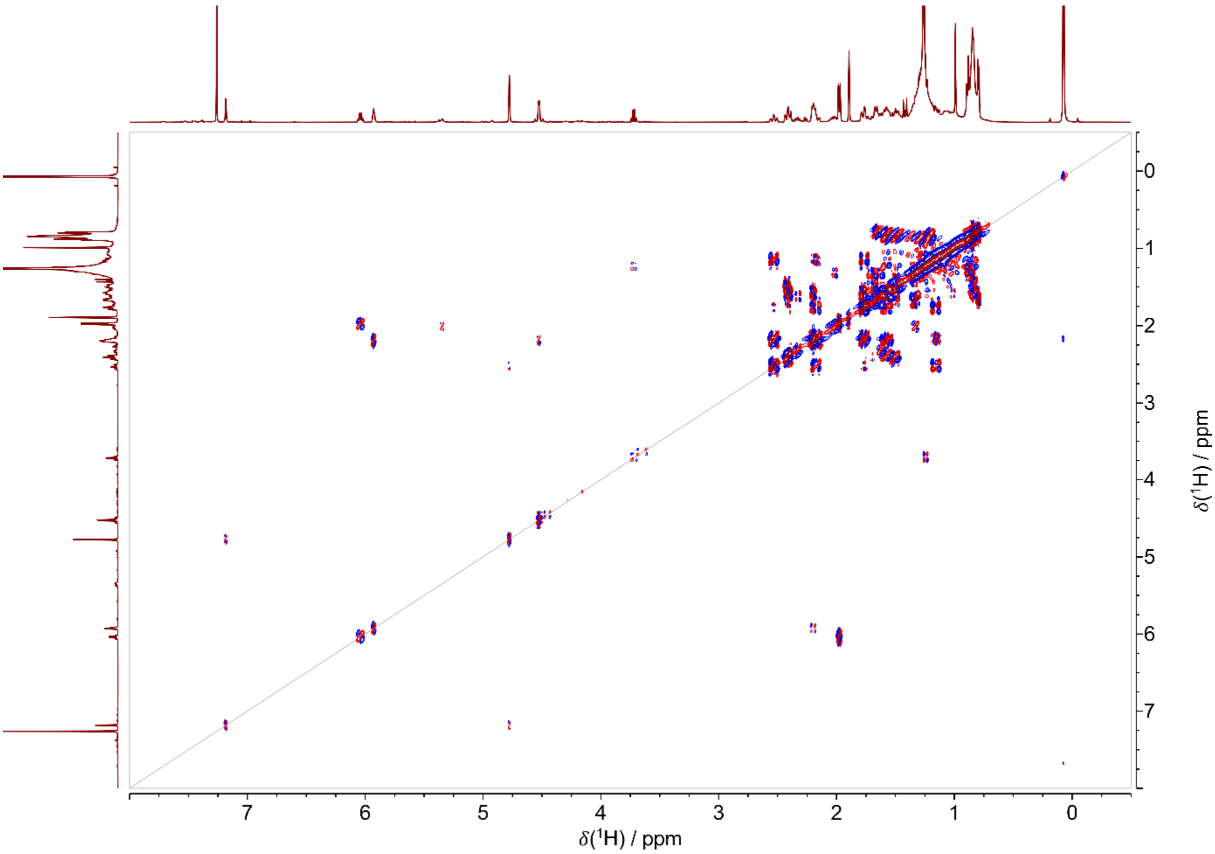

Figure S32. ^{1}H–^{1}H DQF-COSY NMR spectrum of solidagoic acid D (**3**) ($CDCl_3$, 500 MHz).

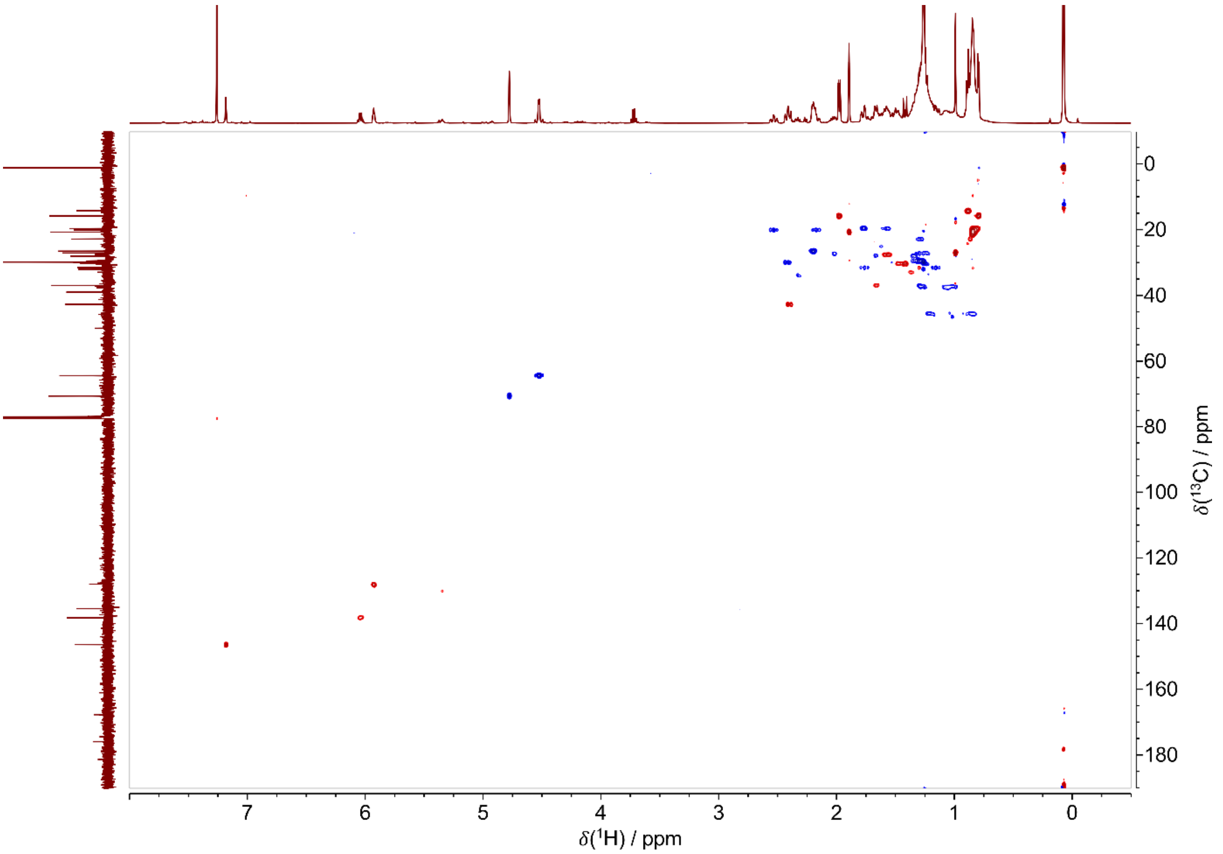

Figure S33. ¹H–¹³C edHSQC NMR spectrum of solidagoic acid D (**3**) (CDCl₃, 500/126 MHz).

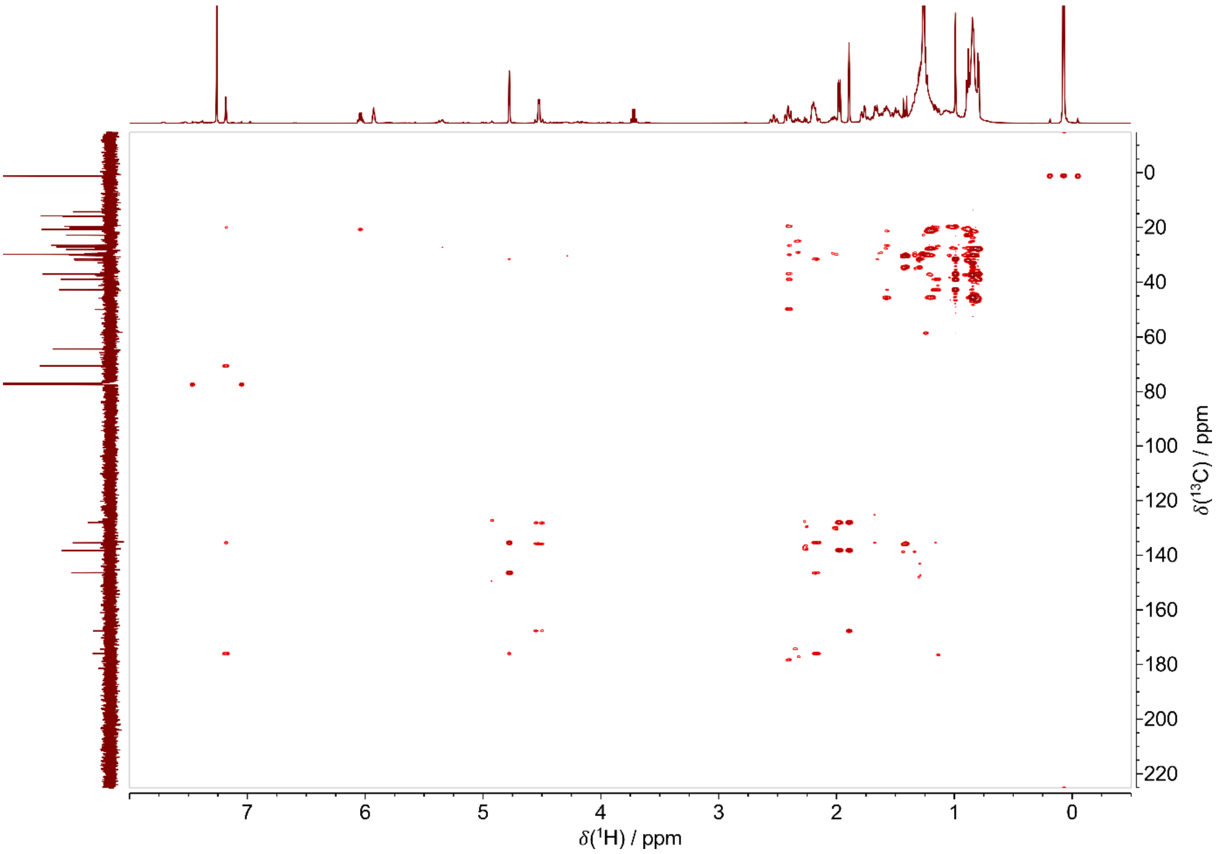

Figure S34. ¹H–¹³C HMBC NMR spectrum of solidagoic acid D (**3**) (CDCl₃, 500/126 MHz).

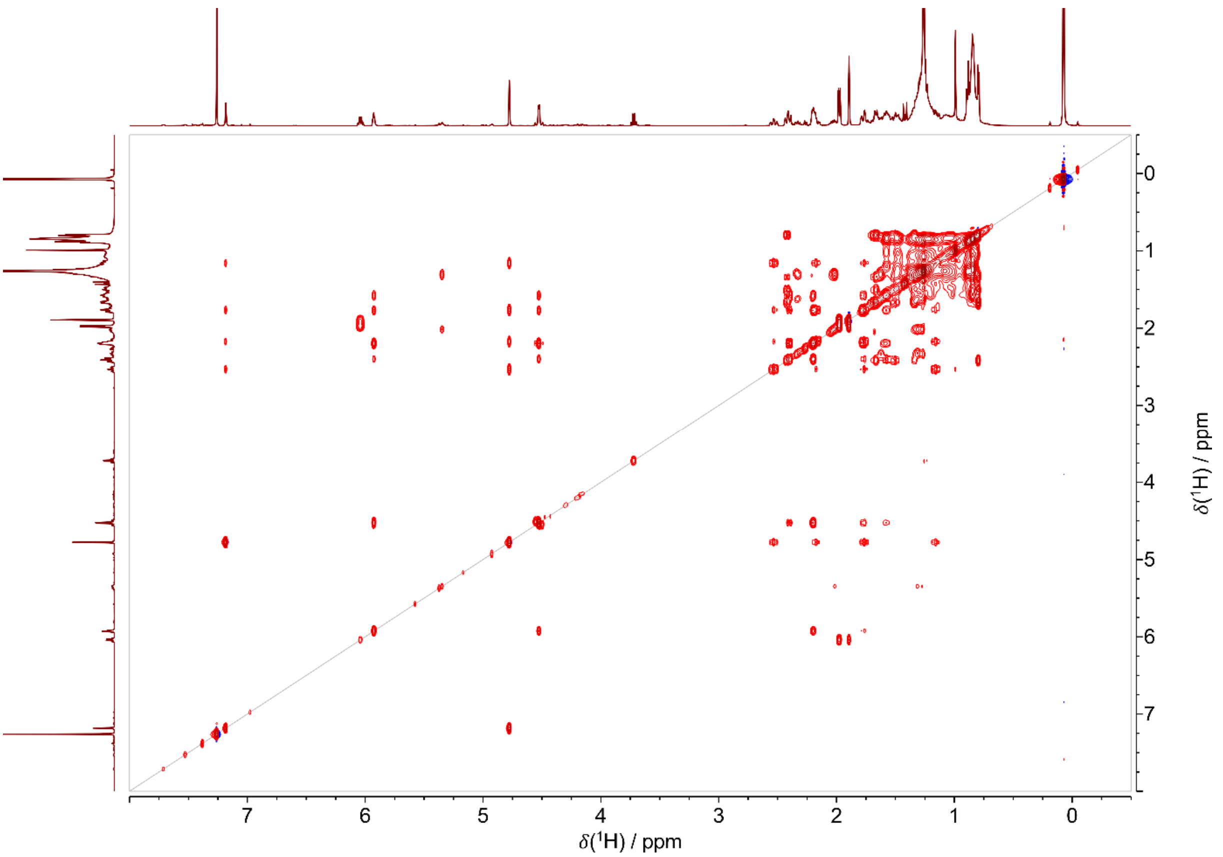

Figure S35. ^{1}H–^{1}H TOCSY NMR spectrum of solidagoic acid D (**3**) ($CDCl_3$, 500 MHz).

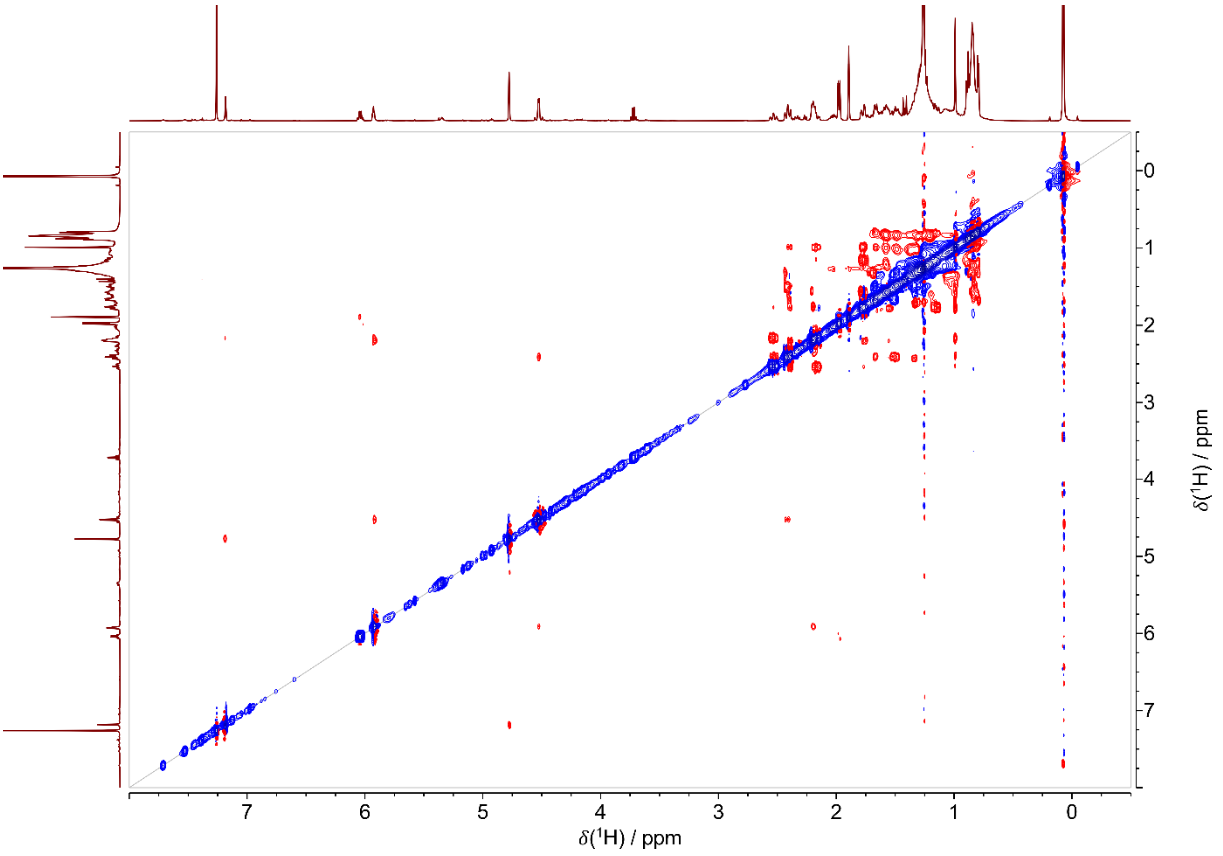

Figure S36. ^{1}H–^{1}H ROESY NMR spectrum of solidagoic acid D (**3**) ($CDCl_3$, 500 MHz).

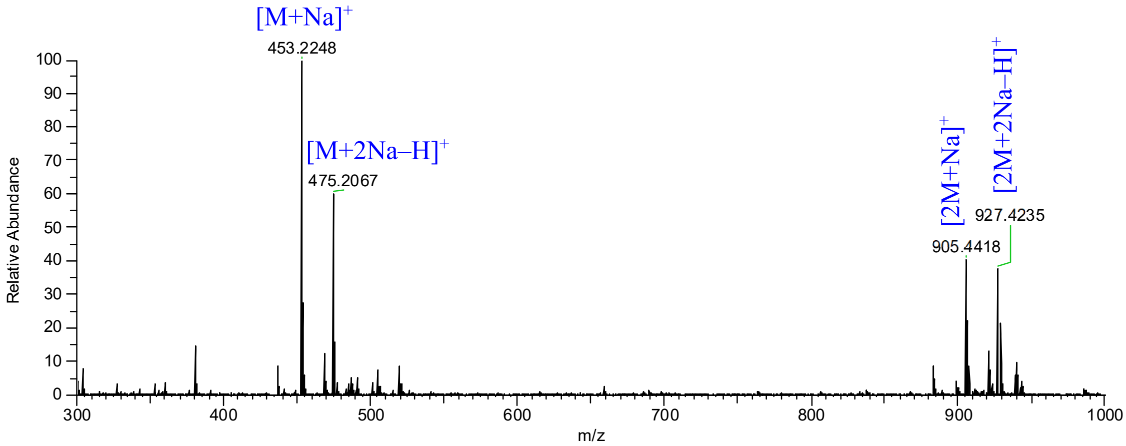

Figure S37. HR-ESI$^+$-MS spectrum of solidagoic acid D (**3**), *m*/*z* 453.2247 $[M+Na]^+$ (calculated for $C_{25}H_{34}O_6Na^+$, *m*/*z* 453.2248 $[M+Na]^+$, error: 0.1 ppm).

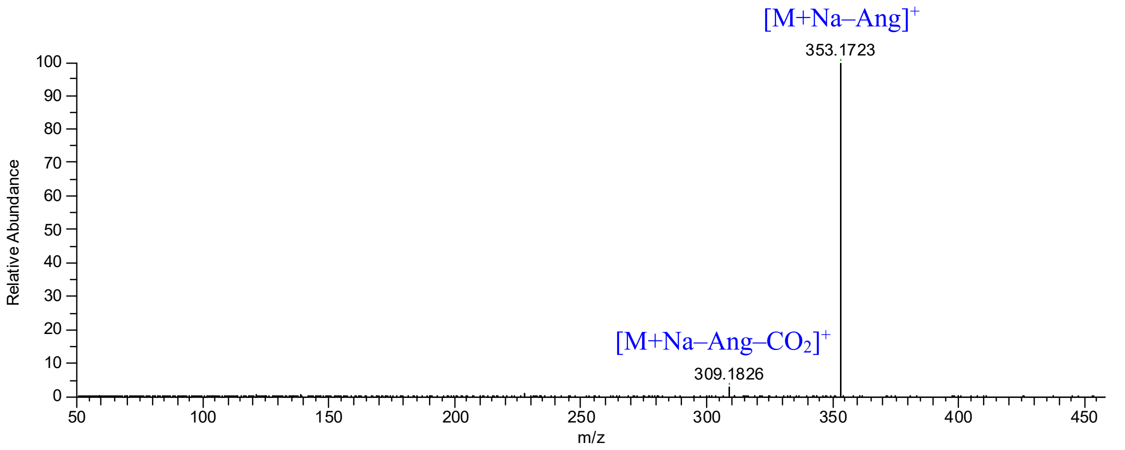

Figure S38. HR-ESI$^+$-MS/MS of solidagoic acid D (**3**) with a normalized HCD collision energy of 20%. Precursor ion: *m*/*z* 453.2248 $[M+Na]^+$, $C_{25}H_{34}O_6Na^+$. Ang denotes an angelate group ($C_5H_8O_2$).

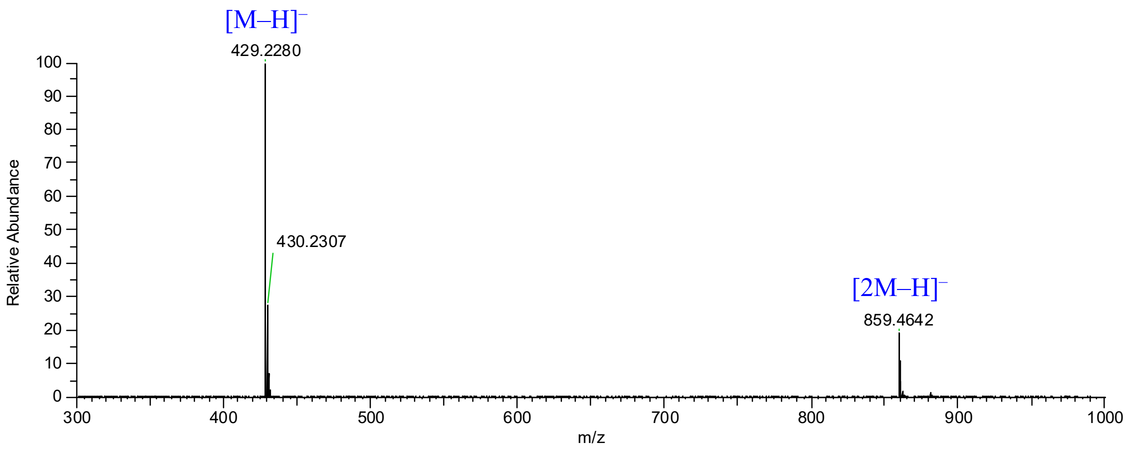

Figure S39. HR-ESI$^-$-MS spectrum of solidagoic acid D (**3**), *m*/*z* 429.2280 $[M-H]^-$ (calculated for $C_{25}H_{33}O_6^-$, *m*/*z* 429.2283 $[M-H]^-$, error: –0.5 ppm).

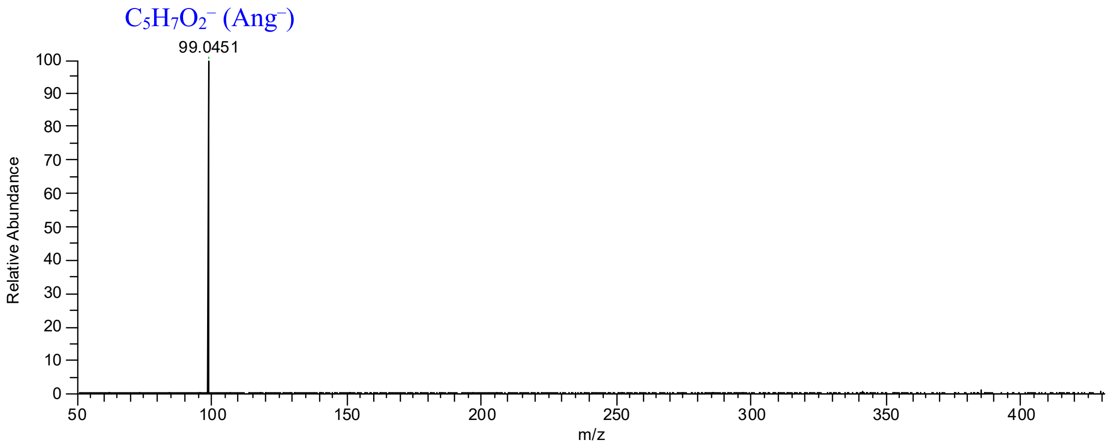

Figure S40. HR-ESI⁻-MS/MS of solidagoic acid D (**3**) with a normalized HCD collision energy of 50%. Precursor ion: *m*/*z* 429.2283 [M–H]⁻, $C_{20}H_{27}O_4^-$. Ang⁻ denotes an angelate ion.

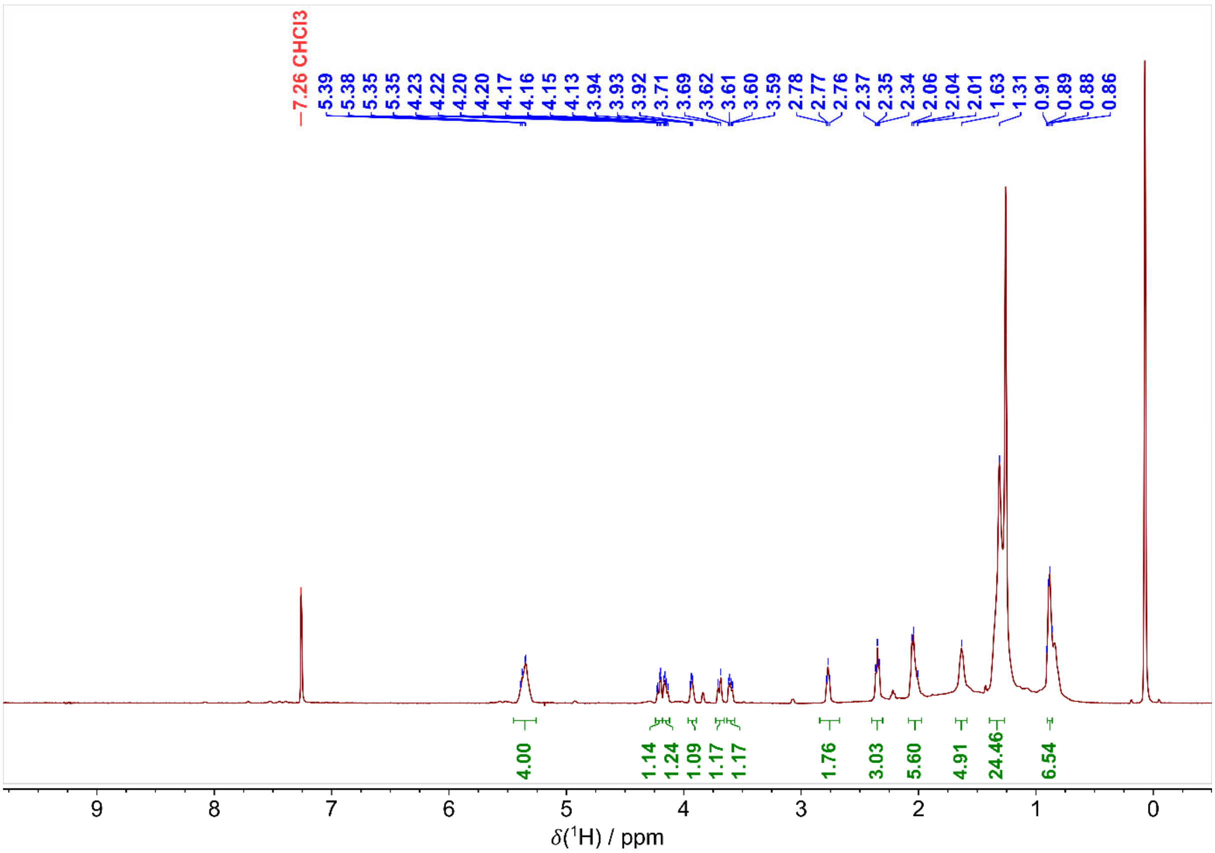

Figure S41. ^{1}H NMR spectrum of 1-linoleoyl glycerol (**4**) ($CDCl_3$, 500 MHz).

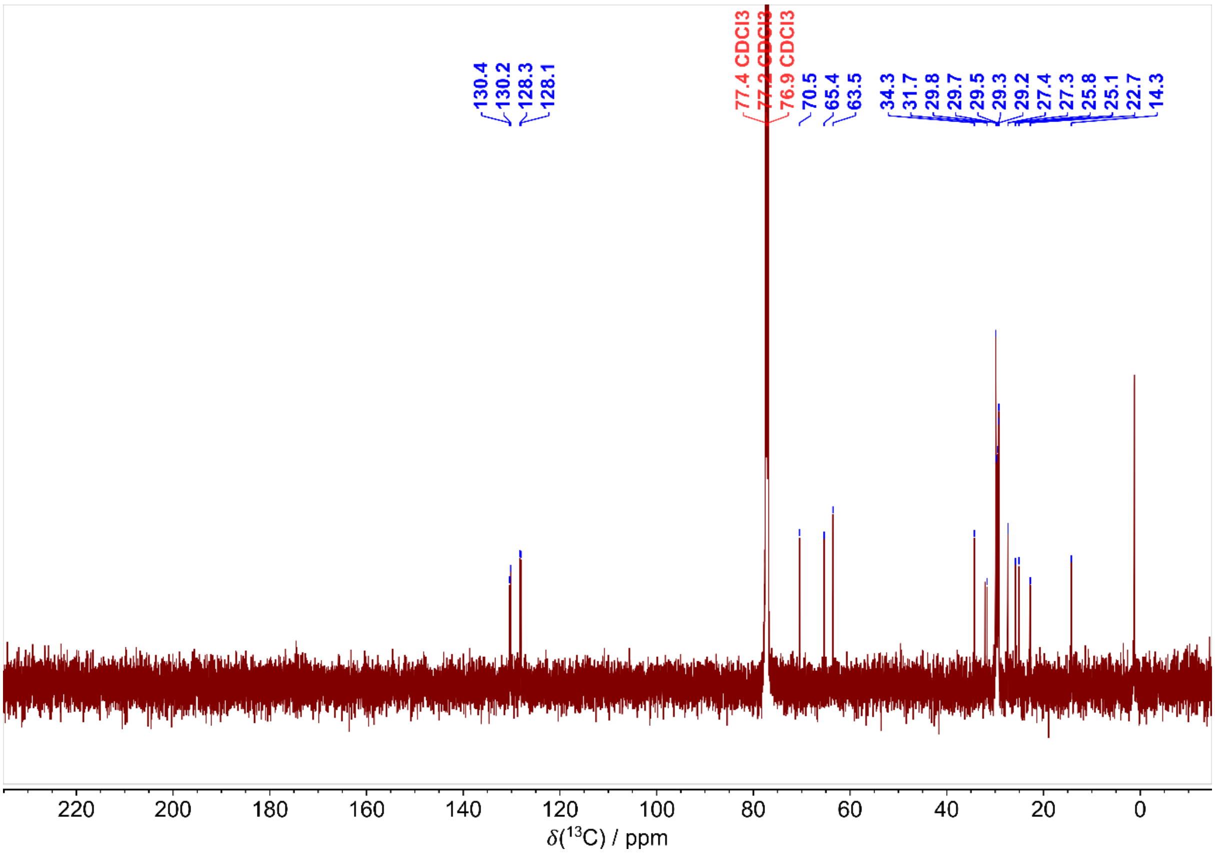

Figure S42. ^{13}C NMR spectrum of 1-linoleoyl glycerol (**4**) ($CDCl_3$, 126 MHz).

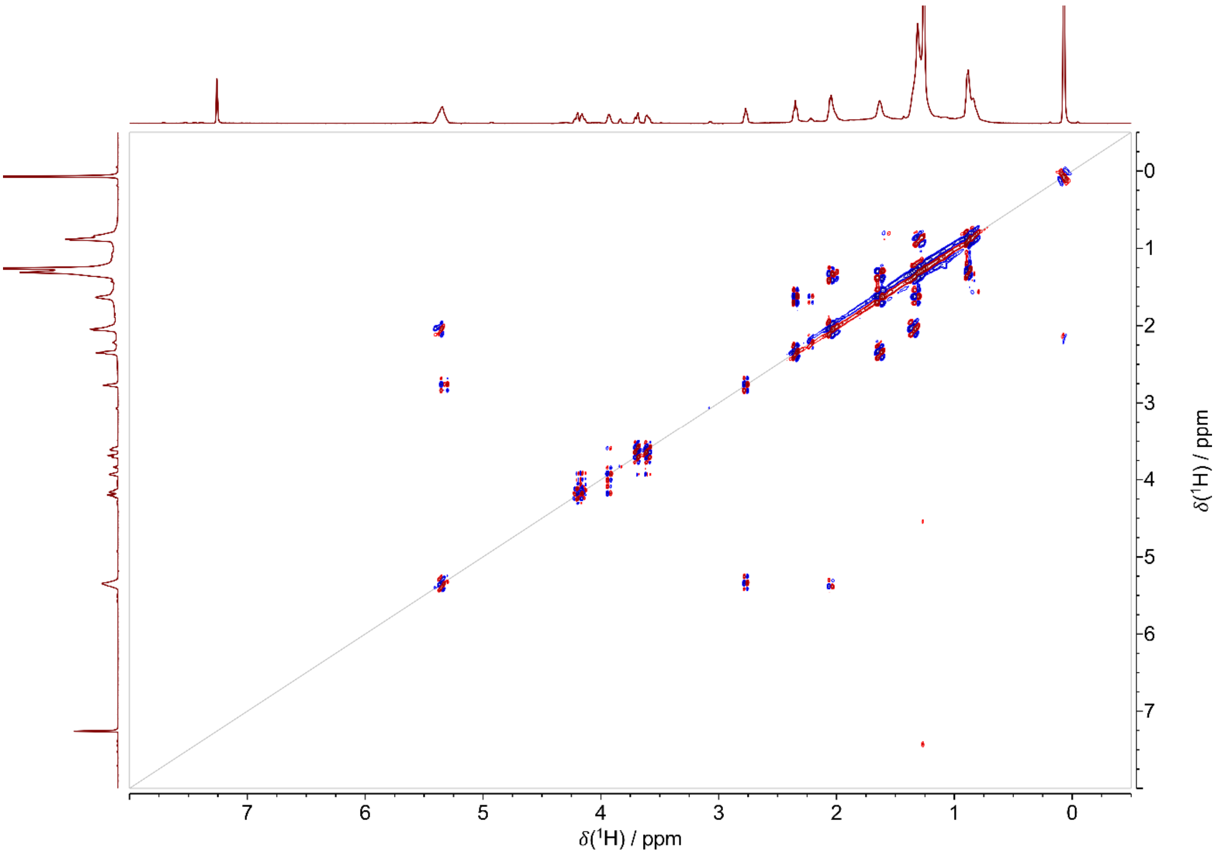

Figure S43. ^{1}H–^{1}H DQF-COSY NMR spectrum of 1-linoleoyl glycerol (**4**) ($CDCl_3$, 500 MHz).

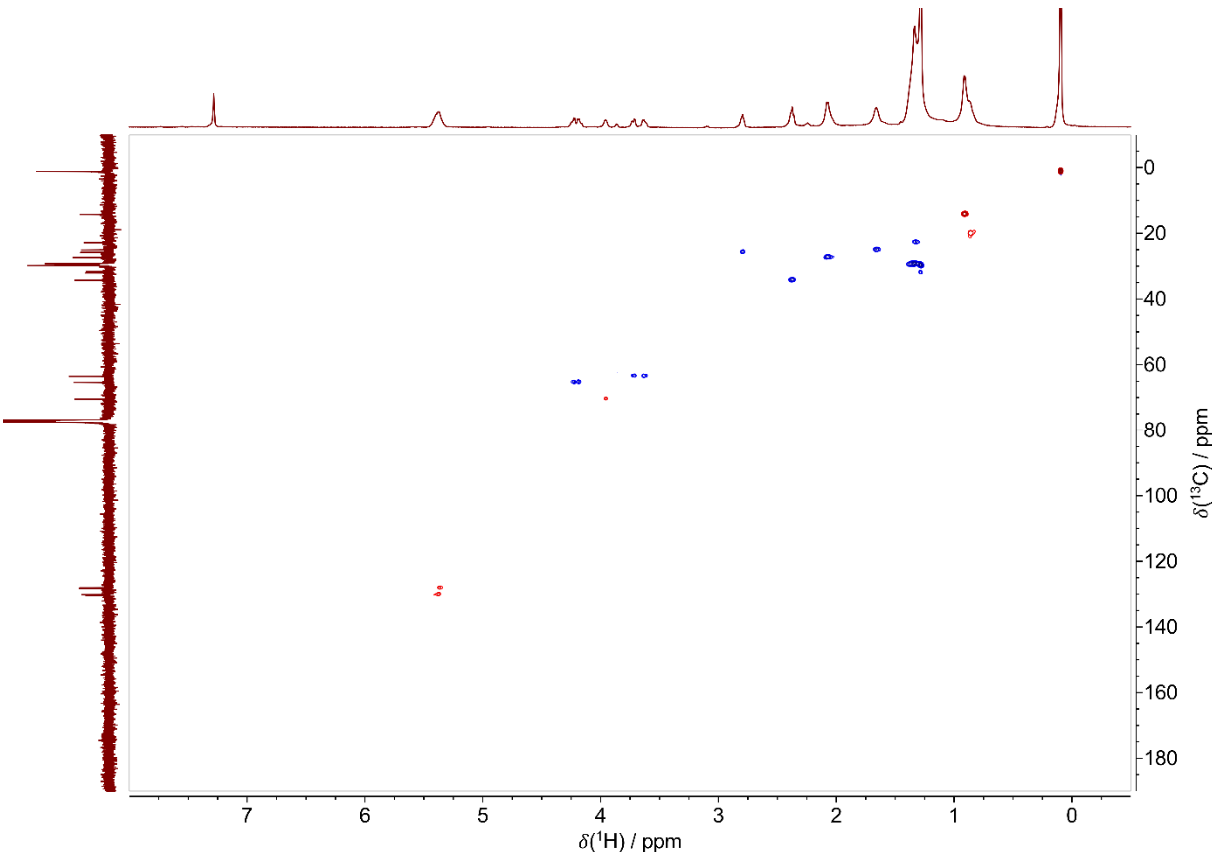

Figure S44. $^{1}H–^{13}C$ edHSQC NMR spectrum of 1-linoleoyl glycerol (**4**) ($CDCl_3$, 500/126 MHz).

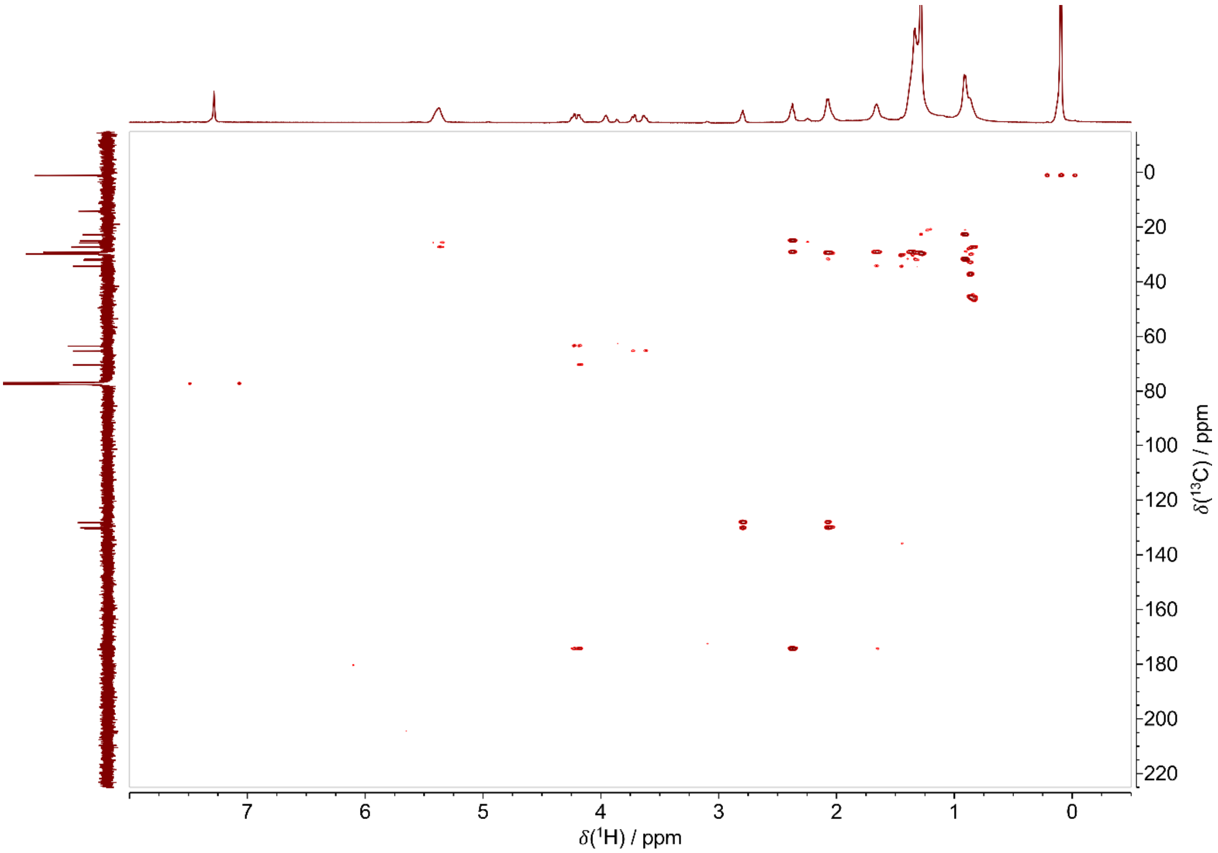

Figure S45. $^{1}H–^{13}C$ HMBC NMR spectrum of 1-linoleoyl glycerol (**4**) ($CDCl_3$, 500/126 MHz).

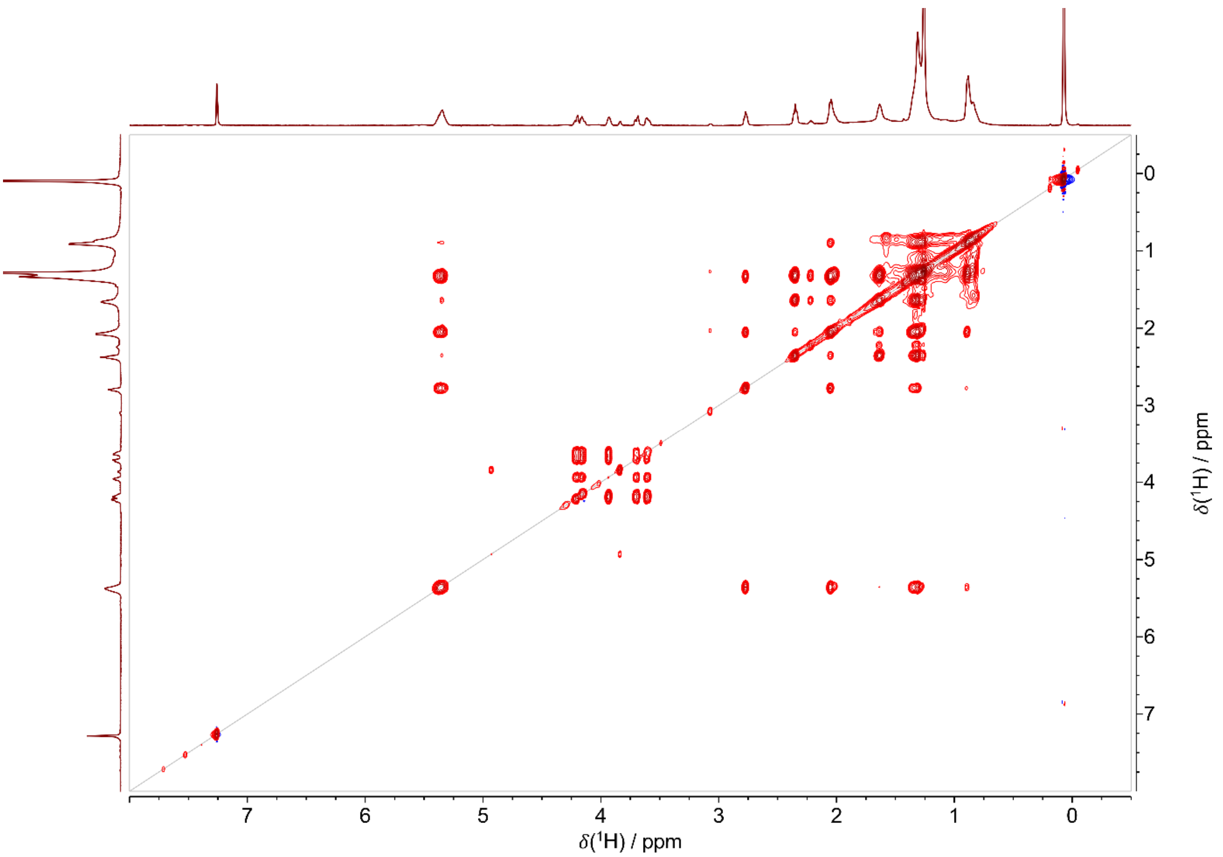

Figure S46. ^1H–^1H TOCSY NMR spectrum of 1-linoleoyl glycerol (**4**) ($CDCl_3$, 500 MHz).

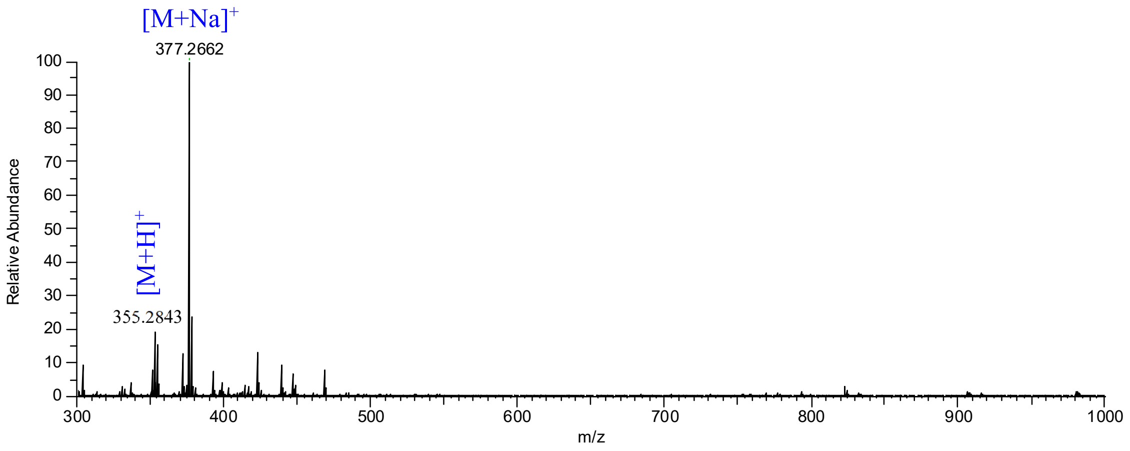

Figure S47. HR-ESI$^+$-MS spectrum of 1-linoleoyl glycerol (**4**), *m/z* 377.2662 $[M+Na]^+$ (calculated for $C_{21}H_{38}O_4Na^+$, *m/z* 377.2662 $[M+Na]^+$, error: 0.0 ppm); *m/z* 355.2843 $[M+H]^+$ (calculated for $C_{21}H_{39}O_4^+$, *m/z* 355.2843 $[M+H]^+$, error: 0.0 ppm).

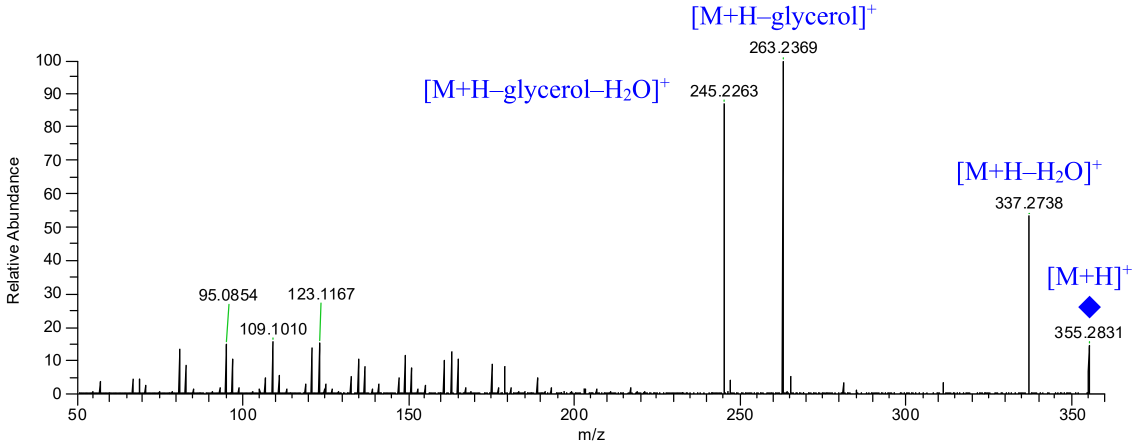

Figure S48. HR-ESI$^+$-MS/MS of 1-linoleoyl glycerol (**4**) with a normalized HCD collision energy of 50%. Precursor ion: *m/z* 355.2831 [M+H]$^+$, $C_{21}H_{39}O_4^+$.

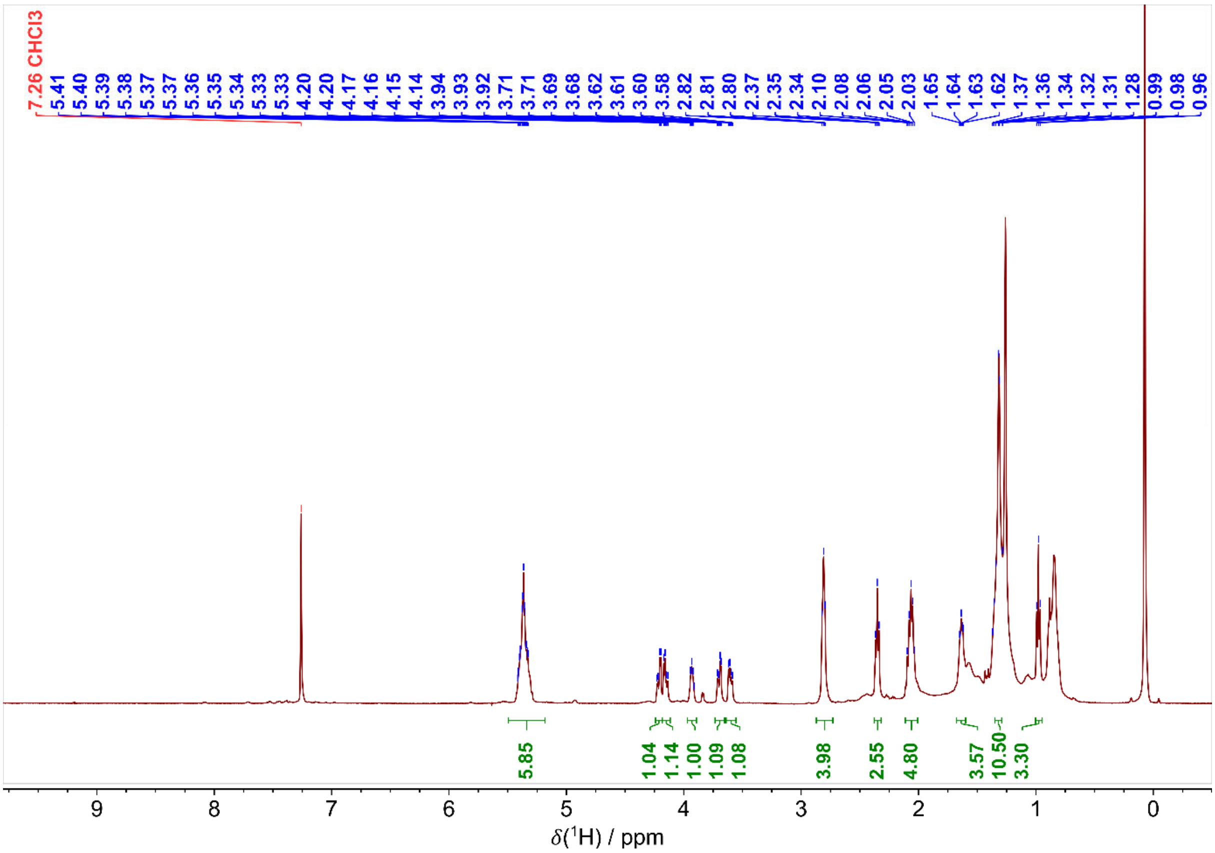

Figure S49. ^{1}H NMR spectrum of 1-α-linolenoyl glycerol (**5**) ($CDCl_3$, 500 MHz).

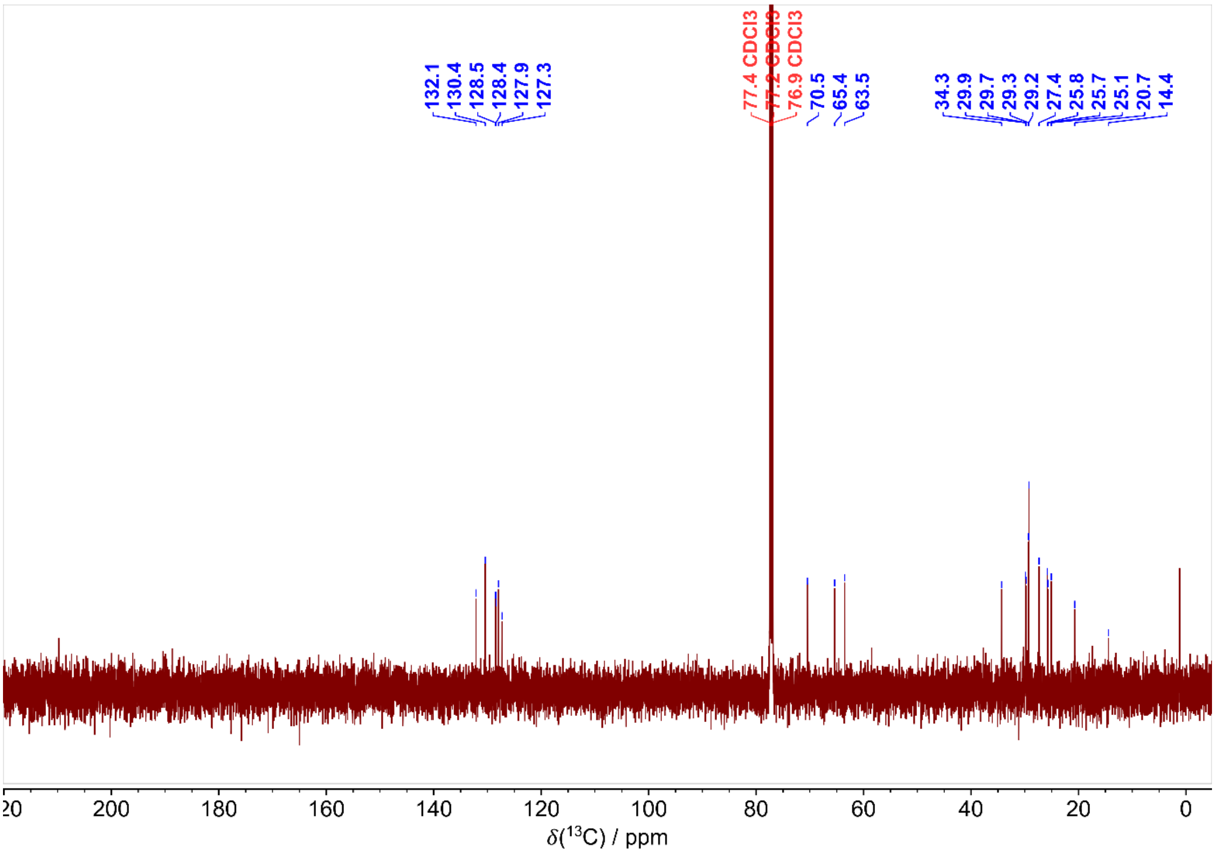

Figure S50. ^{13}C NMR spectrum of 1-α-linolenoyl glycerol (**5**) ($CDCl_3$, 126 MHz).

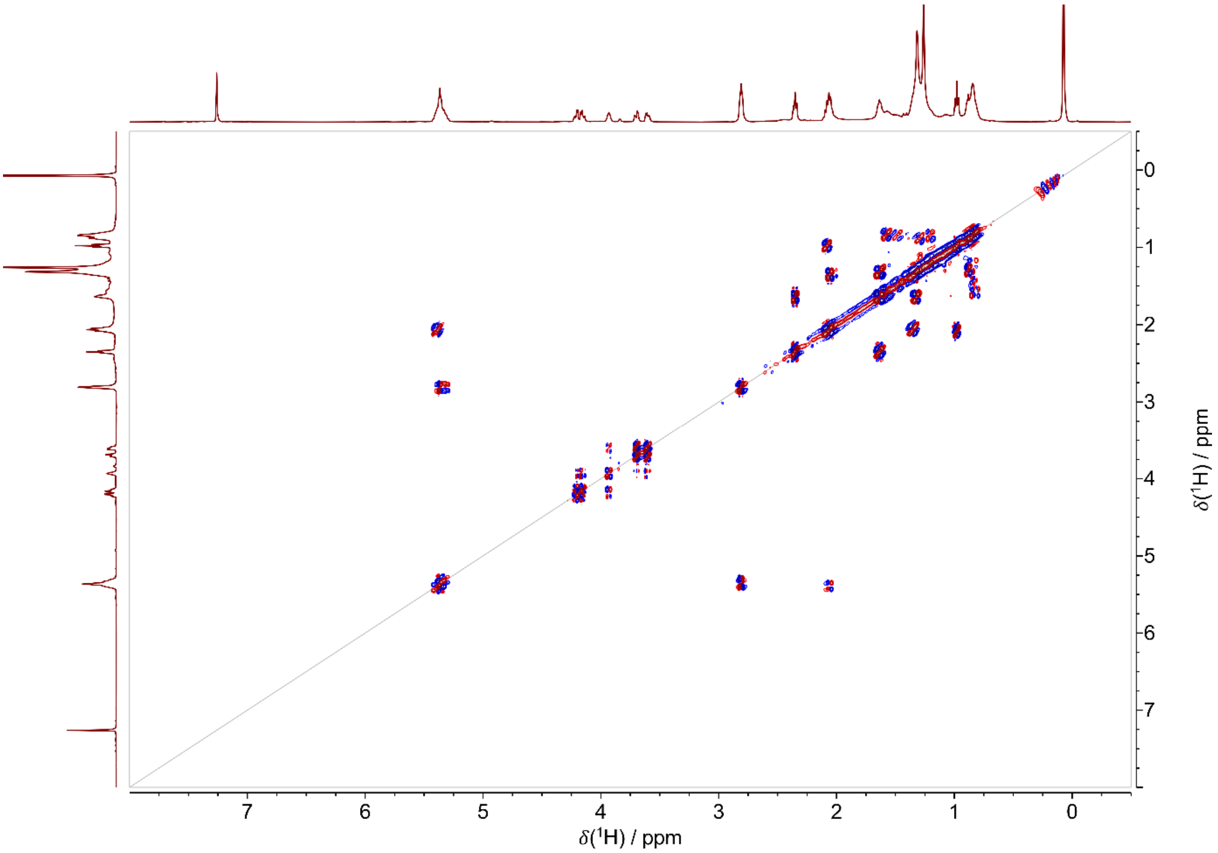

Figure S51. ^{1}H–^{1}H DQF-COSY NMR spectrum of 1-α-linolenoyl glycerol (**5**) ($CDCl_3$, 500 MHz).

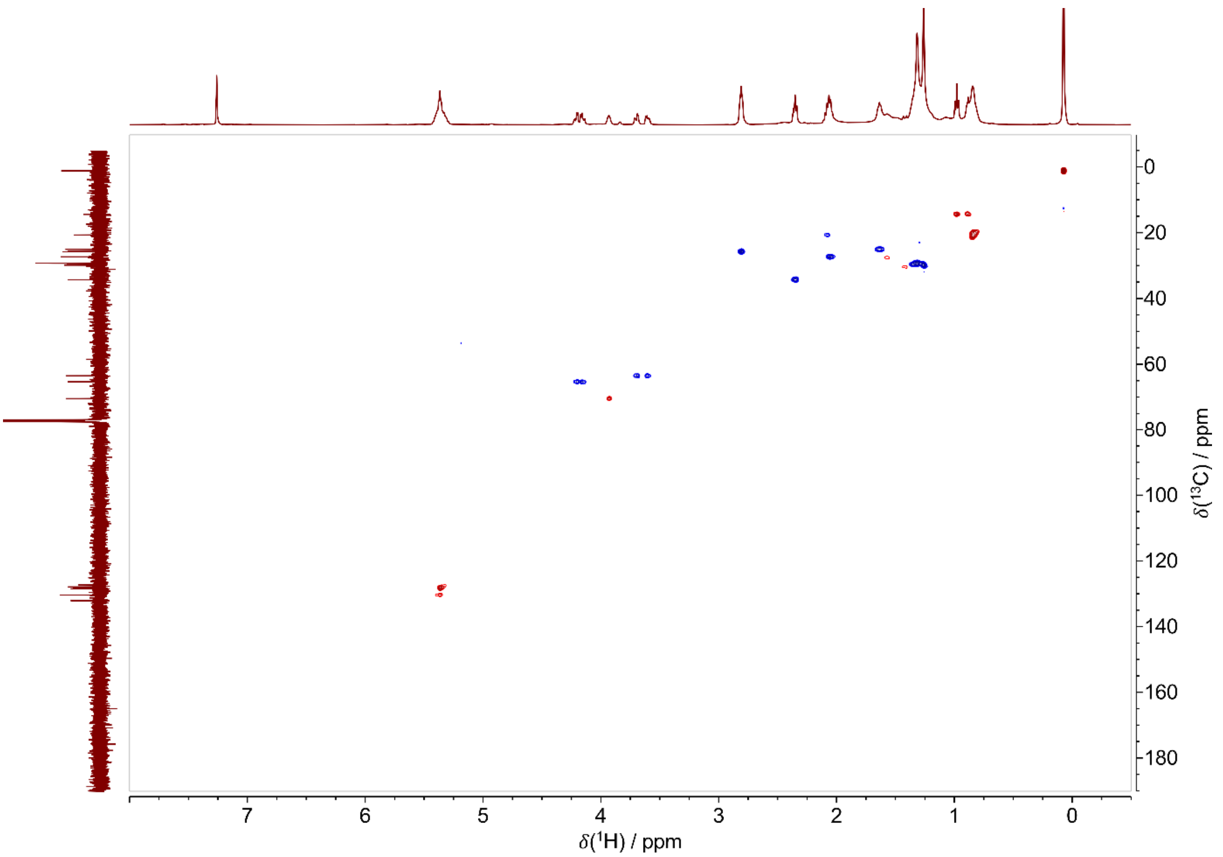

Figure S52. ^{1}H–^{13}C edHSQC NMR spectrum of 1-α-linolenoyl glycerol (**5**) ($CDCl_3$, 500/126 MHz).

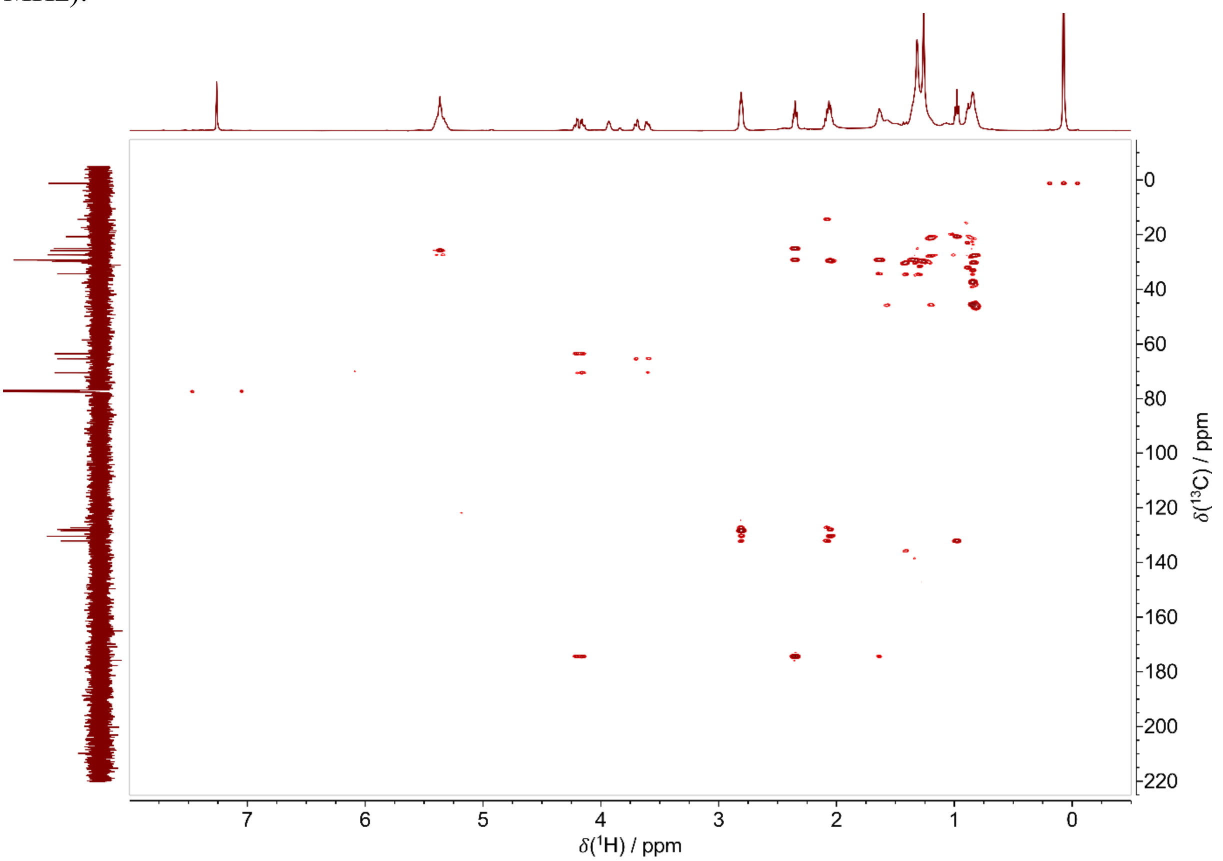

Figure S53. ^{1}H–^{13}C HMBC NMR spectrum of 1-α-linolenoyl glycerol (**5**) ($CDCl_3$, 500/126 MHz).

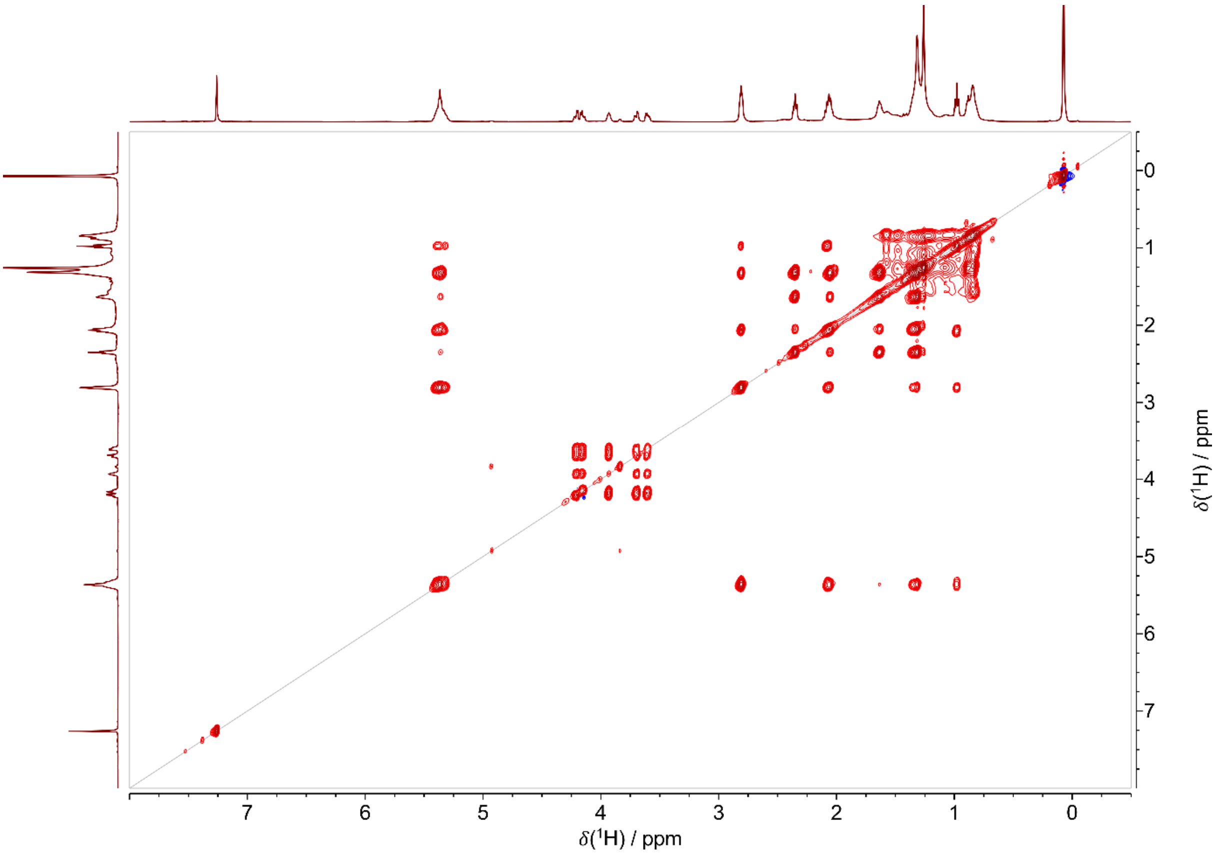

Figure S54. ^{1}H–^{1}H TOCSY NMR spectrum of 1-α-linolenoyl glycerol (**5**) ($CDCl_3$, 500 MHz).

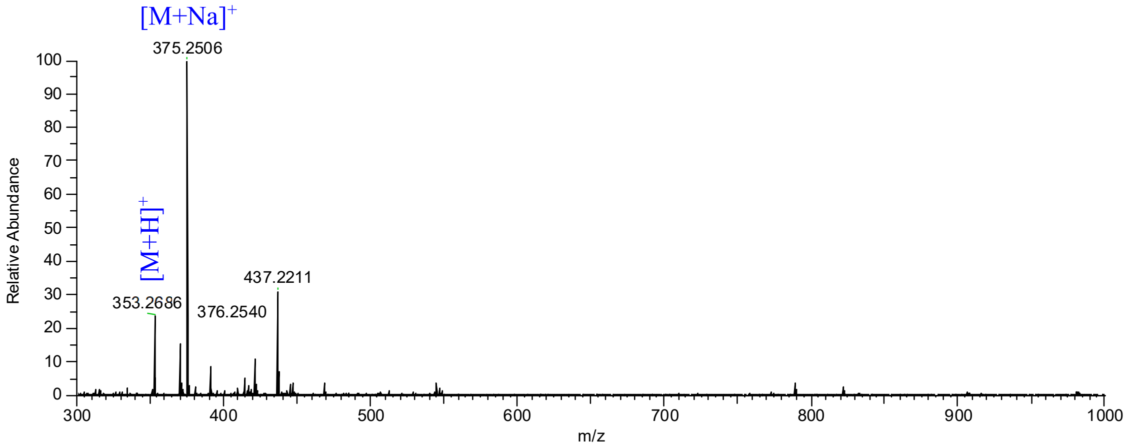

Figure S55. HR-ESI$^+$-MS spectrum of 1-α-linolenoyl glycerol (**5**), *m/z* 375.2506 $[M+Na]^+$ (calculated for $C_{21}H_{36}O_4Na^+$, *m/z* 375.2506 $[M+Na]^+$, error: 0.0 ppm); *m/z* 353.2686 $[M+H]^+$ (calculated for $C_{21}H_{37}O_4^+$, *m/z* 353.2686 $[M+H]^+$, error: 0.0 ppm).

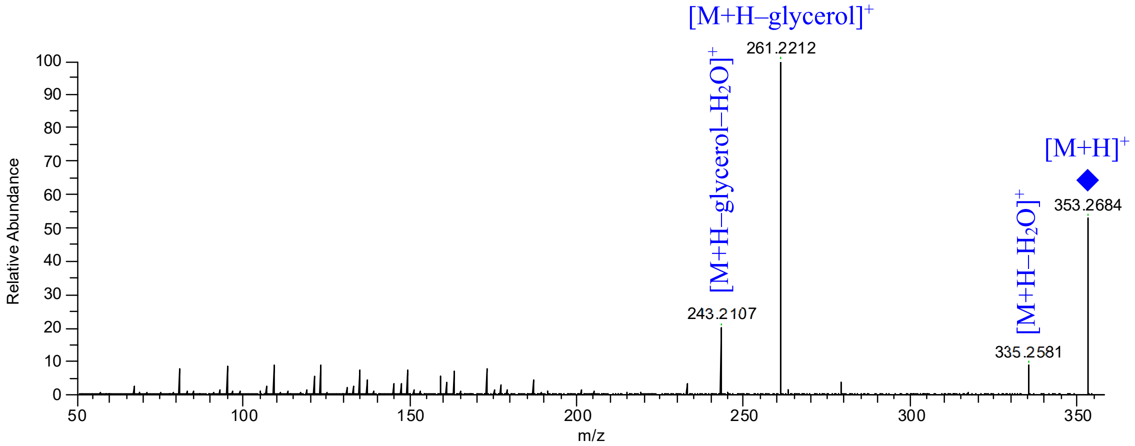

Figure S56. HR-ESI$^+$-MS/MS of 1-α-linolenoyl glycerol (**5**) with a normalized HCD collision energy of 50%. Precursor ion: *m*/*z* 353.2684 $[M+H]^+$, $C_{21}H_{37}O_4^+$.
